# Controlling electric potential to inhibit solid-electrolyte interphase formation on nanowire anodes for ultrafast lithium-ion batteries

**DOI:** 10.1038/s41467-018-05986-9

**Published:** 2018-08-27

**Authors:** Won Jun Chang, Su Han Kim, Jiseon Hwang, Jinho Chang, Dong won Yang, Sun Sang Kwon, Jin Tae Kim, Won Woo Lee, Jae Hyung Lee, Hyunjung Park, Taeseup Song, In-Hwan Lee, Dongmok Whang, Won Il Park

**Affiliations:** 1 Division of Materials Science and Engineering, Hanyang University, Seoul, 04763 Republic of Korea; 2 Department of Chemistry and Research Institute for Convergence of Basic Sciences, Hanyang University, Seoul, 04763 Republic of Korea; 3 Department of Energy Engineering, Hanyang University, Seoul, 04763 Republic of Korea; 4 School of Advanced Materials Science and Engineering, Sungkyunkwan University, Suwon, 16419 Republic of Korea; 5 HYU-HPSTAR-CIS High Pressure Research Center, Hanyang University, Seoul, 04763 Republic of Korea

## Abstract

With increasing demand for high-capacity and rapidly rechargeable anodes, problems associated with unstable evolution of a solid-electrolyte interphase on the active anode surface become more detrimental. Here, we report the near fatigue-free, ultrafast, and high-power operations of lithium-ion battery anodes employing silicide nanowires anchored selectively to the inner surface of graphene-based micro-tubular conducting electrodes. This design electrically shields the electrolyte inside the electrode from an external potential load, eliminating the driving force that generates the solid-electrolyte interphase on the nanowire surface. Owing to this electric control, a solid-electrolyte interphase develops firmly on the outer surface of the graphene, while solid-electrolyte interphase-free nanowires enable fast electronic and ionic transport, as well as strain relaxation over 2000 cycles, with 84% capacity retention even at ultrafast cycling (>20C). Moreover, these anodes exhibit unprecedentedly high rate capabilities with capacity retention higher than 88% at 80C (vs. the capacity at 1C).

## Abstract (web summary)

Lithium-based rechargeable batteries suffer from unstable evolution of solid-electrolyte interphase on the electrode surface. Here, the authors provide an approach to inhibiting SEI formation by controlling electric potential distribution across electrolyte and electrode.

## Introduction

The keys to meeting the ever-increasing demand for highly efficient and ultrafast rechargeable batteries include establishing high-performance electrodes with sufficient energy storage capacity, structural integrity and stability, and fast electron/ion transport^1,2^. As an alternative to conventional graphite anodes, which have an unsatisfactory capacity of 372 mAh g^−1^, higher capacity materials (e.g., Si^3–5^, Ge^6^, and Sn^7^) have been widely explored. However, these materials tend to exhibit substantial capacity fading with cycling, which is primarily due to their large volume change and the evolution of an unstable solid-electrolyte interphase (SEI) on the electrode surface^8,9^. A variety of nanostructured anodes with engineered morphologies and feature sizes can be used to effectively relax the strain caused by extensive volume change; however, this creates a trade-off with SEI stability due to the enlarged surface area of the anode exposed to the electrolyte^10,11^. This becomes more severe for higher capacity materials and with increased cycling rates, making fast and long cycle operation a greater challenge. Recently, to stabilize the SEI on nanostructured anodes, a novel hybrid design concept was developed. Pioneering examples of this concept were implemented by employing a clamping layer on hollow materials (e.g., Si/SiO*x*^12^ nanotubes) or caging nanoscale particles with hollow shell layers (e.g., Si@C^13^ and Ge@C^6^ yolk-shell structures). In those structures, the sturdy shell layers provide a surface for stable SEI formation by preventing direct contact between the electrolyte and the anode, while an internal void space allows for free expansion of the anode material. These approaches led to stable operation with enhanced cycle lifetimes. Nevertheless, the presence of the shell/SEI layer over the large surface of each nanoscale core increases the series resistance for Li-ion and electron transport between the individually engineered nanostructures. This resistance is negligible at slow cycling but becomes problematic under extremely fast cycling^9^.

Given that most electrochemical reactions are governed by a potential difference or gradient, controlling the potential can be explored as an alternative to SEI engineering beyond previous approaches that focused primarily on the physical interface itself^6,7,12,13^. Here, we propose an approach that prevents the formation of an SEI layer on the active anode by engineering the electric potential across the electrochemical interface. As a proof-of-concept for the proposed strategy, we explore anodes with a high density of nickel silicide nanowires (NiSiNWs) anchored selectively to the inner surfaces of graphene-based micro-tubes (GrμTs) (hereinafter referred to as NiSiNWs@GrμT). In this anode design, the electrolyte inside the GrμTs is electrically separated from an external potential load, eliminating the build-up of potential difference that drives SEI formation on the surface of the NiSiNW anode during lithiation. As a result of this electric control, the NiSiNWs@GrμT anode demonstrated excellent performance with a high specific capacity over 700 mAh g^–1^ (corresponding to 84% capacity retention), even after 2000 cycles at 20C. In addition, the capacity fade is markedly reduced with a capacity of 780 mAh g^–1^ at 80C, retaining more than 88% of the capacity at 1C.

## Results

### Strategy to engineering electric potential across the electrochemical interface

**Fig. 1:**
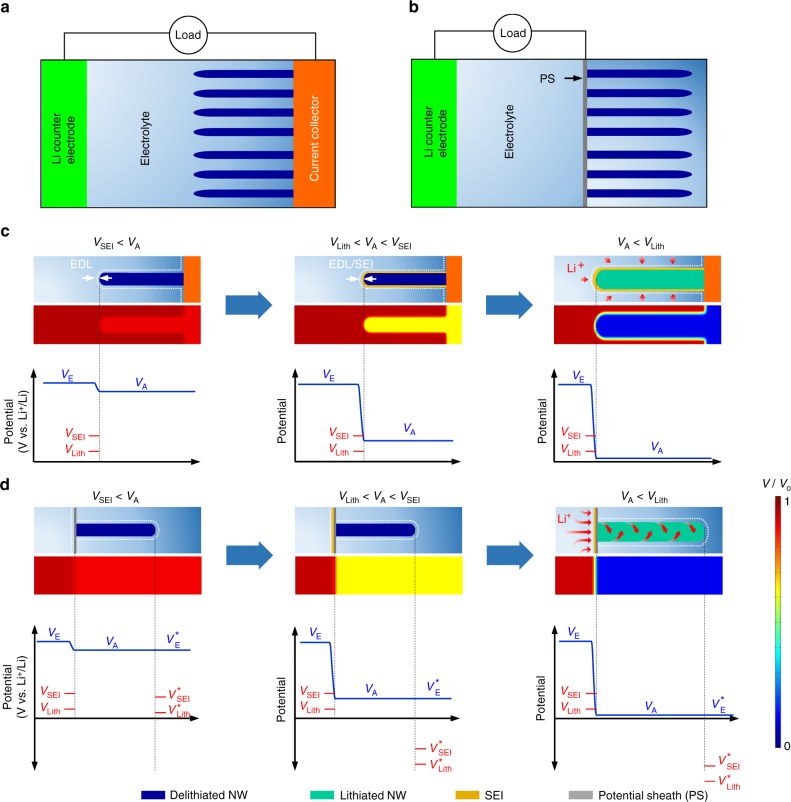
Schematic and electrical potential profiles of the NW-based anodes. **a**, **b** Schematics of a half-cell with a general NW anode on a current collector (**a**) and a half-cell with an NW anode enclosed by PS (**b**) paired with counter electrodes. An external load drives the NW anode to become lithiated. **c** Schematics and corresponding FEA simulation results of electrical potential profiles across the electrolyte and NW anode for the case in **a**, during the first charging cycle. SEI formation and lithiation start at a potential below the *V*_SEI_ and the *V*_Lith_, respectively. **d** Schematic and corresponding electrical potential profiles across the outer electrolyte, electrode (PS, NW anode), and inner electrolyte for the case in **b** during the first charging. Inside the space enclosed by the PS, the potentials across the electrode and electrolyte became nearly equivalent, thereby suppressing SEI formation on the NW surface and lithiation at the electrolyte and NW interface

Figure 1a schematically illustrates the half-cell of the electrolyte and general nanowire (NW) anode on a current collector. Figure 1c shows the change in the structure during the first battery charging and corresponding finite element analysis (FEA) simulation results of the potential distribution across the electrolyte (*V*_E_) and NW anode (*V*_A_) (see also Supplementary Fig. 1). At an early stage of charging, a thin electrical double layer (EDL) develops in the organic electrolyte near the working electrode; most of the potential gradient is created in this area (left and middle panels in Fig. 1c and Supplementary Fig. 1b)^14^. If the working potential of anode (~*V*_A_) becomes substantially lower than the potential of SEI formation (*V*_SEI_), electrons are transported from the anode to the electrolyte and occupy the lowest unoccupied molecular orbital (LUMO) level (middle panel in Fig. 1c and Supplementary Fig. 1c). This process facilitates the reductive decomposition of the electrolyte and the subsequent growth of an SEI layer on the solid surface of the anode^2,15^. When the *V*_A_ further decreases below the potential of lithiation (*V*_Lith_), regular lithiation and concurrent volume expansion occur in the NW anode (right panel in Fig. 1c and Supplementary Fig. 1d)^16^. The SEI on the NW surface also expands and then contracts to its original size upon delithiation. During cycling, with cyclic expansion and contraction of the NWs, the SEI undergoes repetitive breakage and regeneration. This leads to a continuous accumulation of the SEI layer, consumption of the electrolyte, and failure of the anode; this is the primary cause of capacity fading with an increased number of cycles^9^.

To overcome the fundamental limits caused by the SEI, we propose a new design concept introducing a thin wall of a potential sheath (PS) that spatially and electrically separates the electrolyte. Additionally, a high density of NW anodes is anchored to the inside face of the sheath (Fig. 1b). The PS is assumed to be sufficiently thin and lithium (Li)-permeable at *V*_A_ <*V*_Lith_ but electrically conductive so as to possess the functionality of a conducting electrode and an electric shield. In such a scenario, the enclosed space is potentially isolated, in which the potentials of the electrode and electrolyte remain equal (i.e., $V_{\mathrm{A}} = V_{\mathrm{E}}^ \ast$ in thermodynamic equilibrium) regardless of the external bias (left panel in Fig. 1d)^17^. Given that the SEI formation potential across the inner electrolyte and anode interface $\left( {V_{{\mathrm{SEI}}}^ \ast } \right)$, is determined with respect to $V_{\mathrm{E}}^ \ast$ (or LUMO is determined with respect to Fermi level in the electrolyte), $V_{{\mathrm{SEI}}}^ \ast$ remains lower than *V*_A_ (middle and right panels in Fig. 1d). This consideration illustrates that, even at a low working potential of the anode (*V*_A_ < *V*_Lith_), SEI formation on the surface of the NW anode is substantially suppressed. Control experiments support this assertion (Supplementary Figs. 2, 3). As a result of these conditions, an SEI layer develops only on the outer surface region of the PS, where an abrupt potential gradient occurs^2^. The NWs with an SEI-free surface tolerate the large volume change and eliminate the issues related to the SEI (e.g., instability of the SEI and resistance to ion transport)^9^. Although the insertion and extraction of Li ions do not occur via the large area of NW and inner electrolyte interface, the SEI-free NW surface provides a pathway for facile Li transport as suitable for ultrafast (de)lithiation (the details are discussed later).

### Anode based on silicide nanowires anchored inside graphene micro-tubes

**Fig. 2:**
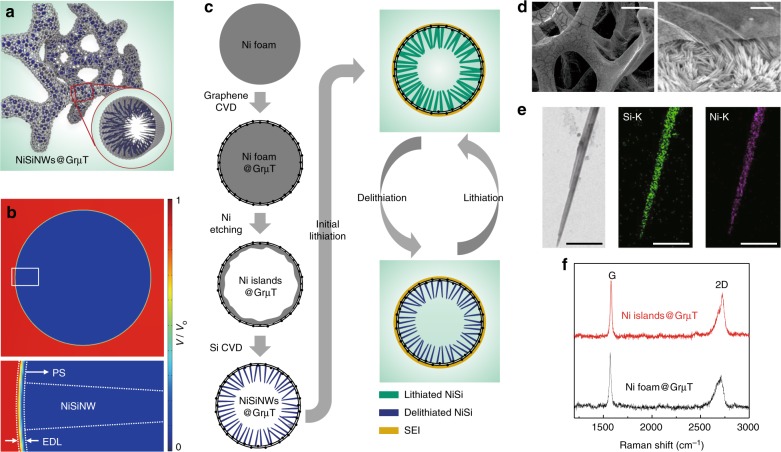
Fabrication and structural analyses of NiSiNWs@GrμT. **a** Schematic of NiSiNWs@GrμT. **b** Simulation of the potential distribution across the NWs@GrμT with an SEI layer on the outer surface of the GrμT. Bottom panel: enlarged images taken from the square in the top panel showing that the formation of the SEI on the outer surface of the GrμT occurred through the development of a rapid potential gradient. A voltage difference of *V*_o_ was applied between the left-hand electrolyte and GrμT, and the calculated position-dependent potential values were normalized with respect to *V*_o_. **c** Schematic of the fabrication process of NWs@GrμT (left) and lithiation-delithiation cycling (right). SEI formation and Li-ion insertion/extraction only occur around the outer surface of the GrμT. **d** SEM images of as-fabricated (left) and cross-sectioned (right) NiSiNW@GrμT. Scale bars, 50 μm (left) and 2 μm (right). **e** TEM image of NiSiNW (left) and EDS elemental mapping images of Si (middle) and Ni (right). A Ni to Si atomic ratio of ~1:1 was confirmed by EDS composition analysis. Scale bars, 400 nm (left) and 250 nm (middle and right). **f** Raman spectra of the GrμT before (black) and after (red) core Ni etching

To test the validity of our proposed concept, we explore anodes with a high density of NWs anchored selectively to the inner surface of a graphene PS with a continuously interconnected, three-dimensional micro-tubular structure (i.e., GrμT), as shown schematically in Fig. 2a. Multilayer graphene with this structure is well-suited for this purpose because it can function effectively as an atomically thin, flexible, robust support with excellent electrical conductivity and electric shielding characteristics. In addition, Li ions/atoms are permeable to the multilayer graphene through atomic defects^18,19^. However, the rate of ion permeation through the GrμT is very slow with regard to the ionic conduction in the electrolyte so that the inner and external electrolytes would be electrically separated. The FEA simulation results showed that more than 97% of the potential gradient develops within the EDL formed on the surface of the graphene PS, while the potential inside the PS remains nearly equivalent to the values of the anode (Fig. 2b and Supplementary Fig. 4 for information about this simulation). It is important to note that our concept can tolerate both anode-electrolyte contact and the existence of nanoscale cracks in the PS (Supplementary Fig. 4b, c). This is different from previous approaches (e.g., yolk-shell structures, in which the internal voids should be kept electrolyte-free by blocking electrolyte permeation with a defect-free sturdy shell); thus, it has advantages in terms of defect tolerance and reliable operation. Alternatively, when the graphene PS has a macroscopic hole, penetration of an external electric potential through the hole countervails the potential shielding effect (Supplementary Fig. 4d).

NiSiNWs are explored as an active anode material; these NWs are attractive due to their potential for high-capacity and good rate capability, as well as their low-temperature growth in the presence of Ni and Si precursors (~450 °C)^20^. NiSi has a gravimetric capacity of ~1300 mAh g^−1^,^21–23^ which is lower than that of Si but still much higher than graphite. An additional advantage of NiSi is related to its metallic conduction, with a typical resistivity of ~10 μΩ cm for single crystals^24^. As schematically shown in Fig. 2c, the key steps to achieve NiSiNWs@GrμT include (i) chemical vapor deposition (CVD) growth of few-layer graphene on Ni foam, (ii) partial etching of core Ni in solution to yield residual Ni islands on the inner surface of the GrμT, and (iii) CVD growth of NiSiNWs. A detailed description of the overall procedure is given in the Methods and scanning electron microscopy (SEM) images of each step and energy-dispersive X-ray spectroscopy (EDS) elemental mapping images of the broken edges of the NiSiNWs@GrμT are shown in Supplementary Figs. 5, 6, respectively.

The SEM image in the left panel of Fig. 2d shows the clean exterior of the final product, which is similar to that of the initial Ni foam (although some shrinkage can be observed). Since the NiSiNWs@GrμT is fabricated with coin-shaped Ni, the GrμT ends remain sealed, as confirmed by SEM images (Supplementary Fig. 5e, f). When the sample was cut after being frozen in liquid nitrogen, very thin graphene tubular walls and a high density of NWs were observed (right panel in Fig. 2d). Typically, the diameter of the GrμT is in the range of 40–60 μm, while the diameter and length of NiSiNWs are ~80–150 nm and 8–10 μm, respectively. To identify the atomic composition and structure of the NiSiNWs, transmission electron microscopy (TEM) and X-ray diffraction (XRD) analyses were performed. The energy-dispersive X-ray spectroscopy (EDS) composition analysis of a single NW reveals the uniform distribution of Ni and Si elements with an atomic ratio of almost 1:1 (Fig. 2e and Supplementary Fig. 7). A lattice-resolved high-resolution TEM image and selected area electron diffraction (SAED) pattern of the NWs also confirm the single-crystal structure of the NiSi core, which coincides with the appearance of XRD peaks of the crystalline NiSi phase after the CVD growth of NiSiNWs, with a thin amorphous shell (Supplementary Fig. 8). From the TEM images of the fragment separated from the NiSiNWs@GrμT, we found that thin island layers remained on the inside surface of the GrμT (Supplementary Fig. 9). The EDS analysis confirmed that those islands are amorphous SiO_X_ embedded with 5–20-nm-diameter Ni nanoparticles. In addition, the Raman spectra of the GrμT collected before and after Ni etching in Fig. 2f exhibit characteristic features, with a 2D to G band ratio in the range of 0.6–0.8, which is similar to the fingerprint of few-layer graphene (five to seven layers) grown on Ni foil (see also Supplementary Fig. 10)^25^. Interestingly, disorder-induced peaks, such as the D peak (~1350 cm^−1^) corresponding to the presence of disorder in *sp*_2_-hybridized carbon systems, are difficult to observe in both samples. These results indicate that the GrμT sustain the original characteristics of few-layer graphene without substantial degradation during Ni etching and NiSiNW growth^26^.

### Electrochemical performance of NiSiNWs@GrμT anodes

**Fig. 3:**
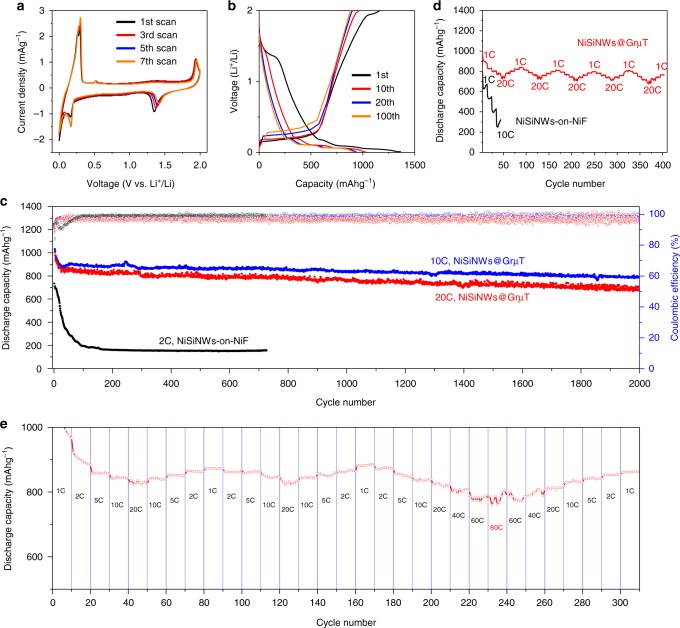
Electrochemical analysis of NiSiNWs@GrμT. **a** CV profiles of NiSiNWs@GrμT during sweeps over the range from 2.0 to 0.0 V vs. Li/Li^+^ at a rate of 0.1 mV s^−1^. **b** Galvanostatic charge-discharge profiles of NiSiNWs@GrμT for the 1st, 10th, 20th, and 100th cycles. **c** Capacity of NiSiNWs@GrμT cycled at the discharging rate while undergoing stepwise changes from 1C to 20C. The NiSiNWs-on-NiF is used for comparison (black). **d** Discharge capacity (solid circles) and coulombic efficiency (open circles) of NiSiNWs@GrμT at 10C (black) and 20C (red) for 2000 cycles. The same plots of NiSiNWs-on-NiF at 2C (blue). **e** Rate capability test of NiSiNWs@GrμT with various cycling rates from 1C to 80C. All tests were performed using 1.0 M LiPF_6_ in EC/DEC (1:1) as the electrolyte, in the potential window between 0.0 V and 2.0 V (vs. Li/Li^+^). All the capacities are represented based on the weight of NiSiNWs

The electrochemical performance of NiSiNWs@GrμT anodes is tested in a CR2032 coin-type cell using Li-metal foil as a counter electrode. Figure 3a shows the evolution of the cyclic voltammetry (CV) profiles for the NiSiNWs@GrμT (1st, 3rd, 5th, and 7th scans). The peaks at 0.1 and 0.22 V in the cathodic scan and at 0.27 V in the anodic scan correspond to lithiation and delithiation of NiSiNWs, respectively, and exhibit increasing current density after the scans. In contrast, other peaks at 1.4 V in the cathodic scan and at 1.9 V in the anodic scan diminish gradually during the scans. We can also observe plateaus at 1.4 and 1.9 V in the galvanostatic charge-discharge (GCD) profiles, displaying similar features in the charging and discharging stages, respectively, and almost disappear after 10–20 cycles (Fig. 3b). The nearly identical behaviors were observed from the nickel sulfide anode^27,28^ and the samples that underwent sulfuric acid treatment (Supplementary Figs. 11, 12)^29^. Thus, we conclude that the plateaus/peaks at 1.4 and 1.9 V are associated with the lithiation and delithiation of nickel sulfide residues that were developed during the nickel etching process using sulfuric acid, respectively. It is noteworthy that the lithiation plateaus disappeared rapidly during the initial several cycles (Fig. 3b). Since the delithiation potential of nickel sulfide shifted toward higher voltage with cycles and exceeded the upper limit of the voltage window (2V)^28^, the more lithiated nickel sulfide could not return to a delithiated state with cycles. This mechanism is closely linked to the rapid capacity fading that tends to occur in NiSiNWs@GrμT anodes in the early stage of cycles (see the red and blue plots in Fig. 3c–e)^30^. Nevertheless, once the anodes were stabilized, there was little change in the charge/discharge profile after 100 cycles at 10C, and the charge/discharge capacities were maintained above 900 mAh g^−1^.

To further evaluate the performance of NiSiNWs@GrμT anodes, charge/discharge cycling tests were performed at 10C and 20C for more than 2000 cycles. The resulting discharge capacity and Columbic efficiency are plotted as a function of the cycle number in Fig. 3c. For comparison, the anode with NiSiNWs-on-Ni foam (NiSiNWs-on-NiF) was also tested at a much slower cycling rate of 2C (see Supplementary Fig. 13 for preparation information about this sample). While the anode with NiSiNWs-on-NiF showed significant capacity fading, with ~33% capacity retention after 100 cycles, the NiSiNWs@GrμT anodes showed superior performance and cycling stability. With the exception of capacity fading during the initial formation cycles (up to the 20th cycle)^30^, the capacity retentions of the NiSiNWs@GrμT anodes are as high as 90% at 10C and 84% at 20C after 2000 cycles. The comparison of the real capacities of NiSiNWs@GrμT with bare GrµT also confirmed that the contribution of graphene to the total capacity was <2% of the total capacity (Supplementary Fig. 14).

Given that changes in the charging/discharging rates can accelerate structural failure of the electrode materials, rate capability is another important consideration for high-power operation. We investigated the rate capabilities of anodes made from NiSiNWs@GrμT and NiSiNWs-on-NiF under a series of stepwise increases and decreases in the discharging rate from 1C to 20C (1C, 2C, 5C, 10C, and 20C; 10 cycles at each rate). As shown in Fig. 3d, even when the charging rate increased 20-fold from 1C to 20C, the NiSiNWs@GrµT anode maintained 91% of its highest capacity at 1C (except for the initial stabilization phase). In contrast, abrupt decay of the charge capacity was observed in the NiSiNWs-on-NiF anode with increasing rates and cycles so that the capacity difference between the anodes became more pronounced with a greater number of cycles. The NiSiNWs-on-NiF anode retained a capacity of 270 mAh g^−1^ at 10C, which is <34% of the capacity of the NiSiNWs@GrμT. More importantly, NiSiNWs@GrμT anodes demonstrate excellent rate capability under more severe cycling conditions, with a stepwise increase and decrease in the rate from 1C to 20C and from 1C to 80C, respectively (Fig. 3e). The anode maintained a capacity above 800 mAh g^−1^ at 40C and above 780 mAhg^−1^ at 80C, retaining 90 and 88% of the capacity at 1C, respectively. The comparison of these results with state-of-the-art LIB anodes in the literature illustrates the substantial advantages of the NiSiNWs@GrμT anode (Supplementary Fig. 15). Importantly, only a few reports have addressed the cycling performance of anodes under extreme cycling conditions and charge/discharge rates higher than 10C^31^. To the best of our knowledge, there is no report demonstrating a capacity higher than 780 mAh g^−1^ at speeds higher than 80C.

## Discussion

**Fig. 4:**
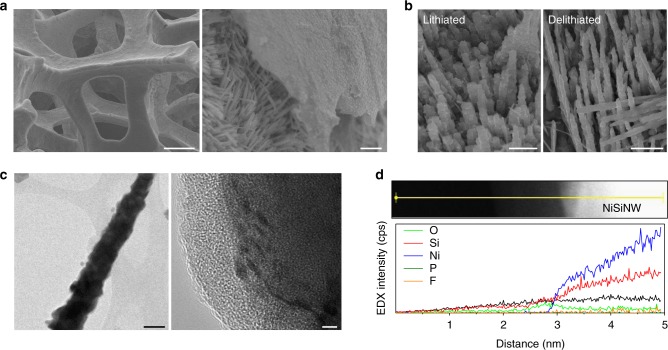
Morphology and SEI formation of NiSiNWs@GrμT after cycling. **a** SEM image of delithiated NiSiNWs@GrμT after cycling. Close-up SEM image of the intentionally-fractured part of the NiSiNWs@GrμT (right), which shows near SEI-free NW surfaces inside the GrμT covered with a thick SEI layer. Scale bars, 100 μm (left) and 2 μm (right). **b** SEM images of the lithiated (left) and delithiated (right) NWs inside the GrμT (with the same magnification). The diameter of the NWs increases by an average of 190% after lithiation. Scale bars, 1 μm. **c** TEM image of delithiated NiSiNW after cycling (left) and enlarged HR-TEM images taken from the square in the left panel, showing the polycrystalline NiSi core covered with a 5-nm-thick oxide amorphous shell (right). 100 nm (left) and 2 nm (right). **d** EDS element analysis of delithiated NiSiNW, taken along the solid line in the upper panel of the dark-field image of the NiSiNW and oxide region. SEI-related components (e.g., P and F) were not detected

We ascribe the unprecedentedly superior cycling performance of the NiSiNWs@GrμT anode to the synergetic advantages of the GrμT and NiSiNWs; an SEI layer develops only on the outer surface of the GrμT-based PS, and the SEI-free NiSiNWs allow fast electron/ionic transport and reversible volumetric change. SEM images of the NiSiNWs@GrμT taken after cycling confirmed that the NiSiNWs maintained most of their original shape and size, although they did show a roughened surface (Fig. 4a, b). The selective formation of an SEI layer on the outer surface of the GrμT, which agrees with our hypothesis, was also confirmed (Fig. 4a, right panel). The comparison of the lithiated and delithiated NiSiNWs showed that the diameter increased by an average of 190%, which is comparable to the theoretical expectation (Fig. 4b)^32^. TEM images of a single delithiated NW, detached from the GrμT, showed a polycrystalline core and amorphous shell (Fig. 4c); these can be assigned to NiSi and SiO*x* (NiO*x*), respectively, based on EDS elemental analysis (Fig. 4d and Supplementary Fig. 16). Importantly, the P and F associated with the SEI layer component are hardly detected in the NiSiNWs^33^. In contrast, the EDS peaks corresponding to F and P elements are clearly seen for the outer surfaces of the GrμT (Supplementary Fig. 17a, b). These results support the validity of our approach, which is based on electric potential control to influence SEI development. In the case of the NiSiNWs-on-NiF anode, all of the NWs are embedded with a thick SEI layer after 100 cycles; the individual NW morphology can be identified only after removing the SEI with acetonitrile (Supplementary Fig. 17c and 18)^34^. This result is also coincident with FEA modeling that shows the existence of an abrupt potential drop on the surface of the NiSiNWs (Supplementary Fig. 4e). Although we cannot exclude the contribution of residual Ni nanoparticles (Supplementary Fig. 9) to the improved rate performance, our in-depth analysis and consideration illustrate that the ‘potential shielding’ effect has a major role in achieving high rate capability.

Despite the excellent cyclability of the NiSiNWs@GrμT, there were large fluctuations of capacity and coulombic efficiency at higher rates (>20C); the coulombic efficiency occasionally exceeded 100% (Fig. 3c, e). We attribute this to the limited Li diffusion in the NWs during ultrafast cycling, which prevents complete lithiation/delithiation^35^. For instance, the residual Li in the delithiated state would reduce the charge capacity in the next cycle, whereas these Li remnants can be released during the next discharging stage, causing the discharge capacity to exceed the charge capacity. For Li diffusion, the NWs inside the PS would have a disadvantage considering that the potential gradient that drives the lithiation does not develop at the NW and electrolyte interface. However, previous in situ TEM studies showed fast lithiation kinetics in individual NWs, in which Li insertion occurred at one end of the NWs and was followed by Li transport along the axial direction, similar to our situation. In some cases, the overall lithiation rate was not axial transport limited, but rather determined by the radial insertion of Li into the NW core^36^. This is due to the much faster Li transport along the surface than that in the bulk, enabling ultrafast lithiation with an axial speed up to ~213 nm s^–1^ for highly conducting SiNWs^37^. In this regard, we conclude that the Li diffusion in our NW anode does not critically limit the cycling capacity even at higher rate (e.g., the axial lithiation speed of 200 nm s^–1^ can fully lithiate ~8 μm-long SiNWs at 80C). Nonetheless, further study is needed to elucidate the lithiation kinetics in our system.

In summary, we suggest a new strategy for inhibiting SEI formation on the anode surface through engineering the electric potential using an atomically thin and ion-permeable PS. The NiSiNWs@GrμT were tested as a proof-of-concept for the proposed strategy and demonstrated excellent performance during 2000 cycles at 20C with a high specific capacity of 702 mAh g^–1^, corresponding to 84% of the initial capacity. Moreover, the NiSiNWs@GrμT anodes showed superior rate capabilities with a capacity retention higher than 88% at 80C (vs. the capacity at 1C). Because our approach is based on general electrochemistry, it is applicable to other materials (e.g., Si-based anode) to further improve their capacity. It can also be used with a variety of electrochemical devices and components.

## Methods

### Fabrication of NiSiNWs@GrμT

The NiSiNWs@GrμT were fabricated by growing NiSiNWs selectively inside the graphene-based 3D micro-tubular structures. First, high-purity Ni foam (1 mm in thickness) was purchased from MTI Korea and cut into coin-like shapes using a punching machine to facilitate cell assembly. Next, the Ni foam was loaded in the CVD reactor chamber to synthesize multilayer graphene on the surface of the Ni foam. The reactor was evacuated below 5 × 10^–3^ torr and heated to 1000 °C. After annealing the Ni foam for 30 min under H_2_/Ar atmospheric pressure, a gas mixture containing 30% CH_4_ diluted in H_2_ and Ar was introduced into the reactor to initiate graphene growth. After 2 min of exposure to the precursor gas mixture, the reactor was evacuated again and immediately cooled to room temperature. The color change of the Ni foam after uniform coating with multilayer graphene was recognizable to the naked eye. The sample was treated with ethanol (EtOH) and then immersed into a Ni etchant (UN2796 sulfuric acid, Transene Company, Inc.), which acts as a surfactant to prevent the formation of air bubbles inside the sample. Samples were immersed in the Ni etchant for 2.5 h, until only a small amount of Ni (0.45 mg cm^–2^) remained inside the GrμT. The sample was then rinsed three times with deionized water and dried fully in an oven (70 °C). After complete drying, the sample was loaded into a silicon CVD reactor to grow NiSiNWs. The growth of NiSiNWs occurs selectively inside the GrμT in the presence of the residual Ni and Si precursors of SiH_4_. During NiSiNW growth, a gas mixture of 10% SiH_4_ diluted with H_2_ was introduced at a flow rate of 50 sccm, and the reactor temperature and pressure were maintained at ~460 °C and 20 torr, respectively. The typical growth time was 20 min. A reference anode consisting of NiSiNWs directly grown on Ni foam was also prepared using identical Si CVD processing conditions.

### Structural characterization

For structural and elemental analyses of the samples, FE-SEM (JEOL JSM-7600 with 15 kV), TEM equipped with EDS (JEOL JEM-2100F, Cs corrector), Raman spectroscopy (MonoRa 750i, Dongwoo Optron Co.), and XRD (Rigaku D/MAX RINT-2000) were used. For cross-sectional SEM measurements, the NiSiNWs@GrμT samples were cut after being frozen in liquid nitrogen. After cycling, the NiSiNWs@GrμT anode was rinsed three times with the EC/EDC solvent and then dried in a glove box.

### Electrochemical characterization

NiSiNWs were assembled into coin-type half cells (CR2032) in an argon-filled glove box. Li metal was used as a counter electrode, and 1.0 M LiPF_6_ in ethylene carbonate/diethylene carbonate (EC/DEC, 1:1 vol.%) was used to fill the half cells. CV profiles were determined using an electrochemical workstation (IVIUM-n-STAT). CV was performed at a scan rate of 0.1 mV s^–1^ between 0 and 2 V. All of the GCD profiles and rate/cycling performances of the coin-type cells were tested using a TOSCAT 3000 battery tester (TOSCAT 3000, Japan) between 0.01 and 2 V.

### Data availability

The authors declare that all the relevant data are available within the paper and its Supplementary Information file or from the corresponding author on reasonable request.

## Electronic supplementary material

Supplementary Information

Peer Revew File

Description of Additional Supplementary Files

Supplementary Movie 1

Supplementary Movie 2
