# Supplementary material for: Controlling electric potential to inhibit solid-electrolyte interphase formation on nanowire anodes for ultrafast lithium-ion batteries
Source: Nat Commun. 2018 Aug 27;9:3461. doi: 10.1038/s41467-018-05986-9 (PMC6110779; doi:10.1038/s41467-018-05986-9)
Supplement: Supplementary file 1 — Supplementary Information [file 41467_2018_5986_MOESM1_ESM.pdf]

Supplementary Information

Controlling Electric Potential to Inhibit Solid-Electrolyte Interphase Formation on Nanowire Anodes for Ultrafast Lithium-ion Batteries

Chang, *et al.*

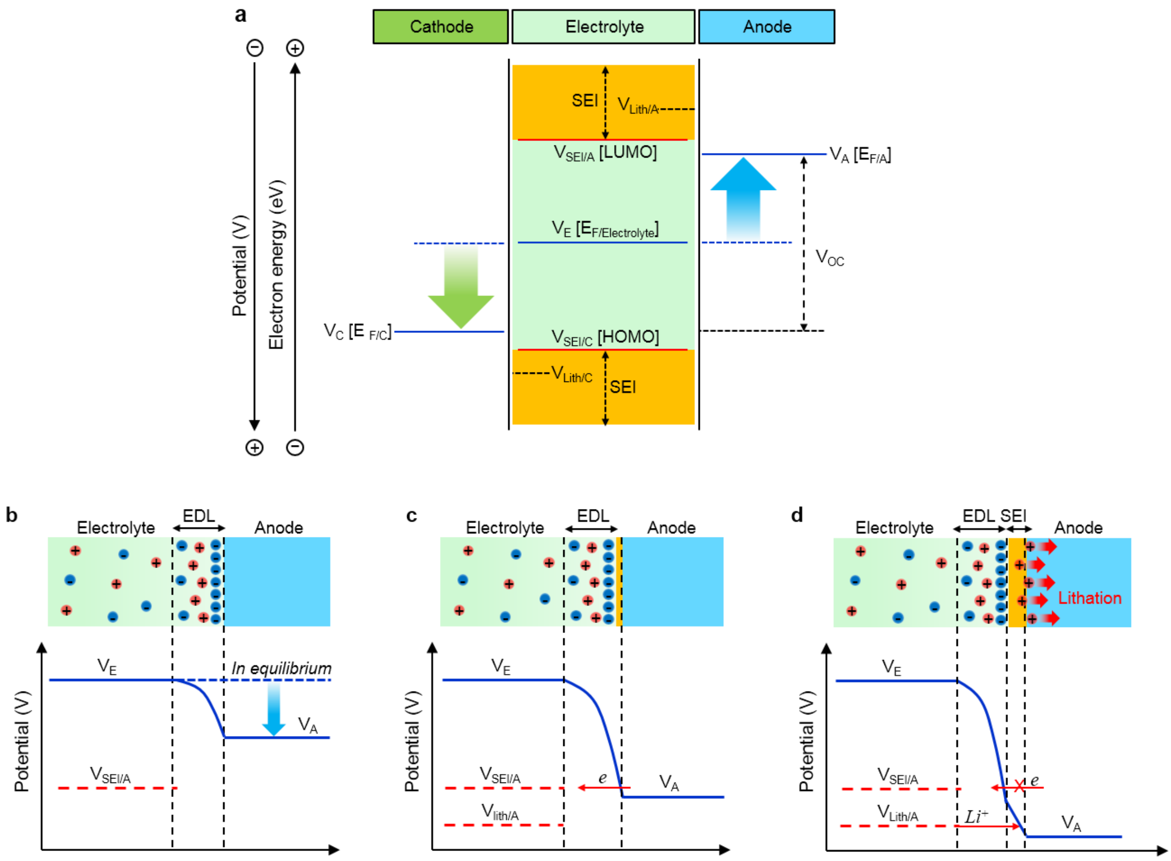

Supplementary Figure 1. Potential/energy diagrams across a battery cell. **a,** Schematic potential and energy diagram across a lithium ion battery cell, consisting of a cathode, electrolyte and anode. Potentials of the cathode (V_C), electrolyte (V_E) and anode (V_A) correspond to their own Fermi levels (or electrochemical potential of electrons) of $E_{F/C}$, $E_{F/Electolyte}$, and $E_{F/A}$, respectively, in the energy scale[1]. The $V_{Lith/C}$ and $V_{Lith/A}$ refer to the (de)lithiation potential of the cathode and anode, respectively. During the charging, the V_C and V_A move in opposite directions, establishing open circuit voltage (V_{OC}). In a cathode, SEI formation starts at a potential V_C above the $V_{SEI/C}$ (or the $E_{F/C}$ lies below the HOMO); in an anode, SEI formation starts at a V_A below the $V_{SEI/A}$ (or the $E_{F/A}$ lies above the LUMO). Similarly, delithiation starts at the V_C above the $V_{Lith/C}$ in the cathode while lithiation starts at the V_A below the $V_{Lith/A}$ in the anode. **b–d,** Schematic images illustrating the developments of EDL and SEI layers and corresponding potential profiles across the electrolyte and anode, during the first charging cycle. During charging, the V_A moves downward, establishing a rapid potential gradient across a very thin EDL layer (b). When the V_A lies below the $V_{SEI/A}$, the electrolyte will be reduced near the anode surface and a thin passivation SEI layer will develop on the anode surface until the SEI layer prevents electron transfer from the anode to the electrolyte LUMO (c). At a potential V_A below the $V_{Lith/A}$ regular lithiation occurs involving the transport of Li^+ to the anode surface through ion-permeable SEI and thereafter reduction and insertion into the anode (d).

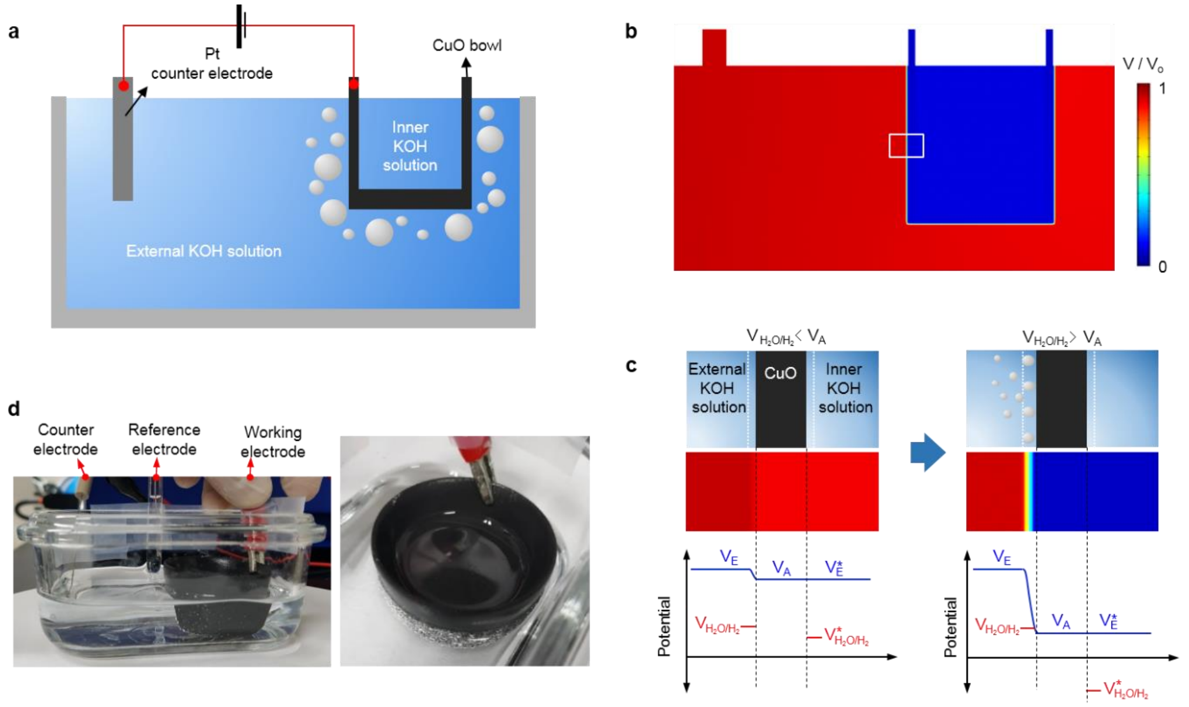

Supplementary Figure 2. Controlled experiment I illustrating electric potential control of the hydrogen evolution reaction. a, Schematic of the experimental setup of the electrochemical cell, consisting of a Pt counter electrode and a copper (Cu) bowl coated with a thermally oxidized layer (CuO) on both the outer and inner surfaces; electrodes are exposed to a 1.0 M KOH aqueous electrolyte. An external load drives the hydrogen-evolution reaction on the CuO/Cu anode surface. **b**, FEA simulation of the potential distribution across the cell under an external voltage bias (V_o). **c**, Schematic and corresponding electrical potential profiles across the electrolyte and CuO/Cu anodes, taken from the square in **b**, without (left) and with (right) an external potential bias. When the working potential of the anode (V_A) becomes substantially lower than the potential of the hydrogen redox potential (V_{H_2O/H_2}) caused by an external bias, hydrogen-evolution starts; however, as confirmed by the generation of hydrogen gas bubbles, the hydrogen-evolution reaction occurs only on the outer surface of the CuO/Cu anode. Inside the space enclosed by the CuO/Cu anode, the potential gradient across the electrode and electrolyte remains constant, thereby suppressing the hydrogen-evolution reaction. **d**, Photograph of the controlled experiment showing the generation of hydrogen bubbles on the outer surface of the CuO/Cu bowl (see also Supplementary Movie 1). This control experiment shows that the hydrogen-evolution reaction can be initiated or inhibited via electric potential control using the Cu bowl as a conducting sheath; this demonstrates the validity of our approach.

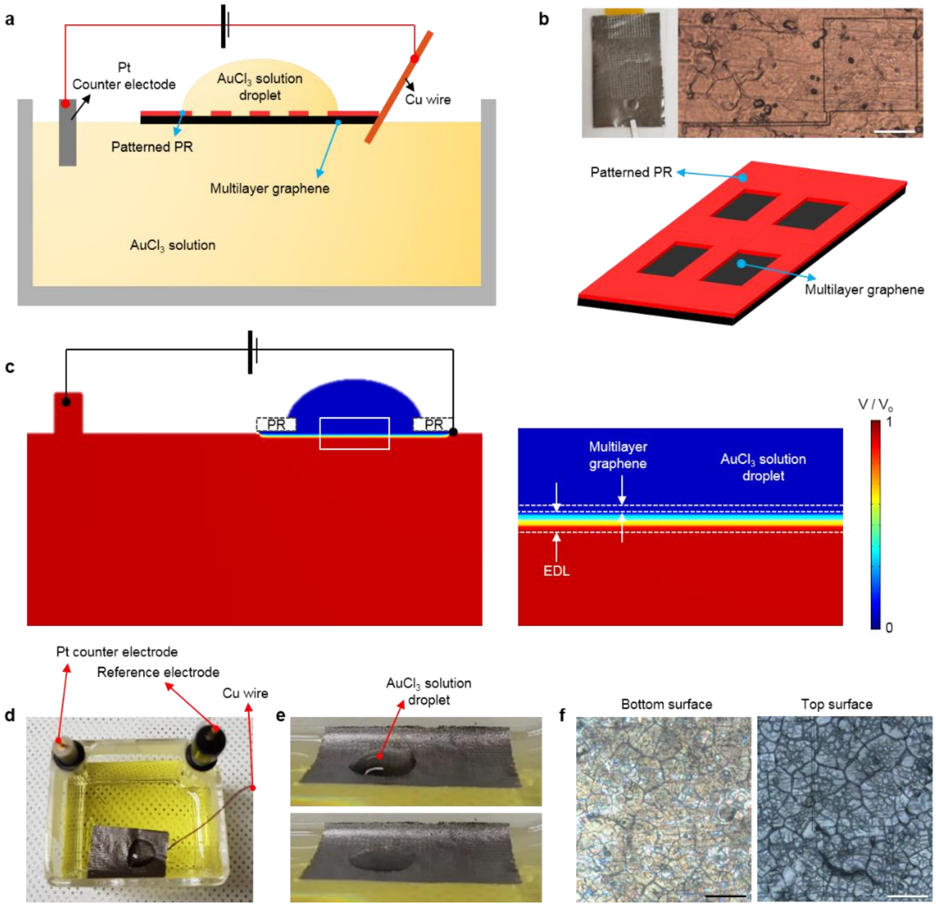

Supplementary Figure 3. Controlled experiment (II) illustrating electric potential control of the electrodeposition reaction. a, Schematic of the experimental setup of the electrochemical cells, consisting of a Pt counter electrode and multilayer graphene membrane connecting with Cu working electrode; the graphene membrane is physically supported by a patterned photo-resist (PR) layer; electrodes are exposed to a 1.0 M $AuCl_3$ aqueous electrolyte, and a droplet of the same electrolyte is placed on the graphene membrane. An external load drives the Au-deposition reaction on the graphene membrane surface. **b,** Photographs (top) and schematic of graphene membrane. Scale bar, 2μm. **c,** FEA simulation of potential distribution across the cell. Bottom panel: enlarged image taken from the square in the left panel showing that the potential gradient is concentrated on the bottom surface of the graphene membrane. **d,** Photograph of the controlled experiment with multilayer graphene on which a droplet of $AuCl_3$ aqueous electrolyte was placed. **e,** Photograph of the $AuCl_3$ droplet placed on the multilayer graphene, taken at an early stage (upper panel) and after 70 s electrodeposition (bottom panel). The $AuCl_3$ droplet gradually percolated down through the multilayer graphene sheet and became smaller, illustrating the permeation of ions/molecules to some extend through the multilayer graphene PS (See also Supplementary Movie 2). **f,** Optical-microscope images of bottom (left) and top (right) surfaces of the graphene membrane after Au-electrodeposition with an external bias of –1.8 V vs. SHE for 100 s, showing that Au-deposition occurred only on the bottom surface of the graphene membrane. Scale bars, 2μm. Despite the permeation of ions/molecules (albeit extremely slow with regards to the ionic conduction in the electrolytes), the Au electrodeposition reaction occurred only on the bottom surface of the multilayer graphene. This indicates that the $AuCl_3$ droplet is electrically separated from the $AuCl_3$ solution by the multilayer graphene. This control experiment also demonstrates the validity of our approach.

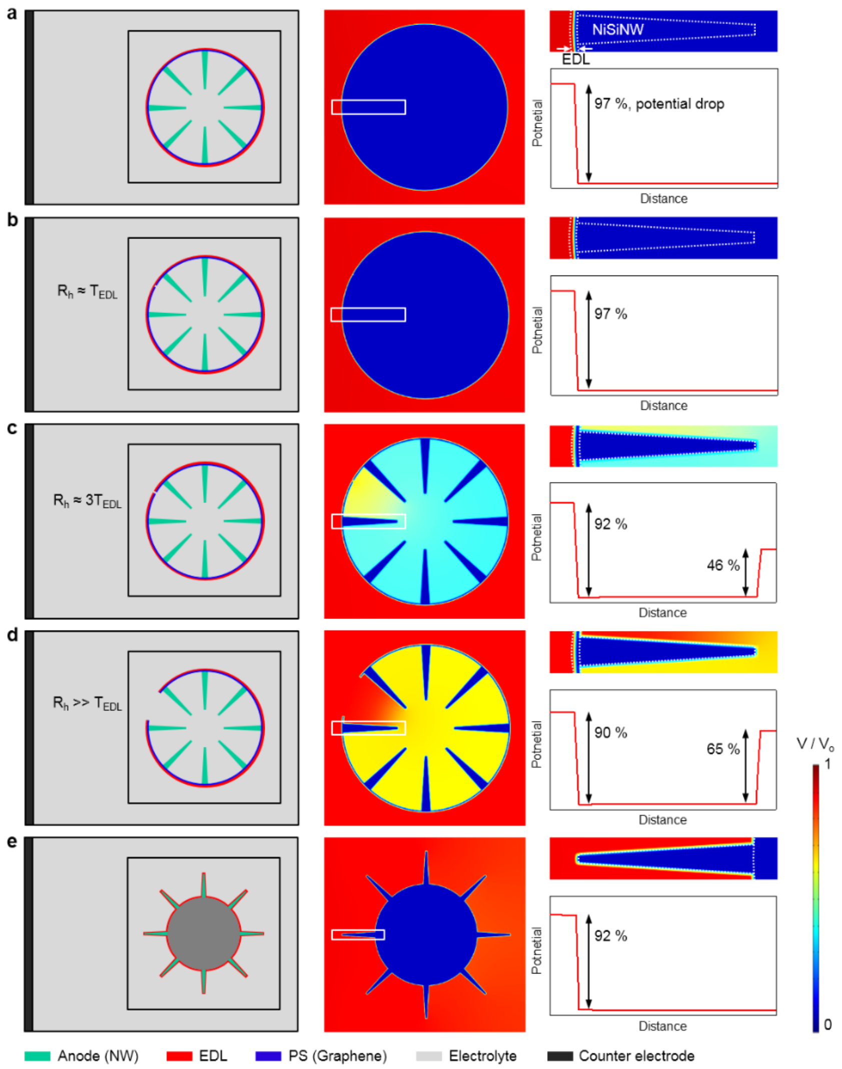

Supplementary Figure 4. FEA simulation of potential distribution. a, Schematic (left) and simulation results (middle) of the potential distributions across the NWs@GrμTs. For this simulation, we assumed that an SEI layer (15 nm in thickness) developed on the outer surface of the defect-free GrμT PS and that a thin EDL dielectric layer (5 nm in thickness) developed at the NWs/electrolyte and GrμT/electrolyte interfaces. In this simulation, an external potential bias of V_o is applied between the left-hand side electrolyte and GrμT, and the following material parameters were used: NiSiNWs (electronic conductivity of 1×10^4 S m^{-1}, length of 3 μm, and dielectric constant of 11.7), electrolyte (ionic conductivity of 820 S m^{-1}), and GrμT (electronic conductivity of 1×10^3 S m^{-1}). To visualize EDL, the thickness and dielectric constant were set to 50 nm and 3500, respectively, which produced a potential drop equivalent to that of 0.5 nm thick EDL with a dielectric constant of 35[2]. We calculated the position-dependent potential across the electrolyte and NiSiNWs@GrμT anode with respect to PS whose potential is set at ground (i.e. 0 V). Finally, the calculated potential values were normalized by the V_o. **b–d,** The change in potential distribution across the NWs@GrμTs with the existence of nanoscale and macroscopic holes in the GrμT PS, which are characterized by the correlation between the radii of holes (R_h) and EDL thickness (T_{EDL}): **b**, $R_h \approx T_{EDL}$; **c**, $R_h \approx 3T_{EDL}$; **d**, $R_h >> T_{EDL}$. **e,** The potential distribution across the NiSiNWs-on-NIF. Right panels in **a–e** show the enlarged views (top) of the white squares in the middle panels and the corresponding electrical potential profiles across the NiSiNWs (bottom).

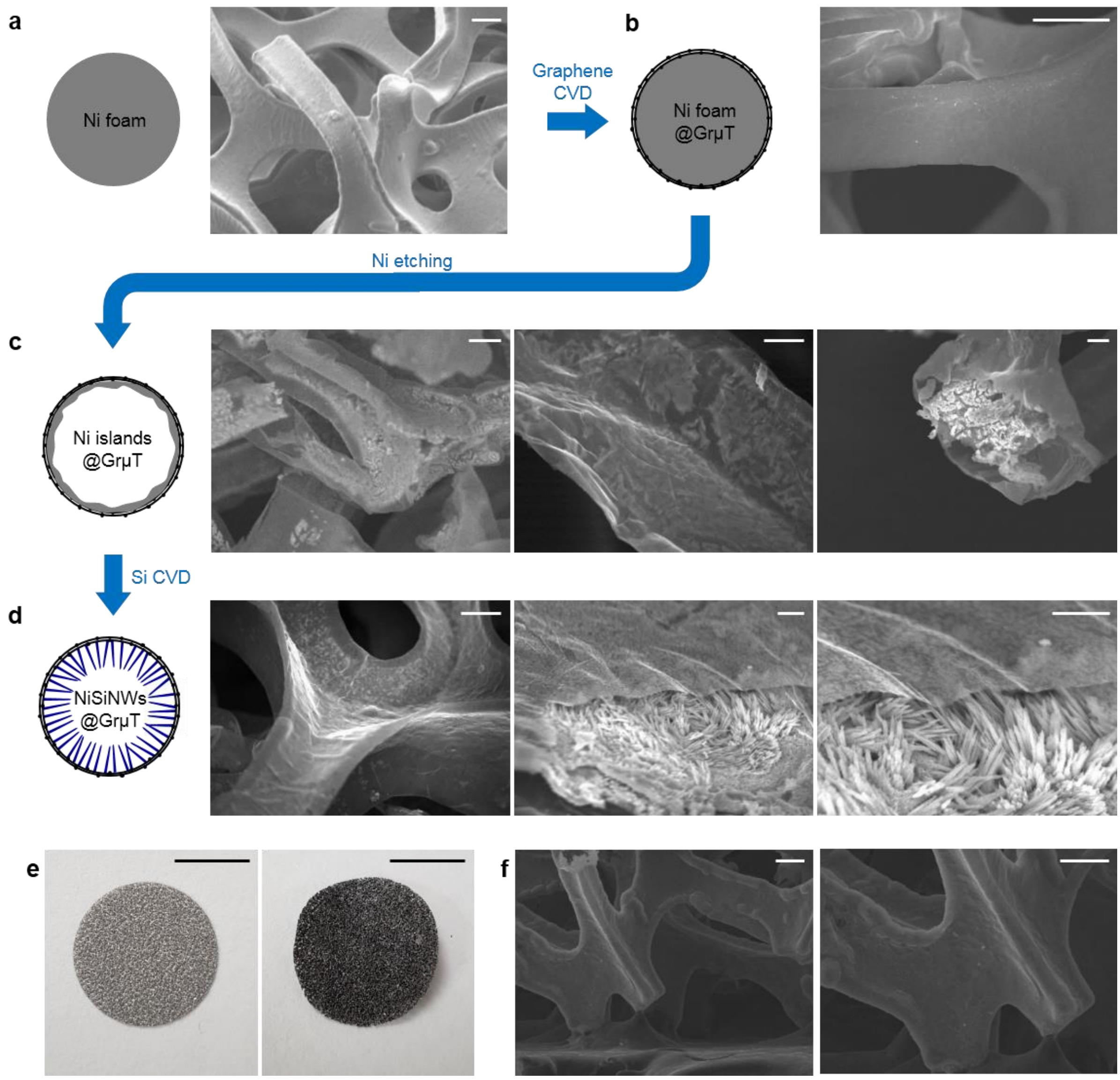

Supplementary Figure 5. Schematics and SEM images at each preparation step of NiSiNWs@GrμT. a, Ni foam. Scale bar, 50 μm. **b,** graphene-on-Ni foam. Scale bar, 50 μm. **c,** Ni islands@GrμT. Scale bars, 20 μm (left) and 10 μm (middle and right). **d,** NiSiNWs@GrμTs. Scale bars, 20 μm (left) and 2 μm (middle and right). **e,** Photographs of coin-shaped Ni foam (left panel) and NiSiNWs@GrμT electrode (right panel). Scale bars, 0.7 cm. **f,** SEM images of NiSiNWs@GrμT show that the GrμT ends remain sealed. Scale bars, 50 μm.

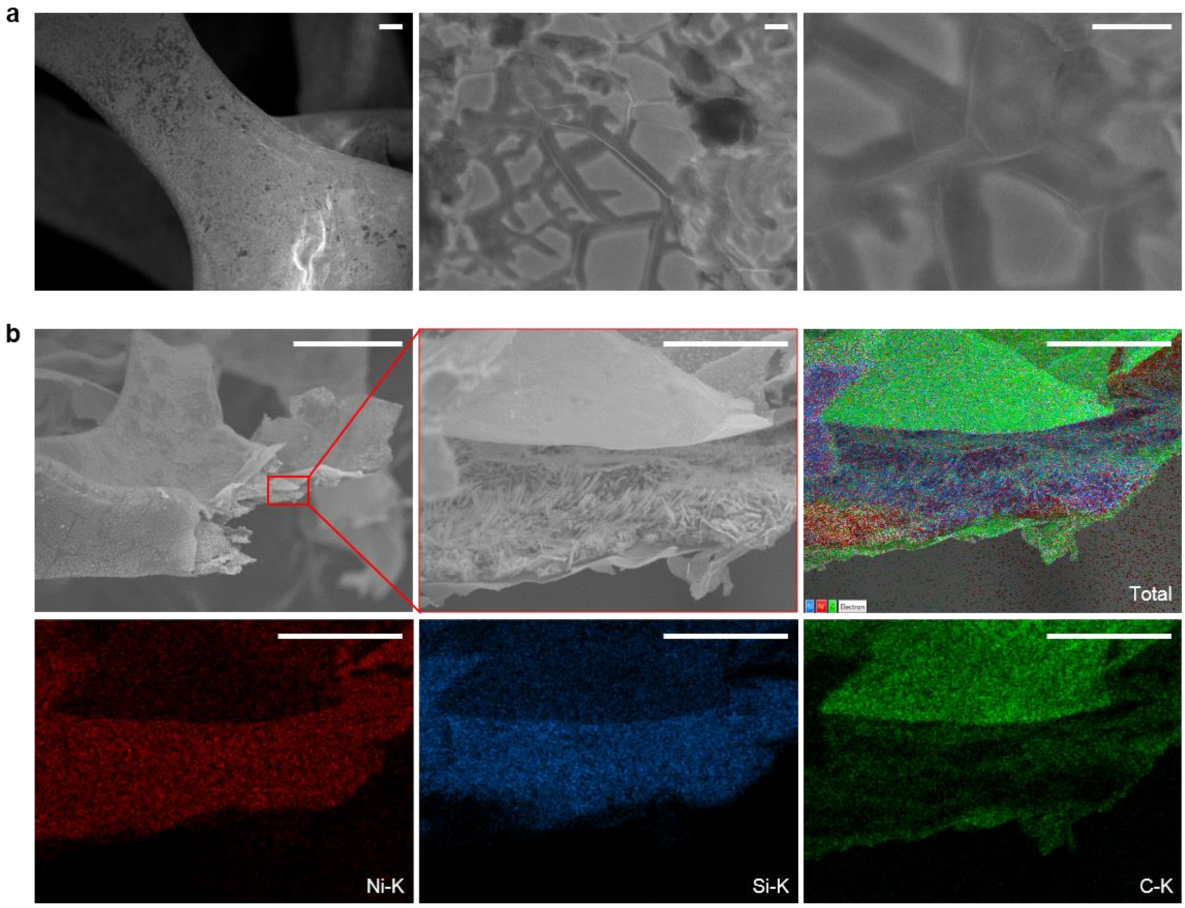

Supplementary Figure 6. SEM and EDS analyses. a, SEM images of the graphene-on-Ni foam taken during the inner Ni etching in the sulfuric acid solution. Ni etching initiated along the grain-boundaries of the GrμT. Scale bars, 10 μm (left) and 1 μm (middle and right). **b,** SEM-EDS elemental mapping images taken at the broken edges of the NiSiNWs@GrμT. Scale bars, 100 μm (upper-left) and 10 μm (all others).

a

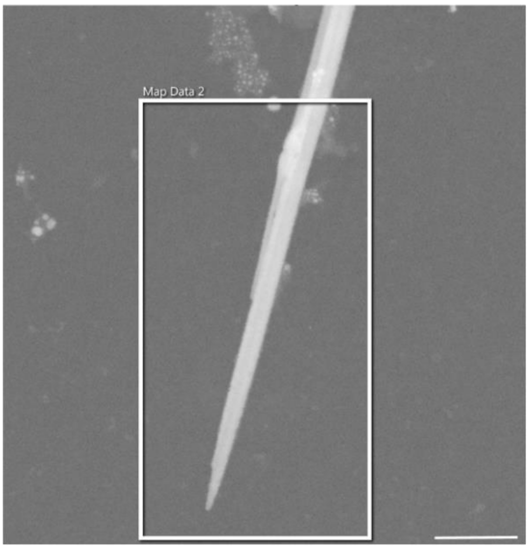

b

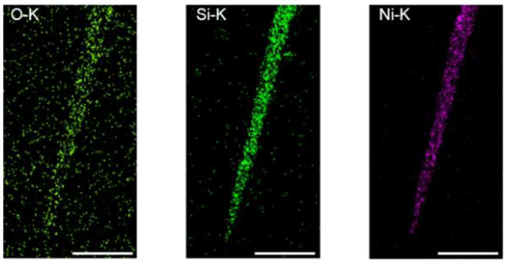

c

Element	Wt%	Atomic %
C	26.56	53.21
O	7.38	11.10
Si	19.29	**16.52**
Ni	46.77	**19.17**
Total:	100.00	100.00

Supplementary Figure 7. TEM-EDS composition analysis of NiSiNW. a, Electron microscopy image of a NiSiNW collected from an as-synthesized NiSiNWs@GrμT sample. **b,** EDS elemental mapping images of O (left), Si (middle), and Ni (right). **c,** Summary of the elemental composition in weight and atomic percent (excluding carbon). Scale bars, 250 nm.

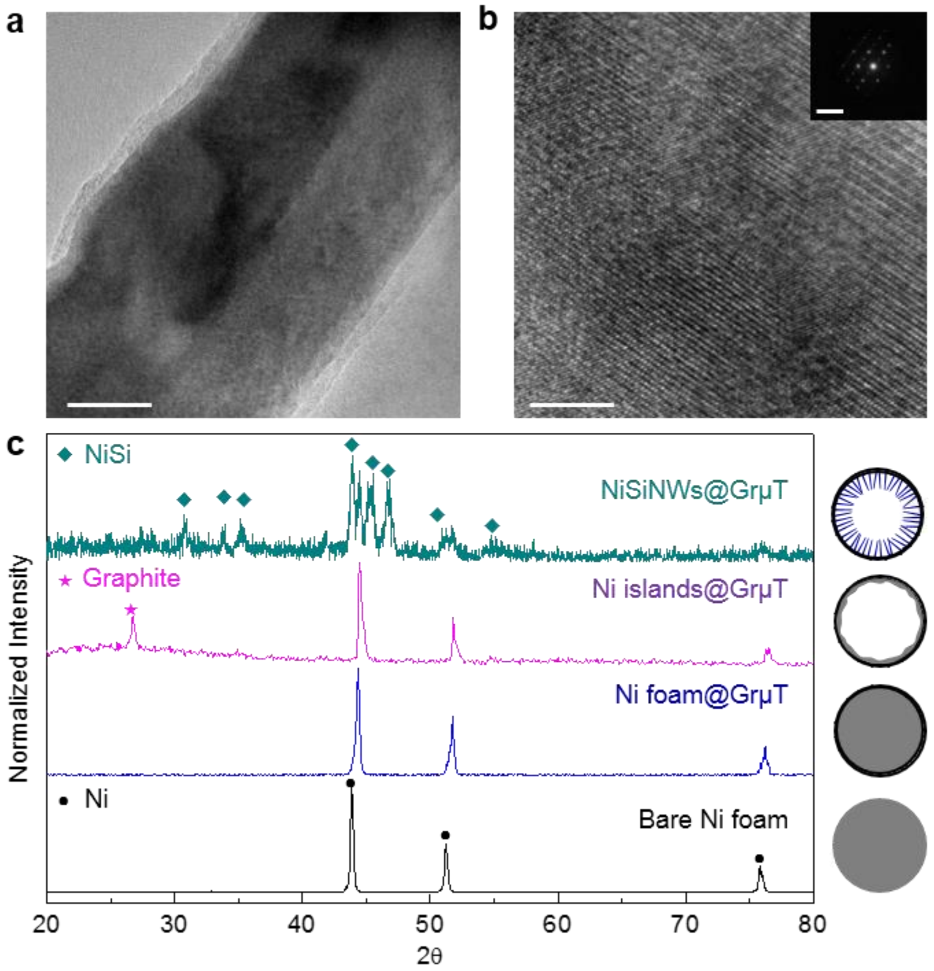

Supplementary Figure 8. Structural analyses of NiSiNWs@GrμTs. a,b, HR-TEM image of NiSiNWs collected from the as-synthesized NiSiNWs@GrμT sample. Lattice-resolved image and selective area electron diffraction pattern (inset) confirm the single-crystal structure of the NiSiNW. Scale bars, 20 nm (a) and 5 nm (b). **c,** XRD patterns of the synthetic products in each step. After the CVD process, new diffraction peaks corresponding to the NiSi phase appear, while the intensities of Ni peaks decease significantly.

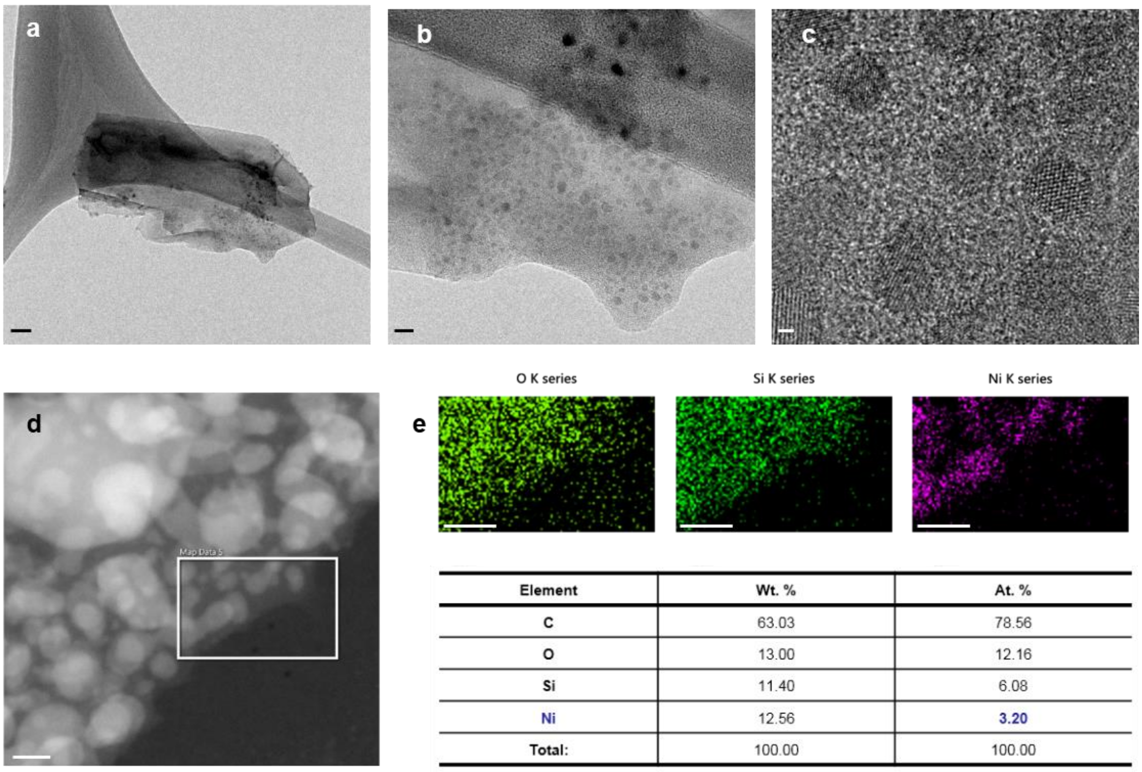

Element	Wt. %	At. %
C	63.03	78.56
O	13.00	12.16
Si	11.40	6.08
Ni	12.56	3.20
Total:	100.00	100.00

Supplementary Figure 9. TEM-EDS analyses of GrμT fragments separated from the NiSiNWs@GrμT. a-c, TEM images in low (a) and high (b,c) magnification of a GrμT fragment placed on a lacey carbon film supported by a copper grid. The fragments were prepared by sonicating the NiSiNWs@GrμT sample. During the sonication, most NiSiNWs were detached from the fragments and only GrμT flakes coated with thin island layers remained. The high magnification images show that 5-20 nm-diameter Ni nanoparticles were embedded in amorphous islands. Scale bars, 20 nm (a), 10 nm (b), and 1 nm (c). **d,e,** TEM-EDS element analysis of a GrμT flake taken over the area inside the square in this dark-field image (d). From the elemental mapping images (upper panels in e) and summaries of the elemental composition (bottom table in e), the islands are determined to be amorphous SiO_X. Scale bars, 10 nm (d, e). This analysis illustrates that during the CVD NiSiNW growth, thin Si island layers also developed on the surface of Ni islands, and at the same time Ni-residue, which is not involved in NiSiNWs growth, formed nanoparticles inside/around the Si islands that were thereafter oxidized to form amorphous SiO_x islands.

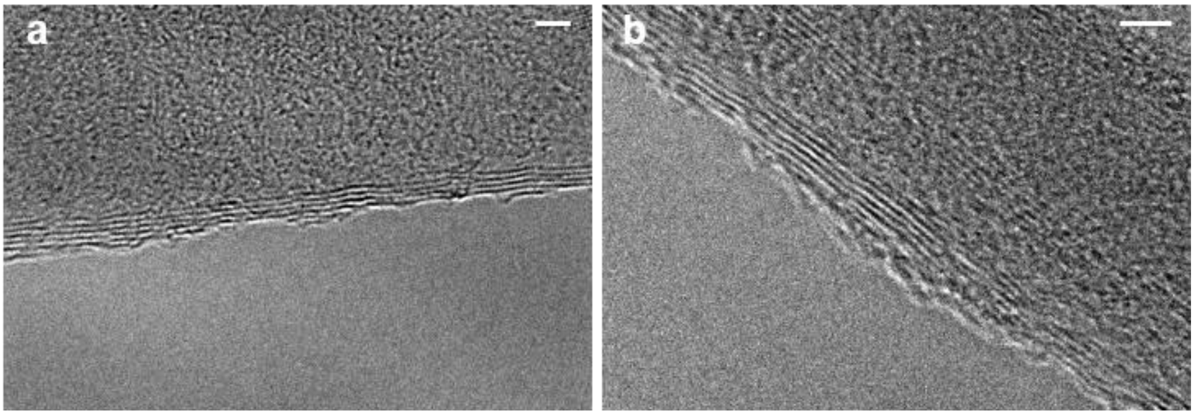

Supplementary Figure 10. TEM analyses of GrμT PS. a,b, HR-TEM images taken at the edges of GrμT walls collected from an Ni islands@GrμT sample, showing that the GrμT consists of only a few layers of graphene (five to seven layers). Scale bars, 2nm.

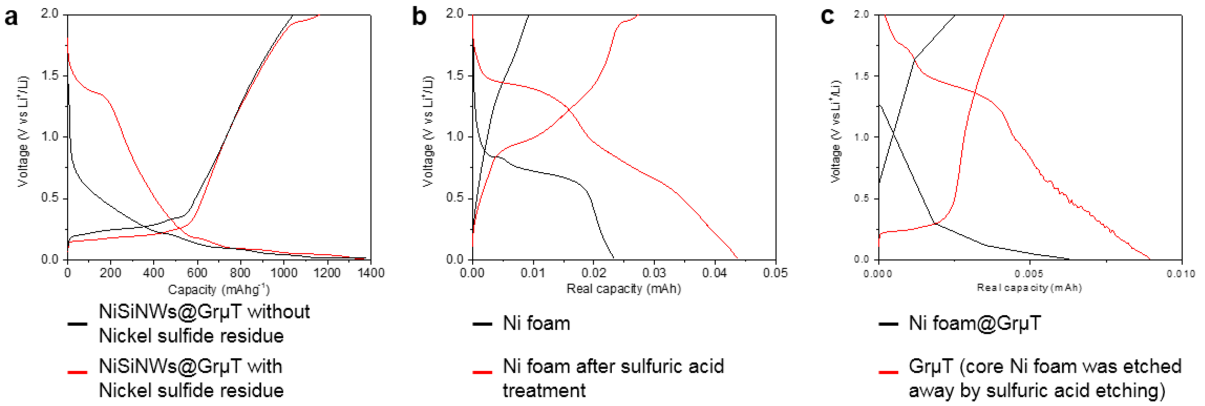

Supplementary Figure 11. Comparison of galvanostatic charge-discharge profiles for diverse samples depending on sulfuric acid treatment. a, NiSiNWs@GrμTs that underwent Ni core etching with hydrochloric acid (black line) and sulfuric acid (red line). **b,c,** Both the Ni foam (**b**) and Ni foam@GrμT (**c**) exhibited new plateaus at 1.4 V and 1.9 V, respectively, only after sulfuric acid treatment.

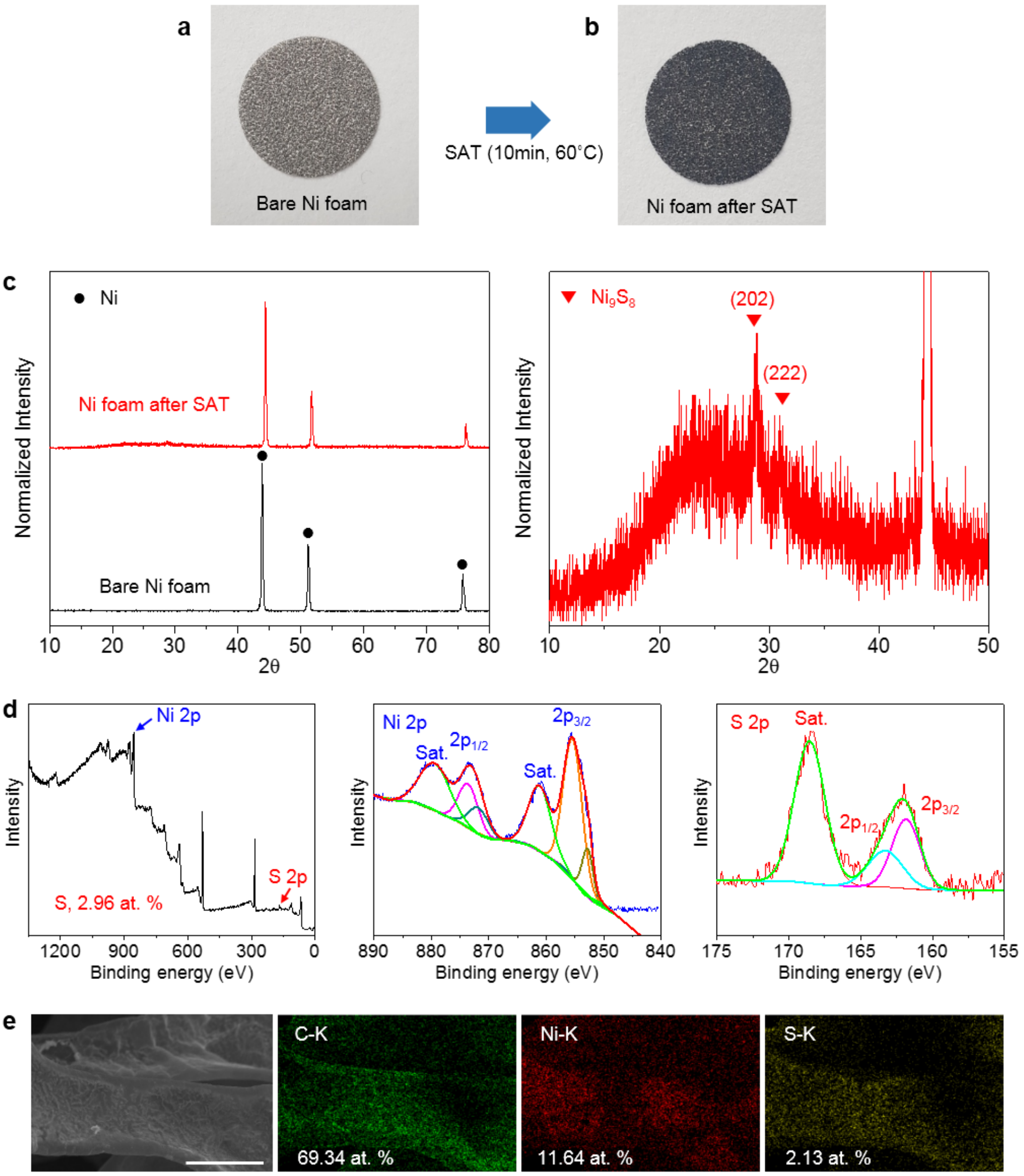

Supplementary Figure 12. Structural analyses of nickel sulfide phase on Ni foam. a,b, Photographs of bare Ni foam (**a**) and Ni foam after sulfuric acid treatment (SAT) (**b**). **c,** XRD measurements of bare Ni foam and Ni foam after SAT (left panel) and enlarged pattern of Ni foam after SAT (right panel). **d,** X-ray photoelectron spectroscopy (XPS) spectrum of Ni foam after SAT in a wide scan (left panel) and high-resolution XPS spectra of Ni 2p (middle panel) and S 2p (right panel). In Ni 2p spectrum, 853.6 and 871.18 eV are assigned to $2p_{3/2}$ and $2p_{1/2}$ of Ni^{2+}, and the peaks at 855.3 and 873.3 eV are assigned to $2p_{3/2}$ and $2p_{1/2}$ of Ni^{3+}, respectively. In S 2p spectrum, 161.3 and 163.4 eV are assigned to S $2p_{3/2}$ and S $2p_{1/2}$ of Ni–S bonding[3], respectively. **e,** SEM (left) and EDS element mapping images of Ni islands@GrμT. Scale bars, 25 μm.

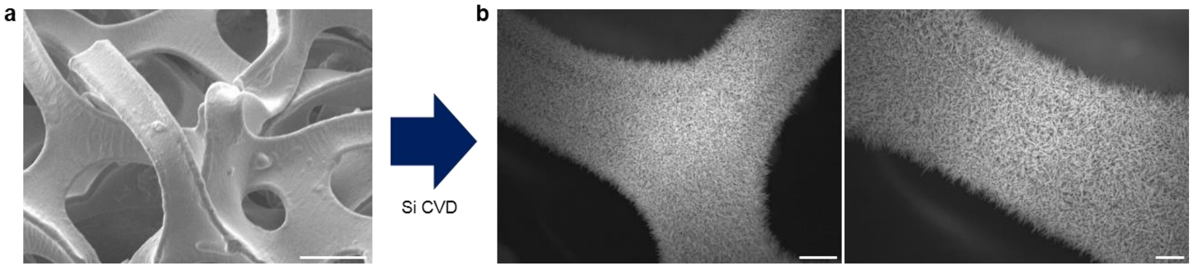

Supplementary Figure 13. Preparation of NiSiNWs-on-NiF. a, SEM image of bare Ni foam. Scale bar, 100 μm. **b,** SEM images of NiSiNWs-on-NiF. The NiSiNWs are grown on the surface of Ni foam in the CVD reactor. For NiSiNW growth, a gas mixture of 10% SiH_4 in H_2 was introduced at a flow rate of 50 sccm, and the reactor pressure and temperature were kept at 20 torr and 460 ˚C, respectively, for 20 min. Scale bars, 10 μm.

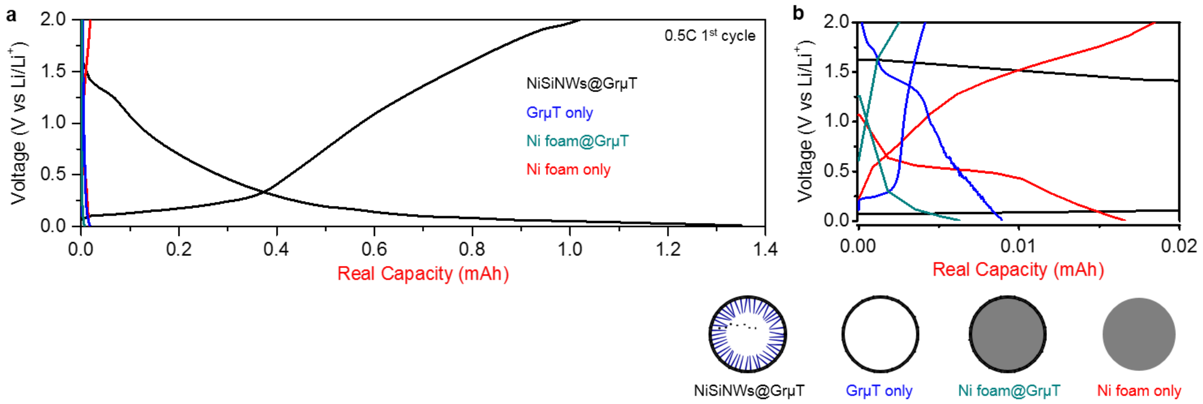

Supplementary Figure 14. Comparison of real capacity. a, GCD profiles of four different kinds of anodes consisting of NiSiNWs@GrμT (black), bare GrμT (blue), Ni foam@GrμT (green), and bare Ni foam (red); these illustrate that the contribution of the GrμT and Ni on the total capacity is negligible. **b,** The identical GCD profiles highlighting the real capacity (x-axis) in the range of 0.00–0.02 mAh, showing that the real capacities of the GrμT and Ni foam are smaller than 0.02 mAh, which is less than 2% of the total capacity of the NiSiNWs@GrμT.

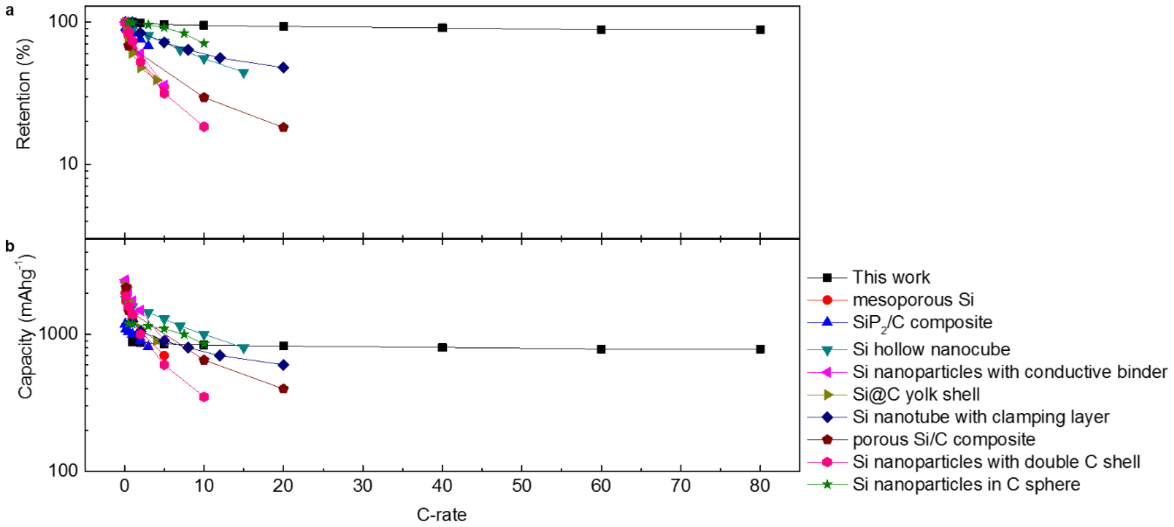

Supplementary Figure 15. Comparison of the rate capability with state-of-the-art Si-based anode materials. a, A plot of specific capacities as a function of the discharge rate for the NiSiNWs@GrμTs anode and other LIB anodes in the literature[4–12]. **b,** A plot of capacity retentions as a function of the discharge rate for the NiSiNWs@GrμTs anode and LIB anodes in the literature. To date, the state-of-the-art Si-based anodes have the highest capacity values in the range of 600 $mAhg^{-1}$ at a cycling rate of 12C; however, most of these undergo fast capacity fading with increased cycling speed, showing capacity retention of less than 50% at 12C (vs. the capacity at 1C).

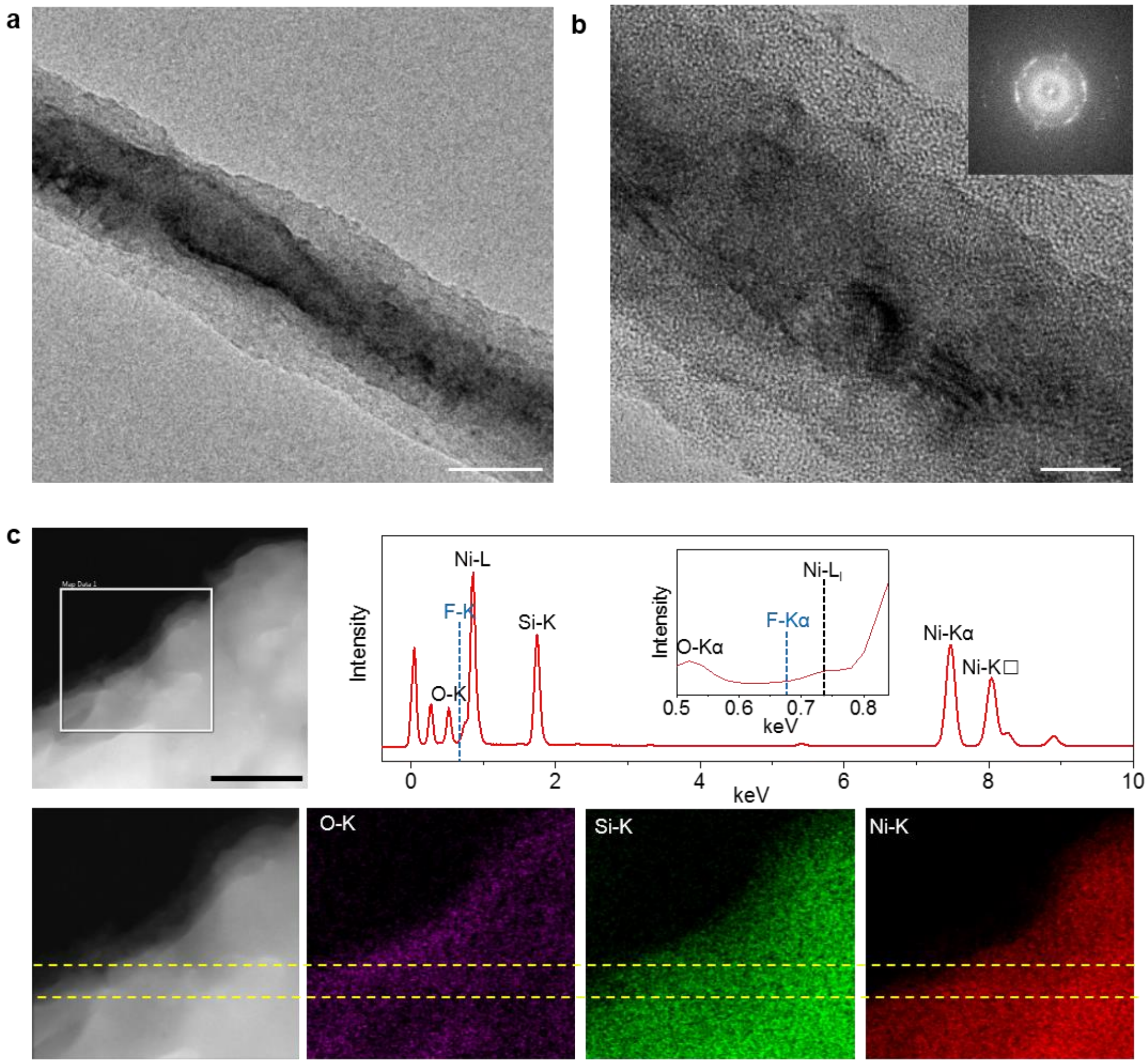

d

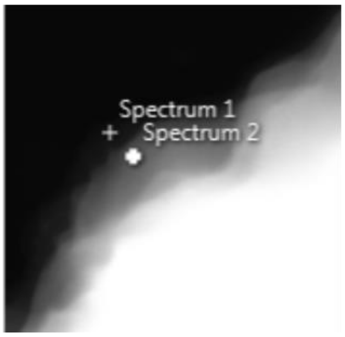

Spectrum 1

Element	Weight %	Atomic %
O	9.98	22.06
Si	32.87	41.40
S	0.06	0.06
Ni	55.42	33.39
Total:	100	100

Spectrum 2

Element	Weight %	Atomic %
O	29.70	43.50
Si	62.93	52.51
S	0.00	0.00
Ni	5.66	2.26
Total:	100	100

Supplementary Figure 16. TEM analysis of delithiated NiSiNW. a,b, HR-TEM image of NiSiNW collected from delithiated NiSiNWs@GrμT after 500 cycles at 10C. Lattice-resolved image and selective area electron diffraction pattern (inset in b) confirm the poly-crystal structure of the NiSiNW. Scale bars, 50 nm (a) and 10 nm (b). **c**, EDS element analysis of delithiated NiSiNW, taken along the area in the upper-left panel of the dark-field image of the NiSiNW. Upper-right panel: EDS spectrum and elemental mapping images (bottom panels) show that outer shell of the NiSiNW consists mainly of SiOx without SEI; the P and F associated with the SEI layer component are not detected within the detection limit. Scale bar. 25 nm. **d,** Summaries of elemental compositions at the crossbar points.

a

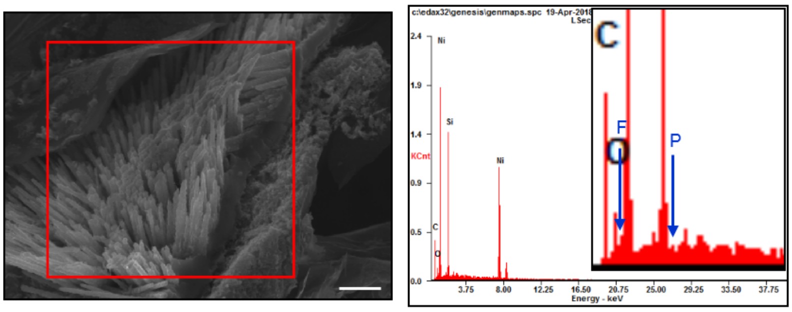

Element	Wt%	At%
CK	32.52	63.81
OK	3.08	4.53
FK	0.74	0.92
SiK	11.81	9.91
PK	0.06	0.04
NiK	51.80	20.79
Matrix	Correction	ZAF

b

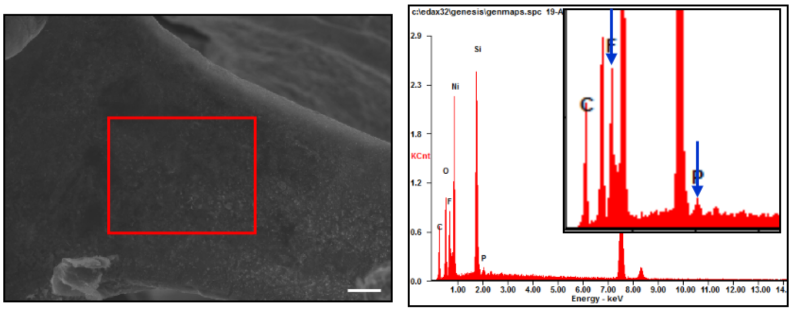

Element	Wt%	At%
CK	26.27	46.79
OK	14.84	19.84
FK	7.94	8.94
SiK	14.37	10.95
PK	0.47	0.32
NiK	36.11	13.16
Matrix	Correction	ZAF

c

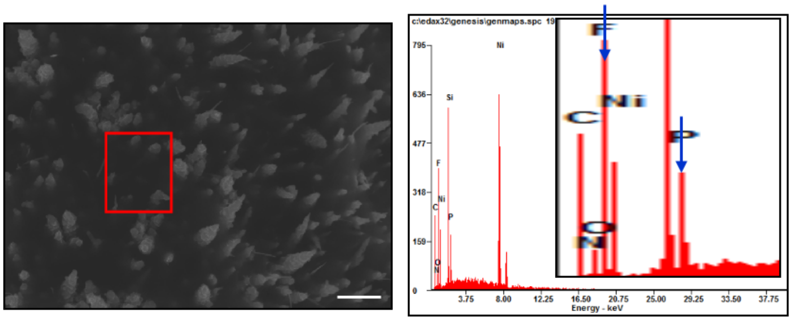

Element	Wt%	At%
CK	28.95	56.88
NK	2.52	4.25
OK	1.82	2.69
FK	6.36	7.90
SiK	7.04	5.91
PK	2.60	1.98
NiK	50.71	20.38
Matrix	Correction	ZAF

Supplementary Figure 17. SEM image and EDS analysis. a-c, SEM images (left), EDS spectra (middle) and summaries of the elemental composition (right) of the delithiated NiSiNWs-on-NiF (**a**) and delithiated NiSiNW@GrμT (**b,c**). In **c**, GrμT was opened to analyze the NiSiNWs inside. The blue arrows in the insets of the middle panels indicate the positions of F K-series (left) and P K-series (right) transitions. Scale bars, 2 μm (a and c) and 10 μm (b).

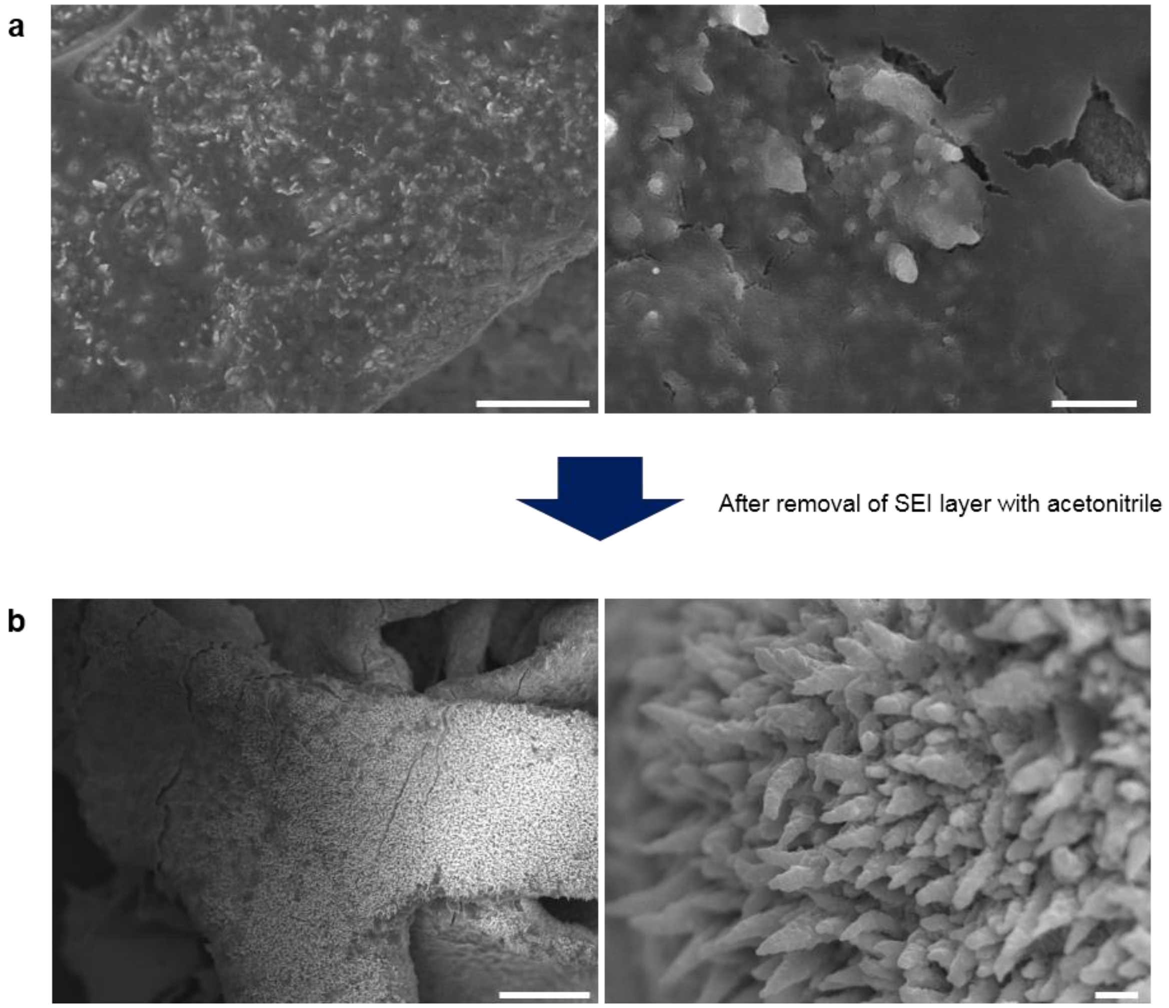

Supplementary Figure 18. Morphology and SEI formation of NiSiNWs-on-NiF after cycling. a, SEM images of NiSiNWs-on-NiF after 100 cycles. After dissembling the coin cell, the NiSiNWs-on-NiF electrode was rinsed with an EC/DEC solvent without $LiPF_6$ salt and dried in a glove box. As can be seen, NiSiNWs were embedded into the thick SEI layer. Scale bars, 10 μm (left) and 1 μm (right). **b,** SEM images of NiSiNWs-on-NiF rinsed with acetonitrile to remove the SEI layer and to identify the morphology of NiSiNWs. Scale bars, 30 μm (left) and 1 μm (right).

Supplementary References

1. Bisquert, J., Cendula, P., Bertoluzzi, L. & Gimenez, S. Energy Diagram of Semiconductor/Electrolyte Junctions. *J. Phys. Chem. Lett.* **5**, 205–207 (2014).
2. Hall, D. S., Self, J. & Dahn, J. R. Dielectric Constants for Quantum Chemistry and Li-Ion Batteries: Solvent Blends of Ethylene Carbonate and Ethyl Methyl Carbonate. *J. Phys. Chem. C* **119**, 22322–22330 (2015).
3. An, T. C. *et al.* Interlaced NiS2-MoS2 nanoflake-nanowires as efficient hydrogen evolution electrocatalysts in basic solutions. *J. Mater. Chem. A* **4**, 13439–13443 (2016).
4. Chen, S. Q., Shen, L. F., van Aken, P. A., Maier, J. & Yu, Y. Dual-Functionalized Double Carbon Shells Coated Silicon Nanoparticles for High Performance Lithium-Ion Batteries. *Adv. Mater.* **29**, 1605650 (2017).
5. Jeong, M. G. *et al.* Self-Rearrangement of Silicon Nanoparticles Embedded in Micro Carbon Sphere Framework for High-Energy and Long-Life Lithium Ion Batteries. *Nano Lett.* **17**, 5600–5606 (2017).
6. Kwon, H. T., Lee, C. K., Jeon, K. J. & Park, C. M. Silicon Diphosphide: A Si-Based Three-Dimensional Crystalline Framework as a High Performance Li-Ion Battery Anode. *ACS Nano* **10**, 5701–5709 (2016).
7. Lawes, S. *et al.* Inkjet-printed silicon as high performance anodes for Li-ion batteries. *Nano Energy* **36**, 313–321 (2017).
8. Liang, J. *et al.* A Deep Reduction and Partial Oxidation Strategy for Fabrication of Mesoporous Si Anode for Lithium Ion Batteries. *ACS Nano* **10**, 2295–2304 (2016).
9. Lin, D. C. *et al.* A high tap density secondary silicon particle anode fabricated by scalable mechanical pressing for lithium-ion batteries. *Energ. Environ. Sci.* **8**, 2371–2376 (2015).
10. Liu, J., Kopold, P., van Aken, P. A., Maier, J. & Yu, Y. Energy Storage Materials from Nature through Nanotechnology: A Sustainable Route from Reed Plants to a Silicon Anode for Lithium-Ion Batteries. *Angew. Chem. Int. Ed.* **54**, 9632–9636 (2015).
11. Wu, H. *et al.* Stable cycling of double-walled silicon nanotube battery anodes through solid-electrolyte interphase control. *Nat. Nanotechnol.* **7**, 309–314 (2012).
12. Yoon, T. *et al.* Mesoporous Silicon Hollow Nanocubes Derived from Metal-Organic Framework Template for Advanced Lithium-Ion Battery Anode. *Acs Nano* **11**, 4808–4815 (2017).
