# Supplementary material for: Controlling electric potential to inhibit solid-electrolyte interphase formation on nanowire anodes for ultrafast lithium-ion batteries
Source: Nat Commun. 2018 Aug 27;9:3461. doi: 10.1038/s41467-018-05986-9 (PMC6110779; doi:10.1038/s41467-018-05986-9)
Supplement: Supplementary file 3 — Description of Additional Supplementary Files [file 41467_2018_5986_MOESM3_ESM.docx]

**Description of Additional Supplementary File**

File Name: Supplementary Movie 1

Description: Hydrogen evolution reaction of CuO/Cu bowl in Controlled Experiment (I)

File Name: Supplementary Movie 2

Description: Permeation of AuCl3 droplets through multilayer graphene in Controlled Experiment (II)
