# Supplementary material for: Controlling electric potential to inhibit solid-electrolyte interphase formation on nanowire anodes for ultrafast lithium-ion batteries
Source: Nat Commun. 2018 Aug 27;9:3461. doi: 10.1038/s41467-018-05986-9 (PMC6110779; doi:10.1038/s41467-018-05986-9)
Supplement: Supplementary file 2 — Peer Revew File [file 41467_2018_5986_MOESM2_ESM.pdf]

Editorial Note: Parts of this peer review file have been redacted as indicated to remove third-party material where no permission to publish could be obtained.

Reviewers' comments:

Reviewer #1 (Remarks to the Author):

The authors proposed a new strategy for inhibiting SEI formation on the anode surface through engineering the electric potential by using an atomically-thin and ion-permeable PS. While it is an interesting design, the data could not support the assumption. Therefore, this manuscript can't be published on Nature Communications at least in the current form. A few issues are listed below:
1. The controlled experiment in supplementary figure 1, the authors used a copper (Cu) bowl coated with a thermally oxidized layer (CuO) on both the outer and inner surfaces, so the KOH inside the bowl had no contact with KOH outside the bowl, it is different in the GrµT structure. In the GrµT structure, the electrolyte inside the PS indeed contacted with the electrolyte outside.
2. The authors need to add some SEM and TEM characterizations about the graphene ps before and after etching.
3. Why exist a plateau at 1.4 V in the galvanostatic charge-discharge (GCD) profiles? I think it's not SEI formation because the electrolyte used (1 M LiPF6 in EC/EDC) always decomposes below 1V.
4. One hypothesis is that Ni may not be completely removed in the process of Ni etching. After growth of NiSi nanowries, still some Ni existed inside the graphene, so it can enhance the electronic conductivity. Maybe that's the important cause for such a good rate performance of this structure. I think it need some experimental data to support the author's result (ie. SEM eds mapping, TEM eds mapping including the graphene part).
5. Too many mistakes in the manuscript. For example, Page 6 line 22, "while the diameter and length of NiSiNWs are ~8-10 µm and 80-150 nm, respectively."; the order of a b c d in the figure 4 caption is totally confused.

Reviewer #2 (Remarks to the Author):

This manuscript describes a new method designed to control the SEI formation in Si-based nanowires used as anodes for Li ion batteries. The designed is explained as a way to control the potential to which the nanowires are exposed in contact with the electrolyte solution. However there are several points that are not clear and should be addressed before the article can be reconsidered for publication.
1. In the new design the Li ions should go through an "atomically thin" wall named "potential sheath" (PS). This PS is made of graphene. Li ions cannot go through the aromatic rings unless there are defects. How can the ions go through to a perfect graphene layer?
2. Page 5. It is suggested that the potential inside the space where the nanowire interfaces with the electrolyte remains lower than VA, and therefore the SEI does not form. It is not clear why. How does the potential of the nanowire, NiSi, varies upon lithiation? The authors should provide information about this lithiation and the voltage of Li intercalation in NiSi. That would clarify what is the potential. Probably all the results can be explained by the use of this alternative material, NiSi.
3. I don't understand how FEA simulations "can predict" voltages. They will only reproduce voltages

given as input. Please explain. If the profile shown in Figure 1d (right) were correct, the nanowires wouldn’t be able to be lithiated.

4. The structure of the nanowires (presence of porous Ni) can explain the smooth behavior during lithiation. The authors should present further characterization of the structure of the nanowires and lithiation behavior. Some publications (JES 154, A97-A102) have suggested that during lithiation NiSi undergoes reversible transformation to/from Ni2Si and LiySi. This transformation has a change of volume opposite to lithiation which may stabilize the system and make the SEI more stable due to the absence of a significant volume change. But it is hard to believe that a SEI is not formed over the nanowire. The authors should review their explanation once these details are clarified.

Reviewer #3 (Remarks to the Author):

This manuscript reports the fabrication of silicide nanowires (NWs) anchored selectively to the inner surface of micro-tubular graphene electrodes through the selective etching followed by CVD process. The authors further claimed that this structure design could electrically shields the electrolyte from an external potential load, eliminating the driving force that generates the SEI on the NW surface during lithiation. Although we agree with the novelties in terms of material design and good cycle performance reported in this manuscript. However, the established "potential shielding" model are not convincing simply based on the electrolytic water experiment shown in the supporting information. Additionally, the characterization revealed some noticeable issues yet to be revised.

Firstly, the so-called "potential shielding" effect for the NiSi NWs in the electrolyte is not comparable to the electrolytic water experiment (supporting information) in which the solution inside of the copper crucible do not form a complete loop with external electric load. Since this part of solution does not necessarily participate in the reaction, FEA simulation results for the potential gradient develops formed on the surface of the graphene PS provides limited comparative meaning.

Secondly, the characterizations results cannot confirm that SEI layer develops only on the outer surface of the Grμ T-based PS, thus the statement of the SEI-free NiSiNWs after cycling is problematic. In the figure 4c, the post-mortem NiSiNWs@GrμTs demonstrate a amorphous layer on the NW surface which seems to be SEI layer. Although the authors claim this is the amorphous oxide layer formed on the SiNi NW, the elemental EDS mapping is needed.

Finally, What does the oxidation peak at around 1.9V and the reduction peak at ~ 1.3V represents in Figure 3a (CV curve)? A detailed explanation of reaction mechanism is needed.

Reviewer #1 (Remarks to the Author):

The authors proposed a new strategy for inhibiting SEI formation on the anode surface through engineering the electric potential by using an atomically-thin and ion-permeable PS. While it is an interesting design, the data could not support the assumption. Therefore, this manuscript can't be published on Nature Communications at least in the current form. A few issues are listed below:

Author Response: We thank the reviewer for the thoughtful comments about our manuscript. We welcome the opportunity to address and clarify the issues raised in the reviewer's report, and believe that the additional experiments and revisions substantially strengthen our manuscript. Our responses to the points raised in the report are below.

Comment (1): The controlled experiment in supplementary figure 1, the authors used a copper (Cu) bowl coated with a thermally oxidized layer (CuO) on both the outer and inner surfaces, so the KOH inside the bowl had no contact with KOH outside the bowl, it is different in the GrμT structure. In the GrμT structure, the electrolyte inside the PS indeed contacted with the electrolyte outside.

Author Response: We agree with the reviewer's comment that a CuO/Cu bowl and a GrμT have a different characteristic as potential sheath (PS) in that the CuO/Cu bowl is not ion/solution permeable. However, since the GrμT ends are sealed, the electrolytes inside and outside of the GrμT do not directly contact each other (although ion/solution are permeable in the GrμT). In this situation, the transport rate of Li through the GrμT is much lower than that of the electron transport in the GrμT so that the potential drop in the GrμT structure was determined by electron conduction (i.e., potential becomes nearly equivalent across the GrμT due to good electron conduction). In addition, since the EDL that develops in the electrolyte near the GrμT surface hinders ion/electron conduction, most of the potential gradient is created in the EDL. It is also noteworthy that even nanoscale cracks exist in the PS, the penetration of electric potential into inside of the GrμT could be effectively suppressed so that the potential gradient inside would not be enough to initiate the SEI formation (see Supplementary Fig. S3b, c in our revised Supplementary Information).

In view of these considerations, we can conclude that both the GrμT and the CuO/Cu bowl electrodes produce the similar potential profile across the outer electrolyte/electrode/inner electrolyte (see Fig. R1 and Supplementary Fig. S1b,c in our revised Supplementary Information). Accordingly, given that most electrochemical reactions (e.g., SEI formation, lithiation, hydrogen evolution, etc.) are governed by a potential gradient, the CuO/Cu bowl experiment could be useful to explain the mechanism of our approach exploiting electric potential control to prevent SEI formation on the NW surface.

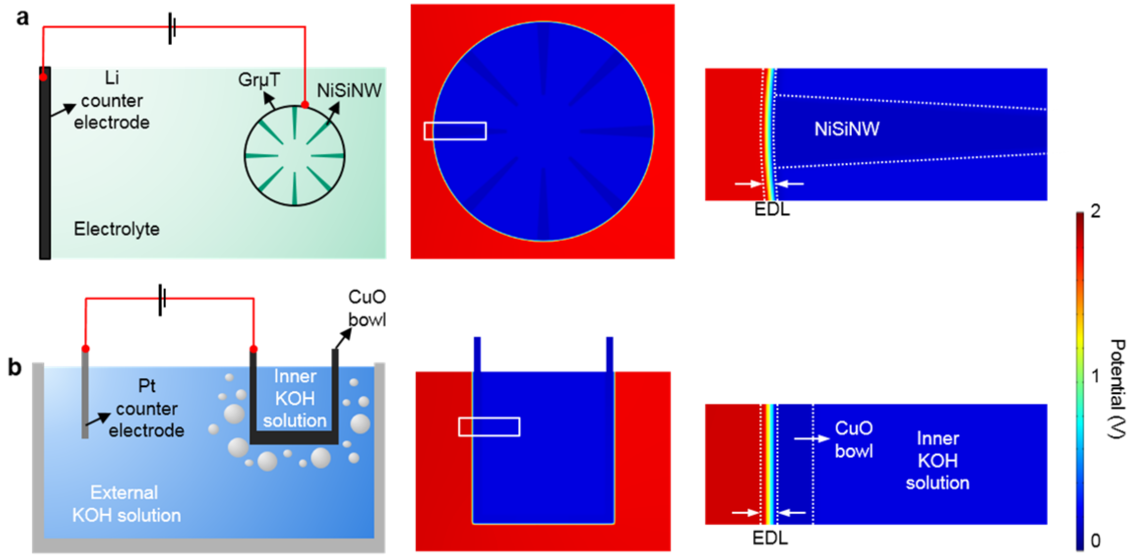

Figure R1. Similarity of potential profile across the outer electrolyte/electrode/inner electrolyte between the NiSiNWs@GrμT (**a**) and CuO/Cu (**b**) bowl anodes of the electrochemical cells.

However, to address the concern that reviewer raised, we have conducted another controlled experiment (II) using multilayer graphene PS, instead of CuO/Cu bowl (see new Supplementary Fig. S2 in our revised Supplementary Information). (Please understand that we had to carry out the electrodeposition experiment instead of lithiation experiment considering practical limits such as oxidation of lithium metal & electrolyte and safety).

The multilayer graphene membrane that was supported by patterned photoresist layer was transferred onto a 1.0 M $AuCl_3$ aqueous electrolyte and a droplet of same electrolyte was placed on the graphene membrane to form additional interface on the top surface of the graphene. As shown in Supplementary Fig. S2a, an external load was applied, between a Pt counter electrode and a Cu wire connected to one end of the graphene membrane, to drive the Au electrodeposition reaction at the electrolyte and graphene interface. After the electrodeposition with an external bias of -1.8 V vs V_{SHE} for 100 s, we found that Au-deposition occurred only on the bottom surface of the graphene membrane where the potential gradient was created. This control experiment also demonstrates the validity of our approach (i.e., the SEI formation and Li insertion occur only on the external electrolyte/GrμT interface where the potential gradient is created).

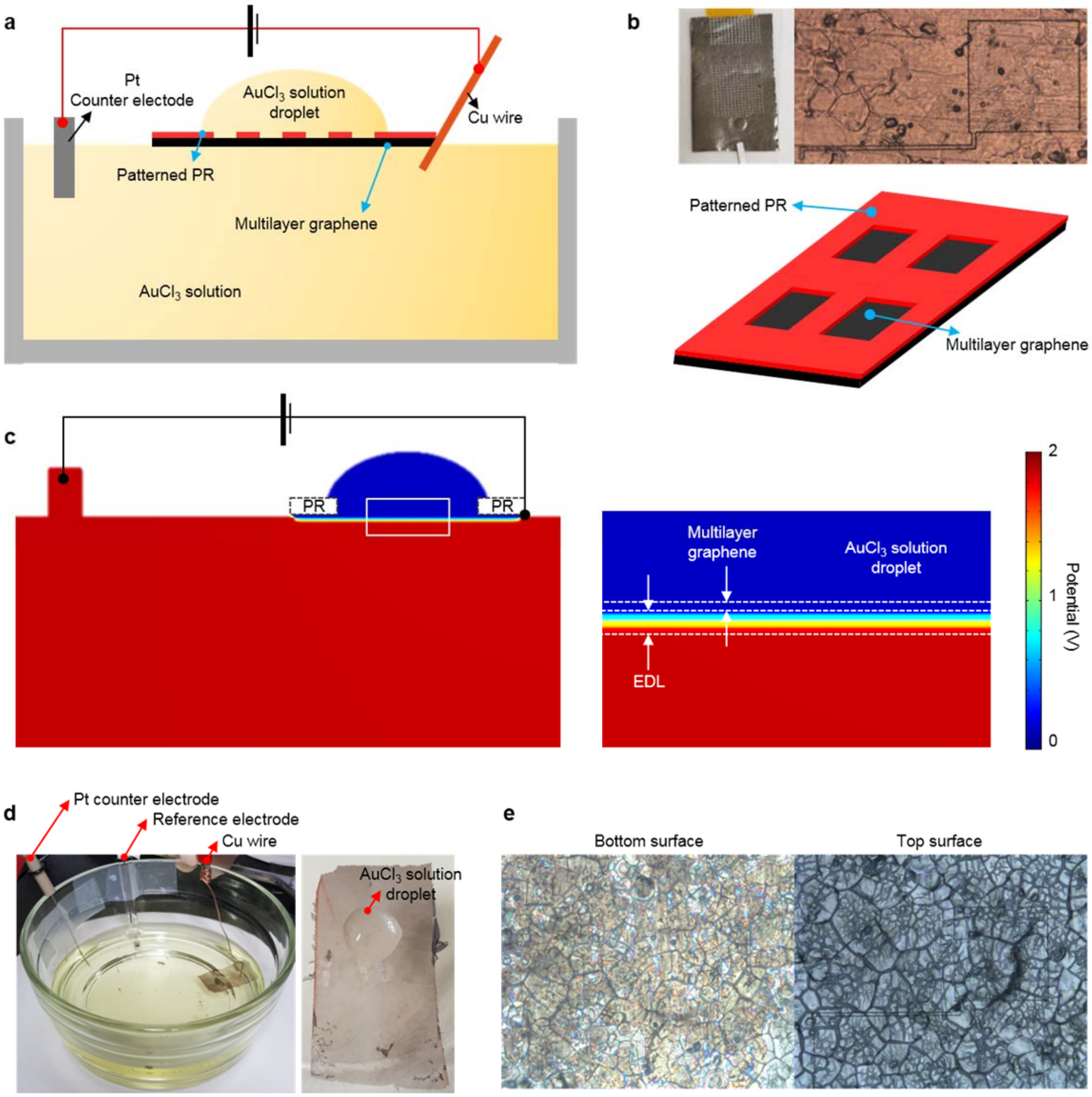

Supplementary Figure S2. Controlled experiment (II) illustrating electric potential control of the electrodeposition reaction. a, Schematic of the experimental setup of the electrochemical cells, consisting of a Pt counter electrode and multilayer graphene membrane connecting with Cu working electrode; the graphene membrane is physically supported by a patterned photo-resist (PR) layer; electrodes are exposed to a 1.0 M $AuCl_3$ aqueous electrolyte, and a droplet of the same electrolyte is placed on the graphene membrane. An external load drives the Au-deposition reaction on the graphene membrane surface. **b,** Photographs (top) and schematic of graphene membrane. **c,** FEA simulation of potential distribution across the cell. Bottom panel: enlarged image taken from the square in the left panel showing that the potential gradient is concentrated on the bottom surface of the graphene membrane. **d,** Photograph of the controlled experiment (left) with a graphene membrane on which a droplet of $AuCl_3$ aqueous electrolyte was placed (right). **e,** Optical-microscope images of bottom (left) and top (right) surfaces of the graphene membrane after Au-electrodeposition with an external bias of -1.8 V vs. SHE for 100 s, showing that Au-deposition occurred only on the bottom surface of the graphene membrane. This control experiment also demonstrates the validity of our approach.

To respond to the reviewer's comment, we added Supplementary Fig. S2 to our revised Supplementary Information. In addition, we included the FEA simulation result for the Controlled experiment I (CuO/Cu bowl experiment) in the revised Supplementary Fig. S1 (see Supplementary Fig. S1b,c).

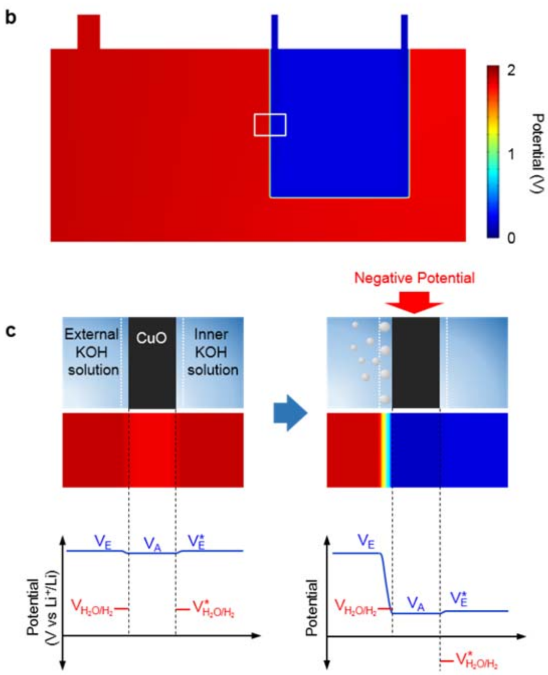

Supplementary Figure S1. b, FEA simulation of the potential distribution across the cell under an external voltage bias of 2V. **c**, Schematic and corresponding electrical potential profiles across the electrolyte and CuO/Cu anodes, taken from the square in **b**, without (left) and with (right) an external potential bias.

Comment (2): The authors need to add some SEM and TEM characterizations about the graphene PS before and after etching.

Author Response: As the reviewers suggested, we have performed the SEM and TEM characterizations of the NiSiNWs@GrμT sample at each preparation process. As shown in new Supplementary Fig. S4, although internal volume of the GrμT was slightly reduced after etching the core Ni foam, the GrμT could maintained the 3D tubular structure. In addition, TEM images identified that the GrμT walls consist of few-layer (five to seven layers) graphene, as coincident to the Raman characterization (see Fig. 2f and Supplementary Fig. S7 in our revised Supplementary Information). It is also noted that when the graphene-on-Ni foam was immersed in sulfuric acid solution to achieve the Ni islands@GrμT, Ni etching initiated along grain-boundaries of GrμT (Supplementary Fig. S4e).

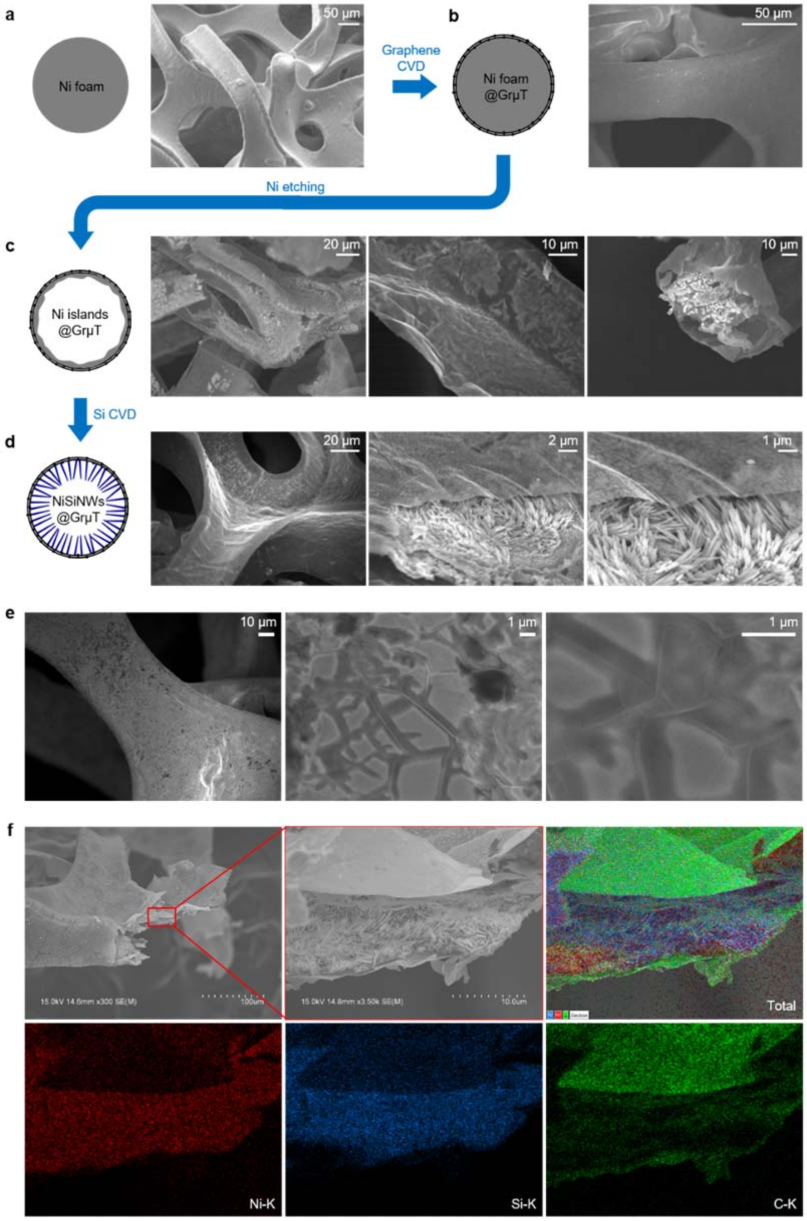

Supplementary Figure S4. Schematics and SEM images at each preparation step of NiSiNWs@GrμT. a-d, Ni foam (**a**), graphene-on-Ni foam (**b**), Ni islands@GrμT (**c**) and NiSiNWs@GrμTs (**d**). **e,** SEM images of the graphene-on-Ni foam taken during the inner Ni etching in the sulfuric acid solution. Ni etching initiated along the grain-boundaries of the GrμT. **f,** SEM-EDS elemental mapping images taken at the broken edges of the NiSiNWs@GrμT.

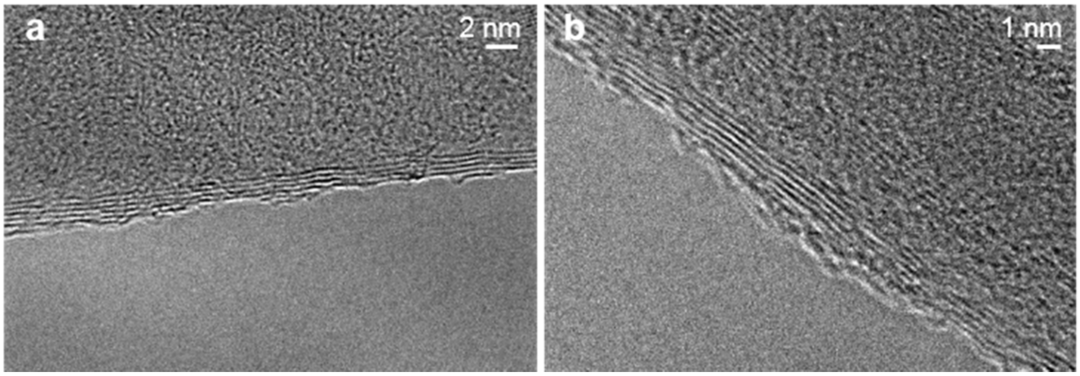

Supplementary Figure S7. TEM analyses of GrμT PS. a,b, HR-TEM images taken at the edges of GrμT walls collected from an Ni islands@GrμT sample, showing that the GrμT consist of few-layer graphene (five to seven layers).

To respond to the reviewer's comment, we added Supplementary Fig. S4 & S7 to our revised Supplementary Information.

Comment (3): Why exist a plateau at 1.4 V in the galvanostatic charge-discharge (GCD) profiles? I think it's not SEI formation because the electrolyte used (1 M LiPF6 in EC/EDC) always decomposes below 1V.

Author Response: Based on the critical comment of the reviewer, we have paid more attention to the analysis of the peak and plateau at 1.4 V in the cathodic scan (as well as those at 1.9 V in the anodic scan) observed in the CV and GCD profiles, respectively. In former times, we have considered that the peak/plateau formed at 1.4 V are associated with SEI formation, considering the decomposition potential (LUMO level) of our electrolyte of ~1.3-1.4 V vs Li^+/Li (*Chem. Mater., 2010, 22, 587-603*).

However, through literature survey and additional experiments, we came to the conclusion that the peak/plateau at 1.4 V and 1.9 V are not associated with SEI formation, but associated with the lithiation and delithiation of nickel sulfide residues with the following reasons: (1) Similar peak/plateau at 1.4 V and 1.9V in the CV and GCD profiles were observed from the nickel sulfide anode by other research groups (e.g., *Small 9, 8, 1321–1328, 2013* & *J. Mater. Chem., 19, 7277-7283, 2009*; see also Fig. R2a,b). (2) the substantially identical behaviors were observed from the nickel-containing samples that were underwent sulfuric acid treatment (Supplementary Fig. S8 in our revised Supplementary Information). (3) Sulfur was detected in the GrμT flake by TEM/EDS analysis, which shows that nickel sulfide residues were developed during the nickel etching process using sulfuric acid (Fig. R2c). Thanks to the previous report, we also came to know that the delithiation potential of nickel sulfide shifted towards higher voltage with cycles and exceeded the upper limit of voltage window (2V). This fact is useful to understand the rapid capacity fading that tends to occur in the NiSiNWs@GrμTs anodes in the early stage of cycles.

REDACTED

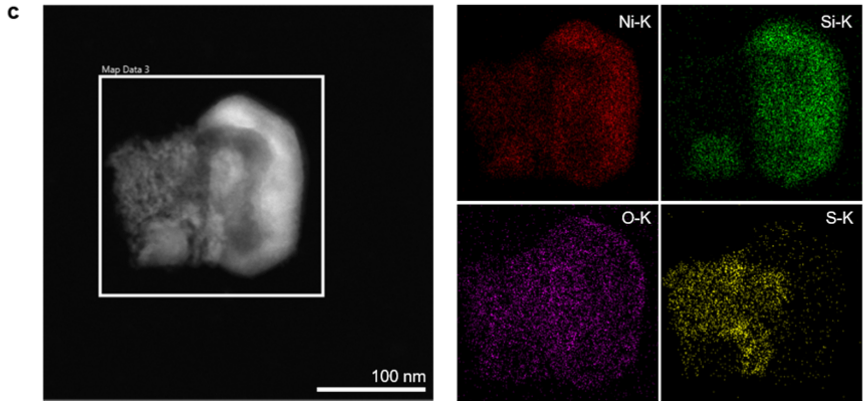

Figure R2. REDACTED c, TEM-EDS analysis of a GrμT flake collected by sonication of NiSiNWs@GrμT.

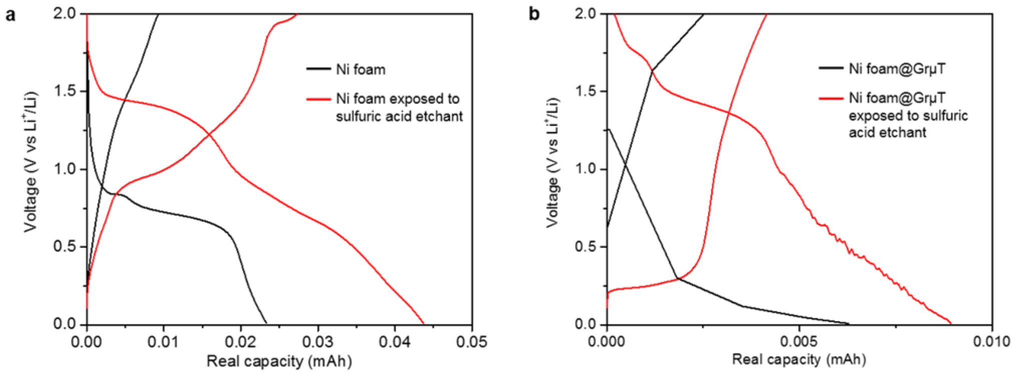

Supplementary Figure S8. Evolution of galvanostatic charge-discharge profiles depending on sulfuric acid treatment. Both the Ni foam (a) and Ni foam@GrμT exhibited new plateaus at 1.4 V and 1.9 V in the galvanostatic charge-discharge (GCD) profiles only after sulfuric acid treatment.

We thank the reviewer for the valuable comments which provides us the opportunity to remedy our wrong analysis. To address this issue, we added Supplementary Fig. S8 to our revised Supplementary Information. In addition, we revised the related paragraph as follows (1st paragraph on page 8) added the relevant references (ref. 26 & 27):

"In contrast, other peaks at 1.4 V in the cathodic scan and at 1.9 V in the anodic scan diminish gradually during the scans. We can also observe plateaus at 1.4 V and 1.9 V in the galvanostatic charge-discharge (GCD) profiles, displaying similar features in the charging and discharging stages, respectively, and almost disappear after 10-20 cycles (Fig. 3b). The nearly identical behaviors were observed from the nickel sulfide anode[26,27] and the samples that underwent sulfuric acid treatment (Supplementary Fig. S8). Thus, we conclude that the plateaus/peaks at 1.4 V and 1.9 V are associated with the lithiation and delithiation of nickel sulfide residues that were developed during the nickel etching process using sulfuric acid, respectivly. It is noteworthy that the lithiation plateaus disappeared rapidly during the initial several cycles (Fig. 3b). Since the delithiation potential of nickel sulfide shifted toward higher voltage with cycles and exceeded the upper limit of the voltage window (2V)[27], the more lithiated nickel sulfide could not return to a delithiated state with cycles. This mechanism is closely linked to the rapid capacity fading that tends to occur in NiSiNWs@GrμT anodes in the early stage of cycles (see the red and blue plots in Figs. 3 c-e)[28]. Nevertheless, once the anodes were stabilized, there was little change in the charge/discharge profile after 100 cycles at 10C, and the charge/discharge capacities were maintained above 900 $mAhg^{-1}$."

"ref 26. Lai, C. H. *et al.* Oriented growth of large-scale nickel sulfide nanowire arrays via a general solution route for lithium-ion battery cathode applications. *J. Mater. Chem.* **19**, 7277-7283, doi:10.1039/b909261g (2009)."

&

"ref 27. Mahmood, N., Zhang, C. Z. & Hou, Y. L. Nickel Sulfide/Nitrogen-Doped Graphene Composites: Phase-Controlled Synthesis and High Performance Anode Materials for Lithium Ion Batteries. *Small* **9**, 1321-1328, doi:10.1002/smll.201203032 (2013)."

Comment (4): One hypothesis is that Ni may not be completely removed in the process of Ni etching. After growth of NiSi nanowries, still some Ni existed inside the graphene, so it can enhance the electronic conductivity. Maybe that's the important cause for such a good rate performance of this structure. I think it need some experimental data to support the author's result (ie. SEM eds mapping, TEM eds mapping including the graphene part).

Author Response: We respectively disagree with the reviewer's comment that some residual Ni inside the GrμT contributes largely to the rate performance of our anode with the following reasons. First, in the case of NiSiNWs-on-NiF anode, all the NiSiNWs are connected to the highly-conducting Ni foam, but it cannot withstand high-rate cycling exceeding 10C. As the reviewer claimed, some Ni islands existed inside the graphene could improve the electrical conductivity, but its contribution to the total electrical conduction would be insignificant given that the Ni islands are not continuously connected (see Supplementary Fig. S4f). When we measured the resistance of the GrμTs with and without Ni islands, no significant difference in electrical resistance was found (GrμTs with ($\rho = 0.12$ $\Omega\cdot$cm) and without ($\rho = 0.14$ $\Omega\cdot$cm) Ni islands; similar electrical conductivity was observed in *Nature Materials, 2011, 10, 424-428*). We have further performed the SEM/TEM-EDS characterizations of the NiSiNWs@GrμTs and achieve more data that can support our claim that our "potential shielding mechanism" could effectively inhibit the SEI formation on the

NiSiNW surface (see our response to the reviewer 2's comment 4). We believe that this phenomenon plays a critical role in achieving high rate capability.

To address this issue, we added the EDS elemental mapping images (Supplementary Fig. S4f) to our revised Supplementary Information.

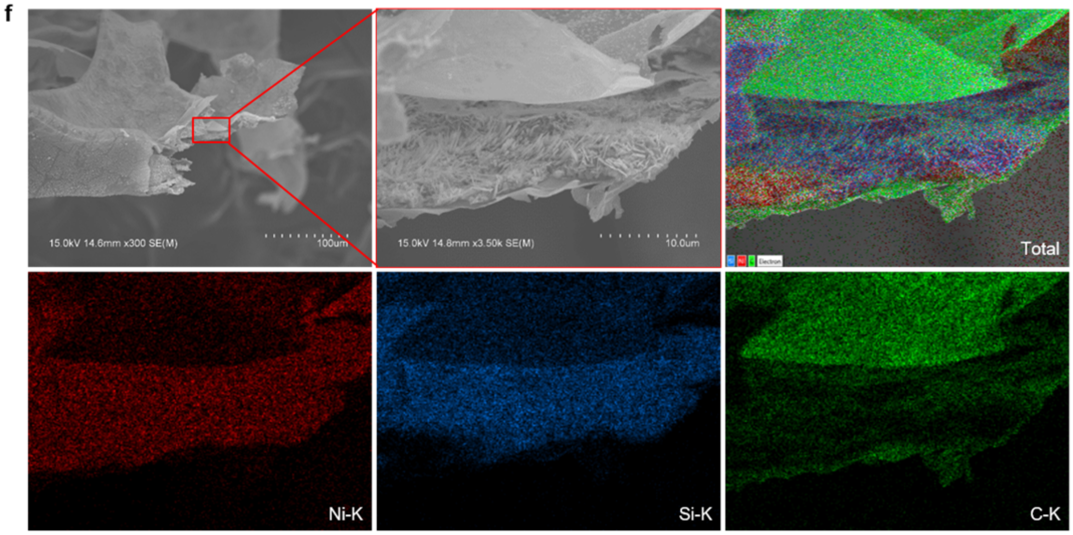

Supplementary Figure S4f, SEM-EDS elemental mapping images taken at the broken edges of the NiSiNWs@GrµT.

Comment (5): Too many mistakes in the manuscript. For example, Page 6 line 22, "while the diameter and length of NiSiNWs are ~8-10 µm and 80-150 nm, respectively."; the order of a b c d in the figure 4 caption is totally confused.

Author Response: We thank to the reviewer for pointing out our mistakes. Following the reviewer's comment, we corrected those mistakes (see 2nd paragraph on page 7 and the legend caption of Fig. 4 in our revised manuscript).

Reviewer #2 (Remarks to the Author):
This manuscript describes a new method designed to control the SEI formation in Si-based nanowires used as anodes for Li ion batteries. The designed is explained as a way to control the potential to which the nanowires are exposed in contact with the electrolyte solution. However there are several points that are not clear and should be addressed before the article can be reconsidered for publication.

Author Response: We thank the reviewer for the thoughtful comments about our manuscript. We welcome the opportunity to address and clarify the issues raised in the reviewer's report, and believe that the additional experiments and revisions substantially strengthen our manuscript. Our responses to the points raised in the report are below.

Comment (1): In the new design the Li ions should go through an "atomically thin" wall named "potential sheath" (PS). This PS is made of graphene. Li ions cannot go through the aromatic rings unless there are defects. How can the ions go through to a perfect graphene layer?

Author Response: As the reviewer pointed out, it is known that Li-ions/atoms cannot penetrate through perfect atomic lattice of graphene. However, CVD-grown multi-layer graphene on Ni foil/foam generally has polycrystal structure with many native defects such as grain boundaries and atomic vacancies. These defects provide the pathway for the transport of many types of atoms/ions/gas molecules, as reported in the literatures (e.g., *Nanoscale, 2014, 6, 151-156* & *Sci. Adv., 2016, 2, e1501272*). In addition, LIB anodes exploiting active materials encapsulated/covered by graphene shells showed that lithium could penetrate through the graphene shells and enter into active materials (see *Nat. Commun., 2017, 8, ARTN 13949* & *Acs Nano, 2017, 8, 1728-1738*). During the fabrication of NiSiNWs@GrμTs, we also found that when the graphene-on-Ni foam was immersed in sulfuric acid solution to achieve the Ni islands@GrμT, Ni etching initiated along grain-boundaries of GrμT (Supplementary Figure S4e).

In view of these considerations, it can be concluded that the defects such as grain boundary provide the pathway for Li-insertion to the graphene PS and transport to the NiSiNWs anchored to the inner surface of the GrμTs.

e

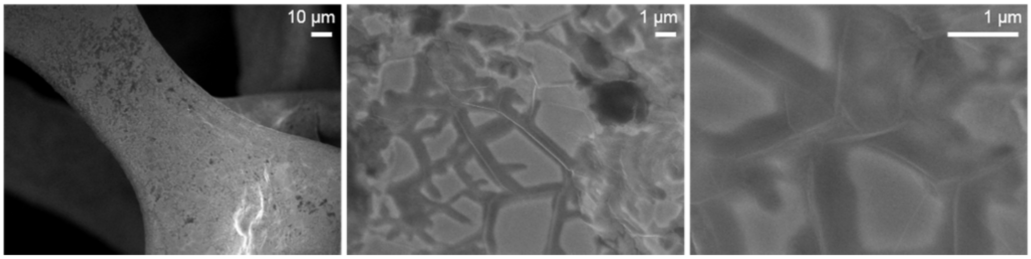

Supplementary Figure S4e. SEM images of the graphene-on-Ni foam taken during the inner Ni etching in the sulfuric acid solution. Ni etching initiated along the grain-boundaries of the GrμT.

To respond to the reviewer's comment, we added Supplementary Fig. S4e to our revised Supplementary Information added the relevant references (ref. 17 & 18).

"ref. 17. Cheng, C. *et al.* Ion transport in complex layered graphene-based membranes with tuneable interlayer spacing. *Sci. Adv.* **2**, doi:ARTN e1501272, 10.1126/sciadv.1501272 (2016)."

&

"ref. 18. Yoon, T., Mun, J. H., Cho, B. J. & Kim, T. S. Penetration and lateral diffusion characteristics of polycrystalline graphene barriers. *Nanoscale* **6**, 151-156, doi:10.1039/c3nr03849a (2014)."

Comment (2): Page 5. It is suggested that the potential inside the space where the nanowire interfaces with the electrolyte remains lower than V_A, and therefore the SEI does not form. It is not clear why**.** How does the potential of the nanowire, NiSi, varies upon lithiation? The authors should provide information about this lithiation and the voltage of Li intercalation in NiSi. That would clarify what is the potential. Probably all the results can be explained by the use of this alternative material, NiSi.

Author Response: Since the external electrolyte forms a loop with the Li metal counter electrode, the potential profiles across the external electrolyte and NISiNWs@GrμT anode connected to working electrode can be determined on the basis of the Li metal counter electrode (the potential at each position can be given by the unit of V vs Li^+/Li). It was reported that the redox potential for (de)lithiation of NiSi was in the range of ~0.0-0.3 V (*Electrochem. Comm., 2011, 13, 546–549*), and this is coincident to the CV profile achieved in this study (Fig. 3a).

On the other hand, the enclosed space of the GrμT is potentially isolated; in this situation, the potential difference over the electrode and electrolyte inside remains constant (which is determined by contact potential difference), regardless of the external load bias (see also our response to the reviewer 1's comment 1 for detailed discussion about this issue).

Comment (3): I don't understand how FEA simulations "can predict" voltages. They will only reproduce voltages given as input. Please explain. If the profile shown in Figure 1d (right) were correct, the nanowires wouldn't be able to be lithiated.

Author Response: As the reviewer commented, the FEA simulation cannot predict the voltage difference between two electrodes; we can measure the potential for certain electrochemical reactions (e.g., potential of SEI formation, V_{SEI}, and (de)lithiation potential, V_{Lith}) experimentally (e.g., by CV profile), while position dependent voltage difference across each component in the electrochemical cell can be simulated by FEA modeling. Based on those information, we can produce position-dependent potential (V vs Li^+/Li) curves (e.g., lower panels in Fig. 1c and d).

Meanwhile, as pointed out by the reviewer, there is no significant potential gradient at the interface between the inner electrolyte and NW, even at $V_A < V_{Lith}$ (Fig. 1d), and thus any electrochemical reaction (SEI formation, lithiation) does not occurs at the inner electrolyte and NW interface for the case of NW anode enclosed by PS. Instead, as schematically illustrated in upper right panel of Fig. 1d, SEI formation and lithiation reactions take place at the interface between the external electrolyte and the PS where a potential gradient is

concentrated. Then, the intercalated Li diffuses to the NWs anchored to the inner surface of the PS.

For Li diffusion, the NWs inside the PS would have a disadvantage considering that the long diffusion pathway. However, it is noteworthy that in-situ TEM studies showed the fast lithiation kinetics in individual NWs, in which Li insertion occurred at one end of the NWs and followed by Li transport along axial direction with axial speed up to ~213 nm/s for highly-conducting SiNWs (ref 31, Fig. R3a,b). We have also carried out FEA simulation for this case, which shows that the potential gradient is concentrated at the interface between tip of the NW and electrolyte (Li_2O), as similar to case of our NISiNWs@GrμT (Fig. R3c).

REDACTED

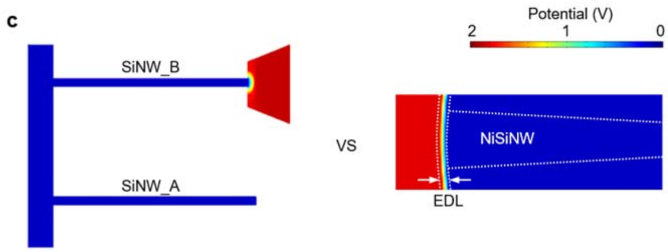

Figrue R3. **[REDACTED]** **c,** FEA simulation for the case of (a) shows that the potential gradient is concentrated at the NW tip/electrolyte interface as similar to the case of our NISiNWs@GrμTs.

To address this issue clearly, we revised the related paragraph as follows (2nd paragraph on page 11):

"However, previous in-situ TEM studies showed fast lithiation kinetics in individual NWs, in which Li insertion occurred at one end of the NWs and was followed by Li transport along the axial direction, similar to our situation. In some cases, the overall lithiation rate was not axial transport limited, but rather determined by the radial insertion of Li into the NW core[34]. This is due to the much faster Li transport along the surface than that in the bulk, enabling ultrafast lithiation with an axial speed up to ~213 nm/s for highly-conducting SiNWs[35]. In this regard, we conclude that the Li diffusion in our NW anode does not critically limit the cycling capacity even at higher rate (e.g., the axial lithiation speed of 200 nm/s can fully lithiate ~8 μm-long SiNWs at 80C). Nonetheless, further study is needed to elucidate the lithiation kinetics in our system."

Comment (4): The structure of the nanowires (presence of porous Ni) can explain the smooth behavior during lithiation. The authors should present further characterization of the structure of the nanowires and lithiation behavior. Some publications (JES 154, A97-A102) have suggested that during lithiation NiSi undergoes reversible transformation to/from Ni2Si and LiySi. This transformation has a change of volume opposite to lithiation which may stabilize the system and make the SEI more stable due to the absence of a significant volume change. But it is hard to believe that a SEI is not formed over the nanowire. The authors should review their explanation once these details are clarified.

Author Response: reviewer: In the literatures, the reversible electrochemical reactions in Li/NiSi cells have been described by either one of the following equations:

$$2NiSi + xLi^{+} \leftrightarrow Li_xSi + Ni_2Si \qquad [1]$$

$$4NiSi + 22Li^{+} \leftrightarrow Li_{22}Si_5 + 4Ni \qquad [2]$$

Theoretical capacities of the NiSi after the reaction [1] and reaction [2] are expected to be ~580 mAh/g and ~1300 mAh/g, respectively. In the paper that reviewer introduced (*JES 154, A97-A102*), it is claimed that equation [1] is the (de)lithiation reaction, in which lithiation NiSi undergoes reversible transformation to/from Ni2Si and LiySi and compensates the volume expansion. On the other hand, most other papers claim that the (de)lithiation reaction occurs via equation [2] that resulted in ~200% volume expansion upon lithiation (*Electrochem. Comm., 2011, 13, 546–549 & J. Alloy. Comp., 2000, 306, 249–252 & J. Mater. Chem. A, 2014, 2, 9156*). In our cells, we also observed ~190% volume expansion upon lithiation (Fig. 4b) and the capacity exceeded largely the theoretical capacity of the reaction equation [1], indicating that the (de)lithiation reaction of our NiSiNWs follows the equation [2]. In addition, the formation of very thick SEI layers on the NiSiNWs-on-NiF was observed, which excludes the possibility that the absence of a significant volume change in the lithiated NiSi enables more stable SEI formation on the NiSiNWs.

Furthermore, to confirm that a SEI is not formed over the NiSiNWs inside the GrµTs, we have further performed the SEM/TEM-EDS characterizations of the NiSiNWs@GrµTs (Supplementary Fig. S12 & S13 in our revised Supplementary Information; see our response to the reviewer 3's comment 2). These characterizations support our claim that our approach exploiting 'potential shielding' effect inhibits SEI formation on the NiSiNW surface, thereby enabling unprecedentedly excellent cycling capability.

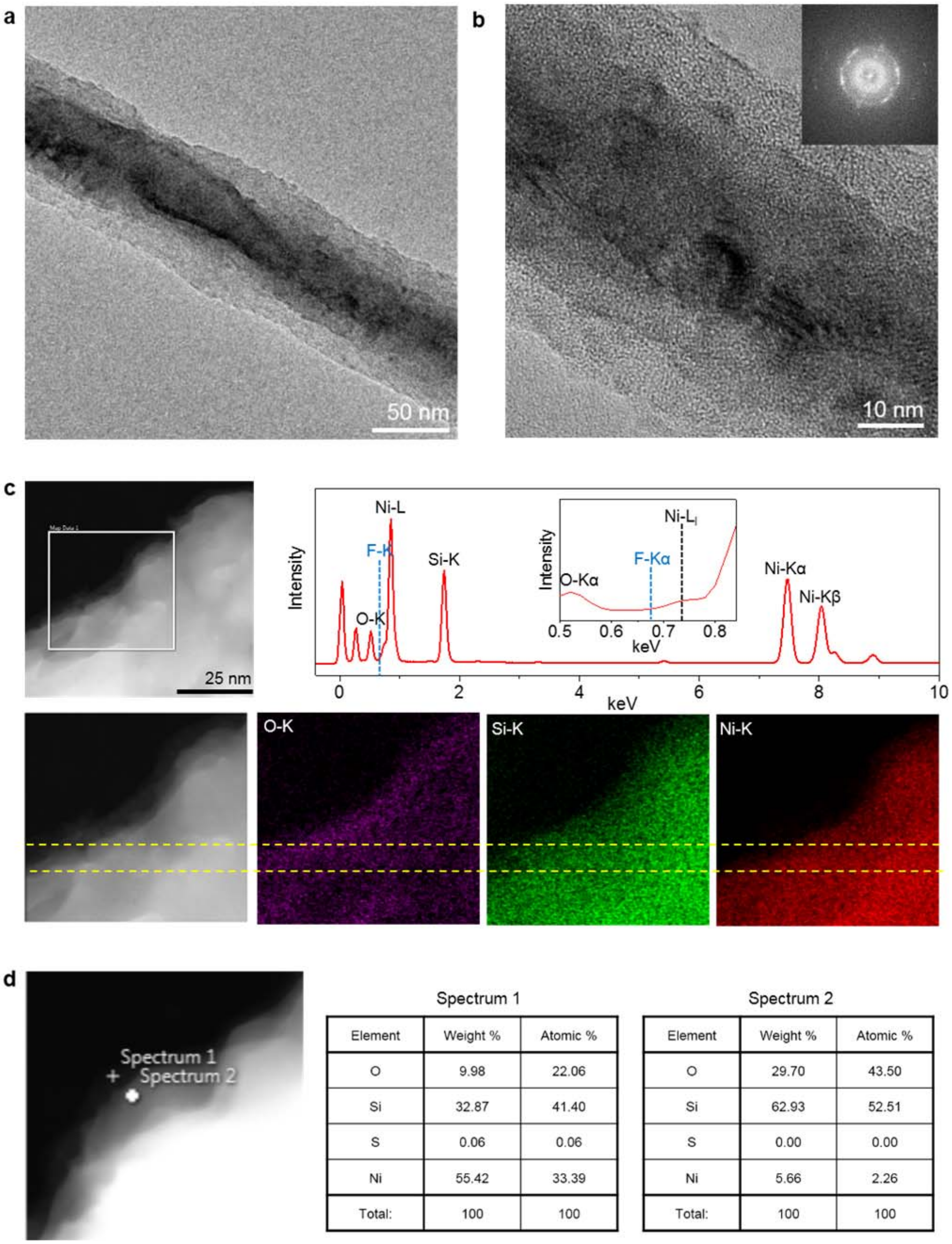

Spectrum 1

Element	Weight %	Atomic %
O	9.98	22.06
Si	32.87	41.40
S	0.06	0.06
Ni	55.42	33.39
Total:	100	100

Spectrum 2

Element	Weight %	Atomic %
O	29.70	43.50
Si	62.93	52.51
S	0.00	0.00
Ni	5.66	2.26
Total:	100	100

Supplementary Figure S12. TEM analysis of delithiated NiSiNW. a,b, HR-TEM image of NiSiNW collected from delithiated NiSiNWs@GrμT after 500 cycles at 10C. Lattice-resolved image and selective area electron diffraction pattern (inset in b) confirm the poly-crystal structure of the NiSiNW. **c**, EDS element analysis of delithiated NiSiNW, taken along the area in the upper-left panel of the dark-field image of the NiSiNW. Upper-right panel: EDS spectrum and elemental mapping images (bottom panels) show that outer shell of the NiSiNW consists mainly of SiOx without SEI; the P and F associated with the SEI layer component are not detected within the detection limit. d, Summaries of elemental compositions at the crossbar points.

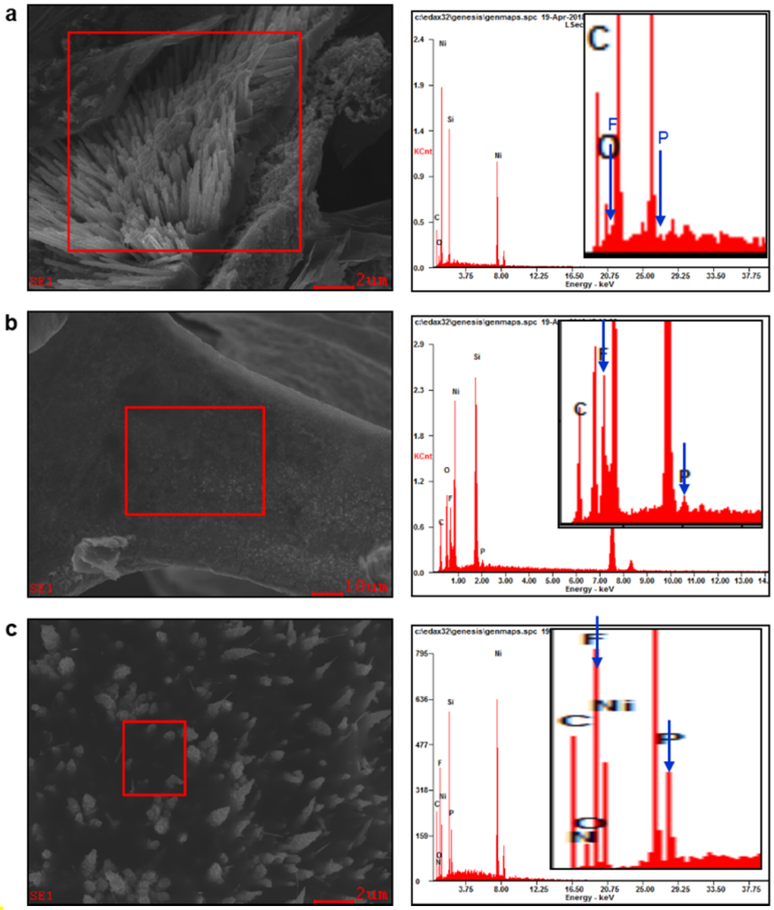

Element	Wt%	At%
CK	32.52	63.81
OK	3.08	4.53
FK	0.74	0.92
SiK	11.81	9.91
PK	0.06	0.04
NiK	51.80	20.79
Matrix	Correction	ZAF

Element	Wt%	At%
CK	26.27	46.79
OK	14.84	19.84
FK	7.94	8.94
SiK	14.37	10.95
PK	0.47	0.32
NiK	36.11	13.16
Matrix	Correction	ZAF

Element	Wt%	At%
CK	28.95	56.88
NK	2.52	4.25
OK	1.82	2.69
FK	6.36	7.90
SiK	7.04	5.91
PK	2.60	1.98
NiK	50.71	20.38
Matrix	Correction	ZAF

Supplementary Figure S13. SEM image and EDS analysis. a-c, SEM images (left), EDS spectra (middle) and summaries of the elemental composition (right) of the delithiated NiSiNWs-on-NiF (**a**) and delithiated NiSiNW@GrμT (**b,c**). In **c**, GrμT was opened to analyze the NiSiNWs inside. The blue arrows in the insets of the middle panels indicate the positions of F K-series (left) and P K-series (right) transitions.

To response the reviewer's comment, we added Supplementary Fig. S12 & S13 and the relevant references (ref. 20 & 21 & 22) to our revised Supplementary Information.

"ref. 20. Wang, G. X. *et al.* Nanocrystalline NiSi alloy as an anode material for lithium-ion batteries. *J. Alloy. Compd.* **306**, 249-252, doi:Doi 10.1016/S0925-8388(00)00775-1 (2000)."

&

"ref. 21. Zhou, Y. N. *et al.* Nanostructured NiSi thin films as a new anode material for lithium ion batteries. *Electrochem. Commun.* **13**, 546-549, doi:10.1016/j.elecom.2011.03.006 (2011)."

&

"ref. 22. Li, F. *et al.* In situ coating of NiO on Ni-silicide nanowires with roughened surfaces for improved electrochemical energy storage. *J. Mater. Chem. A* **2**, 9156-9163, doi:10.1039/c4ta01171f (2014)."

Reviewer #3 (Remarks to the Author):
This manuscript reports the fabrication of silicide nanowires (NWs) anchored selectively to the inner surface of micro-tubular graphene electrodes through the selective etching followed by CVD process. The authors further claimed that this structure design could electrically shields the electrolyte from an external potential load, eliminating the driving force that generates the SEI on the NW surface during lithiation. Although we agree with the novelties in terms of material design and good cycle performance reported in this manuscript. However, the established "potential shielding" model are not convincing simply based on the electrolytic water experiment shown in the supporting information. Additionally, the characterization revealed some noticeable issues yet to be revised.

Author Response: We thank the reviewer for the thoughtful comments about our manuscript. We welcome the opportunity to address and clarify the issues raised in the reviewer's report, and believe that the additional experiments and revisions substantially strengthen our manuscript. Our responses to the points raised in the report are below.

Comment (1): Firstly, the so-called "potential shielding" effect for the NiSi NWs in the electrolyte is not comparable to the electrolytic water experiment (supporting information) in which the solution inside of the copper crucible do not form a complete loop with external electric load. Since this part of solution does not necessarily participate in the reaction, FEA simulation results for the potential gradient develops formed on the surface of the graphene PS provides limited comparative meaning.

Author Response: As the reviewer pointed out, the electrolyte solution inside the CuO/Cu bowl is physically and electrically isolated/separated from the outside electrolyte; accordingly, the inside electrolyte solution is not affected by external electric load (i.e., potential gradient across the CuO/Cu bowl remains unchanged, regardless of the external bias; see Fig. R4a). This would be identical to the reviewer's claim that the inner electrolyte solution does not form a complete loop with external electric load. However, if one side of the CuO/Cu bowl is replaced with a thin dielectric wall, the inner electrolytes is physically separated but not electrically isolated since the dielectric wall cannot fully screen the penetration of electric field (Fig. R4b).

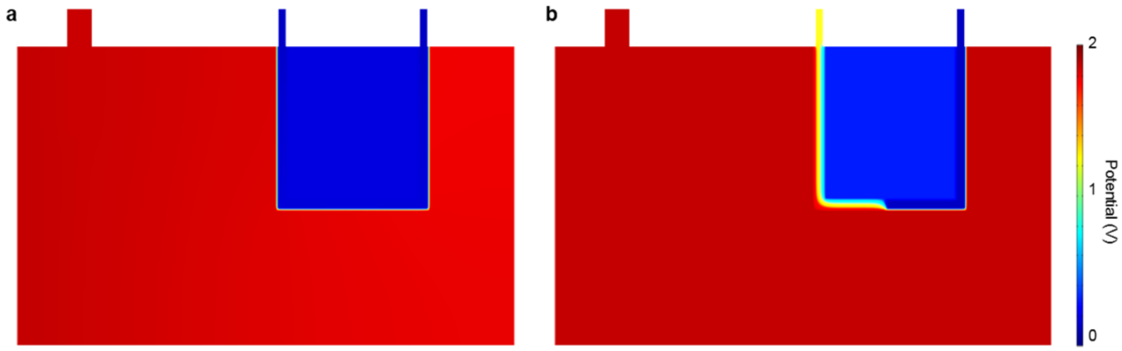

Figure R4. FEA simulation of CuO/Cu bowl (**a**) and CuO/Cu, of which one side is replaced with a thin dielectric wall (**b**).

Meanwhile, given that the ion and electrolyte are not permeable in the CuO/Cu bowl while they are permeable in the GrμT, the CuO/Cu bowl and GrμT may have a different

characteristic as potential sheath; however, the GrμT ends are sealed so that the electrolytes inside and outside of the GrμT do not directly contact each other. In addition, potential becomes nearly equivalent across the GrμT due to good electron conduction, and thus most of the potential gradient is created in the EDL developed at the interface of electrolyte outside and the GrμT. Accordingly, the GrμT and the CuO/Cu bowl electrodes produce the similar potential profile across the outer electrolyte/electrode/inner electrolyte (see Fig. R1 and Supplementary Fig. S1b,c in our revised Supplementary Information). It is also noteworthy that even nanoscale cracks exist in the PS, the penetration of electric potential into inside of the GrμT could be effectively suppressed so that the potential gradient inside would not be enough to initiate the SEI formation (see Supplementary Fig. S3b, c in our revised Supplementary Information).

In this situation, given that most electrochemical reactions (e.g., SEI formation, lithiation, hydrogen evolution, electrodeposition) are governed by a potential gradient, the Cu bowl experiment could be useful to explain the mechanism of our approach exploiting electric potential control to prevent SEI formation on the NW surface.

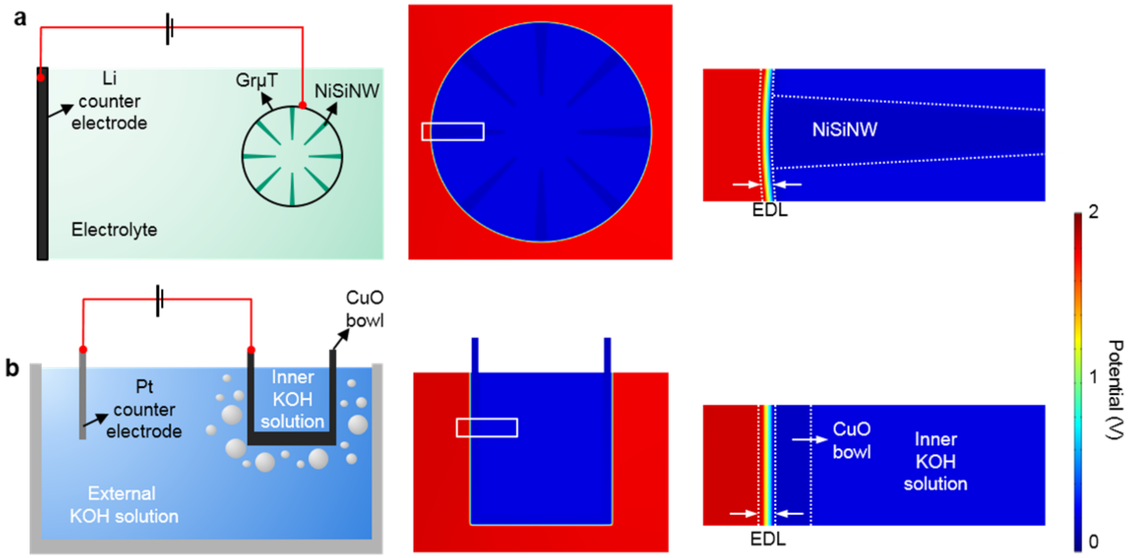

Figure R1. Similarity of potential profile across the outer electrolyte/electrode/inner electrolyte between the NiSiNWs@GrμT and CuO/Cu bowl anodes of the electrochemical cells.

However, to address the concern that reviewer raised, we have conducted another controlled experiment (II) using multi-layer graphene, instead of CuO/Cu bowl, as PS (see Supplementary Fig. S2 in our revised Supplementary Information). The multi-layer graphene membrane that was supported by patterned photoresist layer was transferred onto a 1.0 M $AuCl_3$ aqueous electrolyte and a droplet of same electrolyte is placed on the graphene membrane to form additional interface on the top surface of the graphene (actual lithiation experiments were replaced by electrodeposition experiments due to the practical limits such as safety issue and oxidation of lithium metal & electrolyte.) As shown in Supplementary Fig. S2a, between a Pt counter electrode and Cu electrode connected to one end of the graphene

membrane, an external load was applied to drive the Au electrodeposition reaction at the electrolyte and graphene interface. After the electrodeposition with an external bias of -1.8 V vs V_{SHE} for 100 s, we found that Au-deposition occurred only on the bottom surface of the graphene membrane where the potential gradient is created. This control experiment also demonstrates the validity of our approach (i.e., the SEI formation and Li insertion occur only on the external electrolyte/GrμT interface where the potential gradient is created).

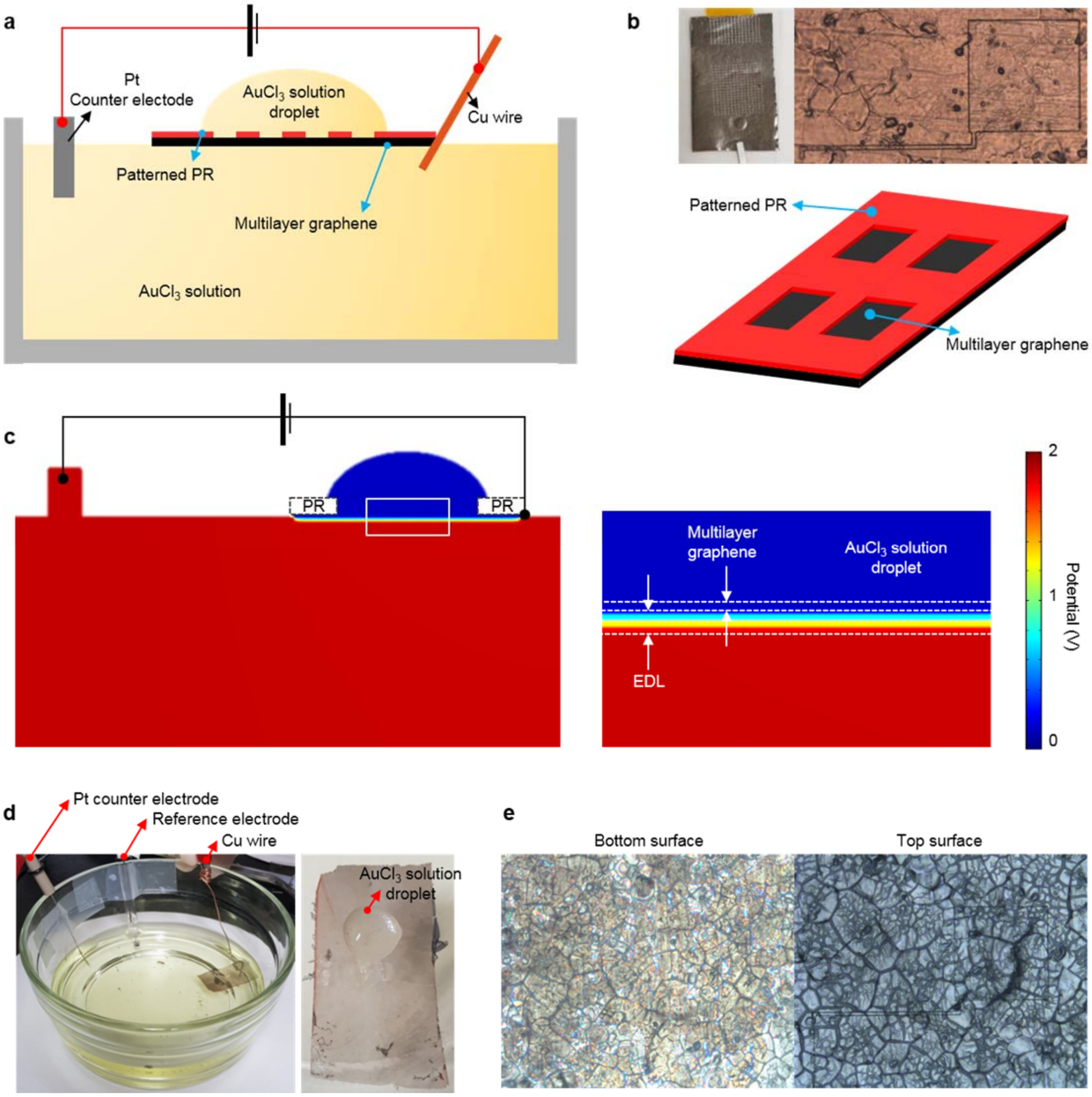

Supplementary Figure S2. Controlled experiment (II) illustrating electric potential control of the electrodeposition reaction. a, Schematic of the experimental setup of the electrochemical cells, consisting of a Pt counter electrode and multilayer graphene membrane connecting with Cu working electrode; the graphene membrane is physically supported by a patterned photo-resist (PR) layer; electrodes are exposed to a 1.0 M $AuCl_3$ aqueous electrolyte, and a droplet of the same electrolyte is placed on the graphene membrane. An external load drives the Au-deposition reaction on the graphene membrane surface. **b,** Photographs (top) and schematic of graphene membrane. **c,** FEA simulation of potential distribution across the cell. Bottom panel: enlarged image taken from the square in the left

panel showing that the potential gradient is concentrated on the bottom surface of the graphene membrane. **d,** Photograph of the controlled experiment (left) with a graphene membrane on which a droplet of $AuCl_3$ aqueous electrolyte was placed (right). **e,** Optical-microscope images of bottom (left) and top (right) surfaces of the graphene membrane after Au-electrodeposition with an external bias of -1.8 V vs. SHE for 100 s, showing that Au-deposition occurred only on the bottom surface of the graphene membrane. This control experiment also demonstrates the validity of our approach.

To respond to the reviewer's comment, we added Supplementary Fig. S2 to our revised Supplementary Information. In addition, we included the FEA simulation result for the Controlled experiment I (CuO/Cu bowl experiment) in the revised Supplementary Fig. S1 (see Supplementary Fig. S1b,c).

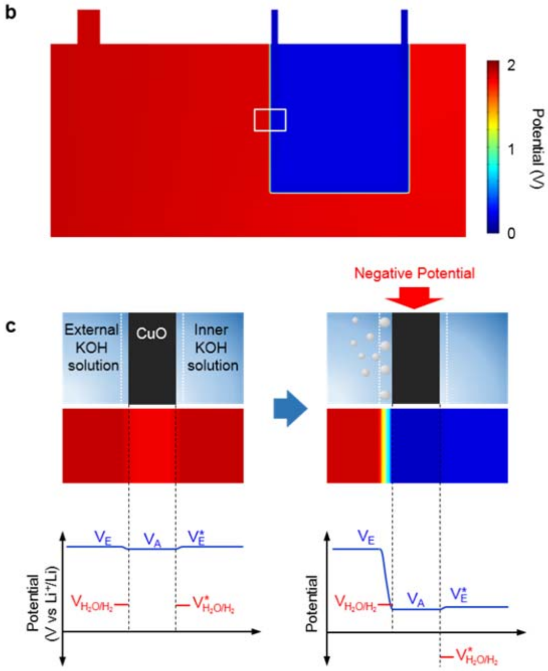

Supplementary Figure S1. b, FEA simulation of the potential distribution across the cell under an external voltage bias of 2V. **c**, Schematic and corresponding electrical potential profiles across the electrolyte and CuO/Cu anodes, taken from the square in **b**, without (left) and with (right) an external potential bias.

Comment (2): Secondly, the characterizations results cannot confirm that SEI layer develops only on the outer surface of the Grμ T-based PS, thus the statement of the SEI-free NiSiNWs after cycling is problematic. In the figure 4c, the post-mortem NiSiNWs@GrμTs demonstrate an amorphous layer on the NW surface which seems to be SEI layer. Although the authors claim this is the amorphous oxide layer formed on the SiNi NW, the elemental EDS mapping is needed.

Author Response: Considering reviewer's concern, we have performed more thorough EDS characterizations of the NiSiNWs@GrμT that underwent delithiation after 500 cycles at 10C. As shown in Supplementary Fig. S12a in our revised Supplementary Information, the

NiSiNWs sustained their original 1D morphology and the size. In addition, TEM-EDS characterizations (EDS spectrum and elemental mapping in Supplementary Fig. S12c,d) showed that the outer amorphous layer on the NW surface consists mostly of Si and O elements, and the P and K elements that are associated with SEI were not detected within the detection limit (upper-right graph in new Supplementary Fig. S12c).

The SEM-EDS analysis on the NiSiNWs inside the GrμT showed the similar result (Supplementary Fig. S13a in our revised Supplementary Information). In contrast, the EDS peaks assigned to the F K-series (left) and P K-series (right) transitions are clearly observed on the outer surface of the GrμT (Supplementary Fig. S13b) and on the NiSiNWs-on-NiF (Supplementary Fig. S13c). Moreover, it is clearly seen that a thick SEI layer covering the NiSiNWs was developed in the NiSiNWs-on-NiF anode after 100 cycles at 2C (Supplementary Fig. S14a).

These EDS analysis results more strongly support our claim that the "potential shielding mechanism" could effectively inhibit the SEI formation on the NiSiNW surface, leading to extraordinary high rate capability of the NiSiNWs@GrμT anode.

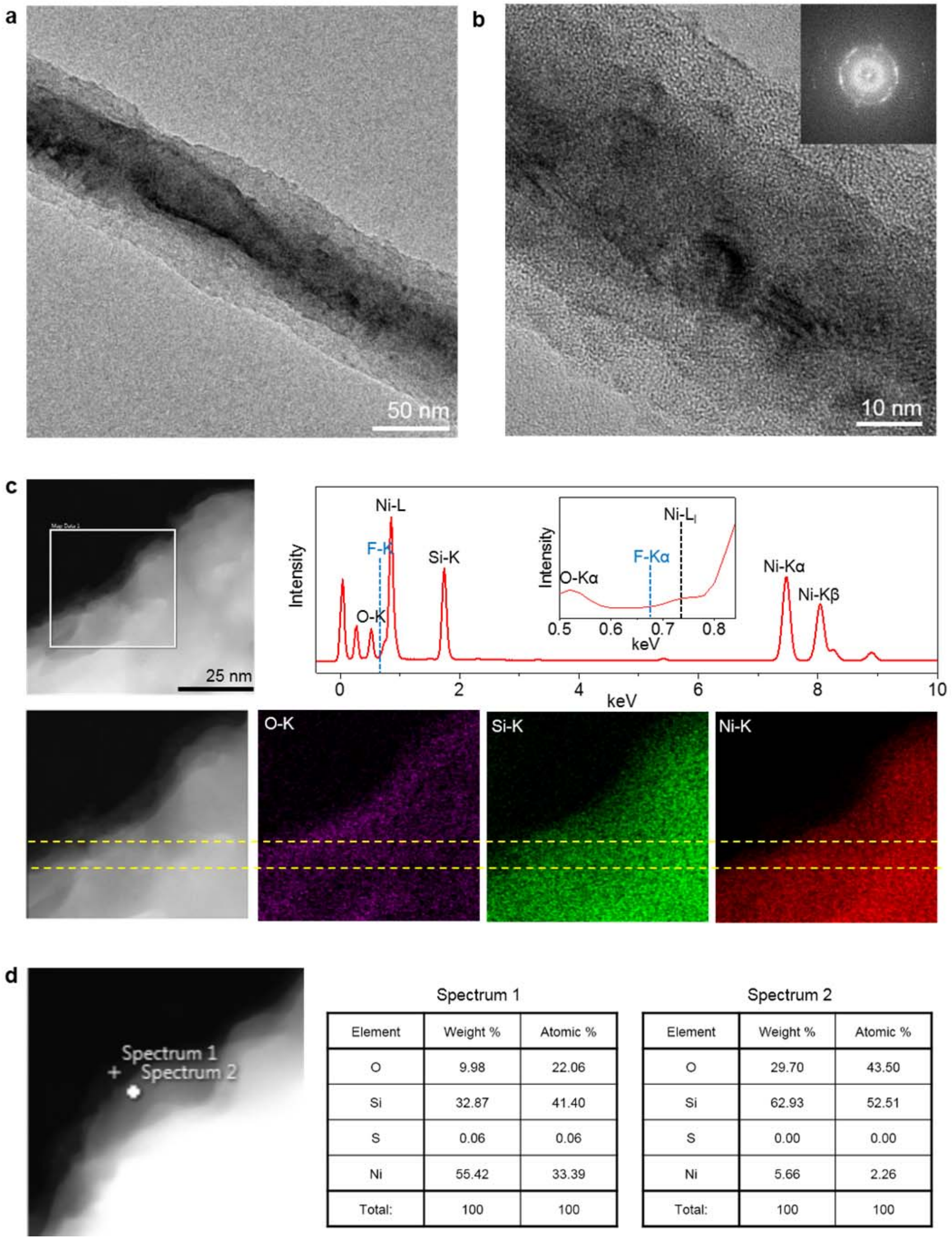

Spectrum 1

Element	Weight %	Atomic %
O	9.98	22.06
Si	32.87	41.40
S	0.06	0.06
Ni	55.42	33.39
Total:	100	100

Spectrum 2

Element	Weight %	Atomic %
O	29.70	43.50
Si	62.93	52.51
S	0.00	0.00
Ni	5.66	2.26
Total:	100	100

Supplementary Figure S12. TEM analysis of delithiated NiSiNW. a,b, HR-TEM image of NiSiNW collected from delithiated NiSiNWs@GrμT after 500 cycles at 10C. Lattice-resolved image and selective area electron diffraction pattern (inset in b) confirm the poly-crystal structure of the NiSiNW. **c**, EDS element analysis of delithiated NiSiNW, taken along the area in the upper-left panel of the dark-field image of the NiSiNW. Upper-right panel: EDS spectrum and elemental mapping images (bottom panels) show that outer shell of the NiSiNW consists mainly of SiOx without SEI; the P and F associated with the SEI layer component are not detected within the detection limit. d, Summaries of elemental compositions at the crossbar points.

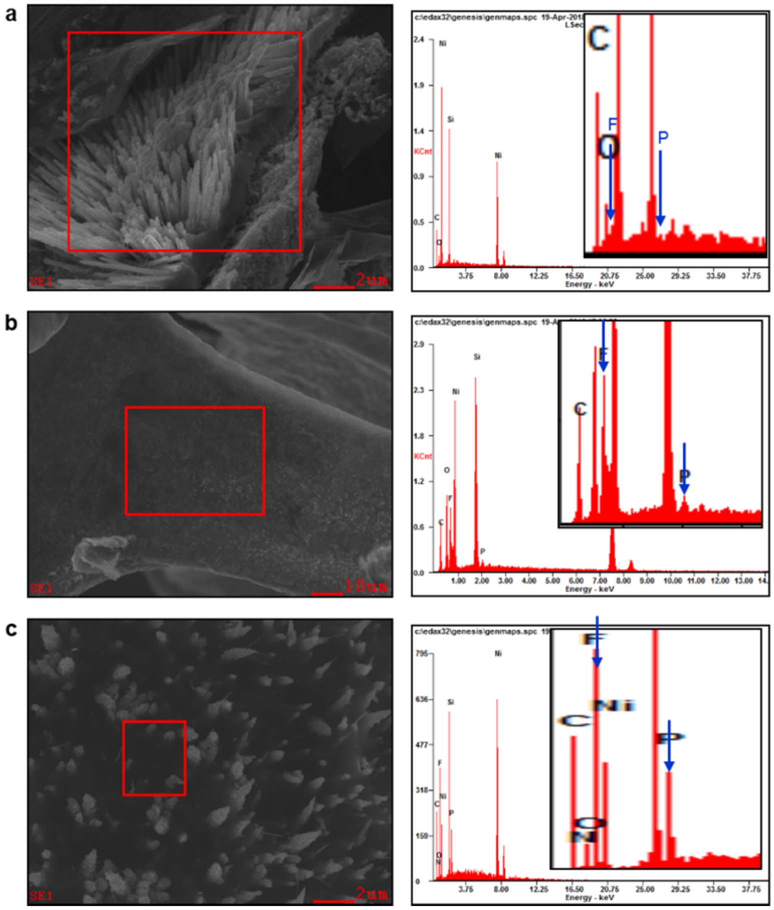

(a)

Element	Wt%	At%
CK	32.52	63.81
OK	3.08	4.53
FK	0.74	0.92
SiK	11.81	9.91
PK	0.06	0.04
NiK	51.80	20.79
Matrix	Correction	ZAF

(b)

Element	Wt%	At%
CK	26.27	46.79
OK	14.84	19.84
FK	7.94	8.94
SiK	14.37	10.95
PK	0.47	0.32
NiK	36.11	13.16
Matrix	Correction	ZAF

(c)

Element	Wt%	At%
CK	28.95	56.88
NK	2.52	4.25
OK	1.82	2.69
FK	6.36	7.90
SiK	7.04	5.91
PK	2.60	1.98
NiK	50.71	20.38
Matrix	Correction	ZAF

Supplementary Figure S13. SEM image and EDS analysis. a-c, SEM images (left), EDS spectra (middle) and summaries of the elemental composition (right) of the delithiated NiSiNWs-on-NiF (**a**) and delithiated NiSiNW@GrμT (**b**,**c**). In **c**, GrμT was opened to analyze the NiSiNWs inside. The blue arrows in the insets of the middle panels indicate the positions of F K-series (left) and P K-series (right) transitions.

To response the reviewer's comment, we added Supplementary Fig. S12 & S13 to our revised Supplementary Information.

Comment (3): Finally, What does the oxidation peak at around 1.9V and the reduction peak at ~ 1.3V represents in Figure 3a (CV curve)? A detailed explanation of reaction mechanism is needed.

Author Response: Based on the reviewer's comment, we have paid more attention to the analysis of the peak and plateau at 1.4 V in the cathodic scan (as well as those at 1.9 V in the anodic scan observed in the CV and GCD profiles, respectively. In former times, we have considered that the peak/plateau formed at 1.4 V are associated with SEI formation, considering the decomposition potential (LUMO level) of our electrolyte of ~1.3-1.4 V vs Li^+/Li (*Chem. Mater., 2010, 22, 587-603*). However, based on the critical comment of the reviewer, we concluded that the peak/plateau at 1.4 V and 1.9 V are not associated with SEI formation, but associated with the lithiation and delithiation of nickel sulfide residues with the following reasons.

First, similar peak/plateau at 1.4 V and 1.9V in the CV and GCD profiles were observed from the nickel sulfide anode by other research groups (e.g., *Small, 2013, 9, 8, 1321–1328* & *J. Mater. Chem., 2009, 19, 7277-7283*; see also Fig. R2a,b). Second, the substantially identical behaviors were observed from the nickel-containing samples that were underwent sulfuric acid treatment (Supplementary Fig. S8). Third, sulfur was detected in the GrμT flake by TEM/EDS analysis, which shows that nickel sulfide residues were developed during the nickel etching process using sulfuric acid (Fig. R2c). Thanks to the previous report, we also came to know that the delithiation potential of nickel sulfide shifted towards higher voltage with cycles and exceeded the upper limit of voltage window (2V). This fact is useful to understand the rapid capacity fading that tends to occur in the NiSiNWs@GrμTs anodes in the early stage of cycles.

REDACTED

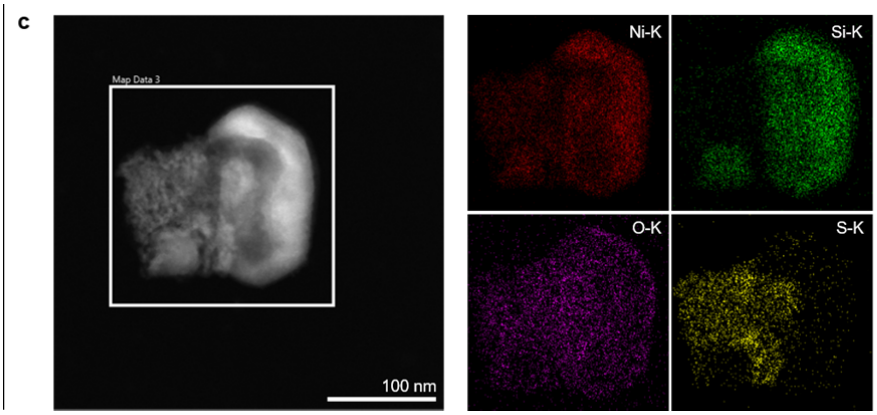

Figure R2. [REDACTED] c, TEM-EDS analysis of a GrμT flake collected by sonication of NiSiNWs@GrμT.

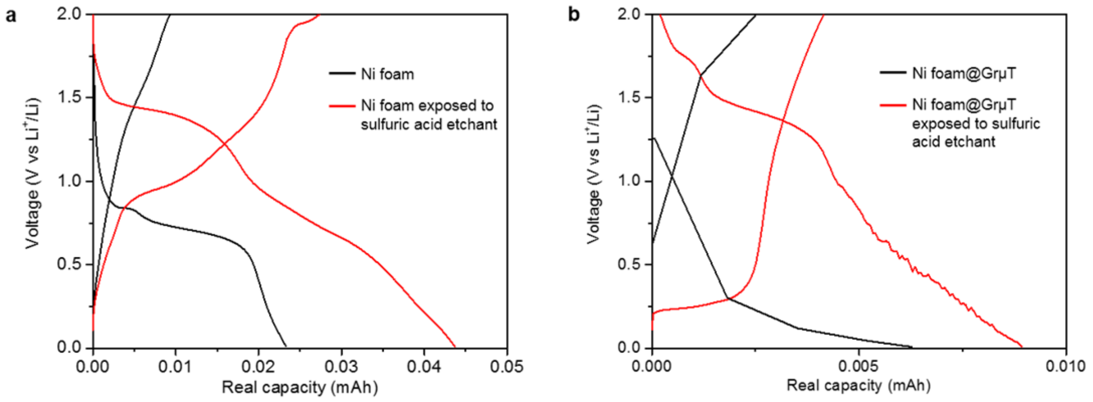

Supplementary Figure S8. Evolution of galvanostatic charge-discharge profiles depending on sulfuric acid treatment. Both the Ni foam (a) and Ni foam@GrμT exhibited new plateaus at 1.4 V and 1.9 V in the galvanostatic charge-discharge (GCD) profiles only after sulfuric acid treatment.

We thank the reviewer for the valuable comments which provides us the opportunity to remedy our wrong analysis. To address this issue, we added Supplementary Fig. S8 to our revised Supplementary Information. In addition, we revised the related paragraph as follows (1st paragraph on page 8) and added the relevant references (ref. 26 & 27):

"In contrast, other peaks at 1.4 V in the cathodic scan and at 1.9 V in the anodic scan diminish gradually during the scans. We can also observe plateaus at 1.4 V and 1.9 V in the galvanostatic charge-discharge (GCD) profiles, displaying similar features in the charging and discharging stages, respectively, and almost disappear after 10-20 cycles (Fig. 3b). The nearly identical behaviors were observed from the nickel sulfide anode[26,27] and the samples that underwent sulfuric acid treatment (Supplementary Fig. S8). Thus, we conclude that the plateaus/peaks at 1.4 V and 1.9 V are associated with the lithiation and delithiation of nickel sulfide residues that were developed during the nickel etching process using sulfuric acid, respectivly. It is noteworthy that the lithiation plateaus disappeared rapidly during the initial several cycles (Fig. 3b). Since the delithiation potential of nickel sulfide shifted toward higher voltage with cycles and exceeded the upper limit of the voltage window (2V)[27], the more lithiated nickel sulfide could not return to a delithiated state with cycles. This mechanism is closely linked to the rapid capacity fading that tends to occur in NiSiNWs@GrμT anodes in the early stage of cycles (see the red and blue plots in Figs. 3 c-e)[28]. Nevertheless, once the anodes were stabilized, there was little change in the charge/discharge profile after 100 cycles at 10C, and the charge/discharge capacities were maintained above 900 $mAhg^{-1}$."

"ref. 26. Lai, C. H. *et al.* Oriented growth of large-scale nickel sulfide nanowire arrays via a general solution route for lithium-ion battery cathode applications. *J. Mater. Chem.* **19**, 7277-7283, doi:10.1039/b909261g (2009)."

&

“ref. 27. Mahmood, N., Zhang, C. Z. & Hou, Y. L. Nickel Sulfide/Nitrogen-Doped Graphene Composites: Phase-Controlled Synthesis and High Performance Anode Materials for Lithium Ion Batteries. *Small* **9**, 1321-1328, doi:10.1002/smll.201203032 (2013).”

Reviewers' comments:

Reviewer #1 (Remarks to the Author):

The authors have added some more data and modified the manuscript. However, the answers to first and fourth question are not convincing.
1. The controlled experiment in supplementary figure 1, the authors used a copper (Cu) bowl coated with a thermally oxidized layer (CuO) on both the outer and inner surfaces, so the KOH inside the bowl had no contact with KOH outside the bowl, it is different in the GrμT structure. In the GrμT structure, the electrolyte inside the PS indeed contacted with the electrolyte outside.
The author replied, "since the GrμT ends are sealed, the electrolytes inside and outside of the GrμT do not directly contact each other".
Firstly, the GrμT ends can not be sealed in the real state; secondly, if electrolytes inside and outside cannot contact, how the Li ion transport to the NiSi NWs and react?
Besides, for the added experiment, the authors still used the AuCl3 solution droplet put on the multilayer graphene which is still separated with the AuCl3 solution contact with the bottom. I think it's different with the electrolyte in the real experiment.

4. I think amounts of Ni will not be removed in the process of Ni etching, and after growth of NiSi nanowries, still some Ni existed inside the graphene, so it can enhance the electron conductivity. Maybe that's the important cause for so good rate performance of this structure. I think it need some experimental data to support the author's result (ie. SEM eds mapping, TEM eds mapping including the graphene part).
The authors just provided electrical resistance of the GrμTs with and without Ni islands, but we know that the real rate performance test is not fully related with the resistance. From the EDS mapping, in some place Ni existed separately with Si and also it's continuously connected.
Therefore, they can not exclude the possibility that the good rate performance is not related with Ni.

Reviewer #2 (Remarks to the Author):

The authors have addressed the major comments of the Reviewers. I recommend publication.

Reviewer #3 (Remarks to the Author):

In the revised manuscript, we authors provided additional experiment to prove his "potential shielding" effect for the NiSi NWs in the electrolyte. However, I still consider the inappropriate comparison of potential gradients developed between these models. FEA simulations can hardly predict the voltage established in the interface between the inner electrolyte and NW. Particularly, CuO/Cu bowl and a GrμT indeed exhibit the different properties as a potential sheath (PS) in that the CuO/Cu bowl is not

ion/solution permeable. Although the authors maintained that the electrochemical reactions (e.g., SEI formation, lithiation, hydrogen evolution, etc.) are governed by a potential gradient, the CuO/Cu bowl experiment is still not comparable to the mechanism of electric potential control to prevent SEI formation on the NW surface since inner KOH solution has not participated in the closed circuit. Similarly, the second experimental setup of the electrochemical cells which consisting of a Pt counter electrode and multilayer graphene membrane demonstrate the similar problem, the potential gradient was not created beneath the graphene membrane only because the complete circuit has been established above the bottom surface of the graphene membrane.

On the other hand, the authors claimed plateaus/peaks at 1.4 V and 1.9 V are associated with the lithiation and delithiation of nickel sulfide residues that were developed during the nickel etching process using sulfuric acid. But no XRD data is provided to prove the formation of this nickel sulfide phase. And I suggest the authors change the treatment procedures of the samples to remove these residues.

Reviewer #1 (Remarks to the Author):
The authors have added some more data and modified the manuscript. However, the answers to first and fourth question are not convincing.

Comment 1: The controlled experiment in supplementary figure 1, the authors used a copper (Cu) bowl coated with a thermally oxidized layer (CuO) on both the outer and inner surfaces, so the KOH inside the bowl had no contact with KOH outside the bowl, it is different in the GrμT structure. In the GrμT structure, the electrolyte inside the PS indeed contacted with the electrolyte outside. The author replied, "since the GrμT ends are sealed, the electrolytes inside and outside of the GrμT do not directly contact each other". Firstly, the GrμT ends cannot be sealed in the real state; secondly, if electrolytes inside and outside cannot contact, how the Li ion transport to the NiSi NWs and react? Besides, for the added experiment, the authors still used the AuCl3 solution droplet put on the multilayer graphene which is still separated with the AuCl3 solution contact with the bottom. I think it's different with the electrolyte in the real experiment.

Author Response:

We apologize for our insufficient explanation causing the reviewer to be confused. We believe that the 3 major issues (1. Sealing of the GrμT ends; 2. Ion/molecule-exchange through GrμT PS vs. Ion conduction in bulk electrolyte; 3. Li transport from the outer electrolyte to the NiSi NWs inside the GrμT) should be worth discussing. Our responses to these concerns are below.

1. Sealing of the GrμT ends

Since the NiSiNWs@GrμT is fabricated with a coin-shaped Ni foam from the beginning (Fig. R1a), the Ni foam ends are indeed covered by multi-layer graphene after CVD process. Accordingly, the GrμT ends remain sealed, as confirmed by SEM images (Fig. R1b). Therefore, in our cells, the electrolytes inside and outside are physically separated by the GrμT and thus do not directly contact each other.

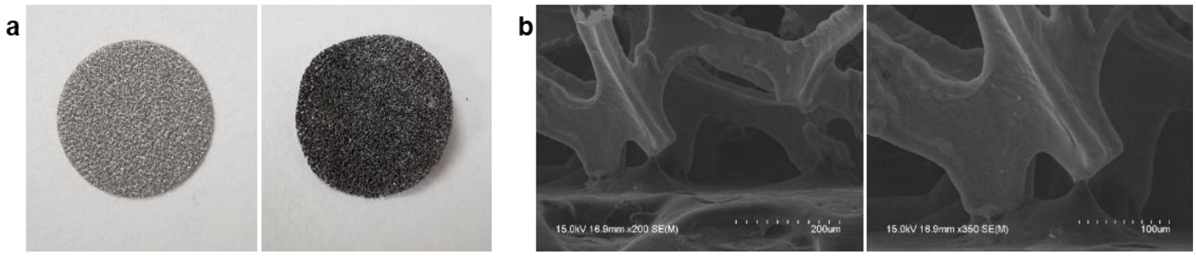

Figure R1. a, Photographs of coin-shaped Ni foam (left panel) and NiSiNWs@GrμT electrode (right panel). **b,** SEM images of NiSiNWs@GrμT show that the GrμT ends remain sealed.

2. Ion/molecule-exchange through GrμT PS vs. Ion conduction in bulk electrolyte

Meanwhile, given the permeation of etchant and gaseous source through the multi-layer graphene walls (GrμT PS) to produce NiSiNWs@GrμT, we conclude that ion/molecule-permeation occurs in GrμT PS, especially via the atomic-scale defects.

However, the rate of ion/mass permeation through the GrμT PS is very slow with regards to the ionic conduction in the electrolytes. Accordingly, although the CuO bowl and GrμT PS have large differences in the ion exchange rate, we can assume that both systems are similar in that the inner/external electrolytes are electrically separated.

In addition, in the case of Controlled experiment II, the $AuCl_3$ droplet and solution are separated by multilayer graphene sheet, and are not in direct contact with each other. Accordingly, both the GrμT PS and multilayer graphene sheet are basically very similar in the ion/mass exchange. In the Controlled experiment II, the permeation of ions/molecules to some extend through the multilayer graphene PS was further confirmed by the observation that the $AuCl_3$ droplet gradually percolated down through multilayer graphene sheet and became smaller (Fig. R2; see also Supplementary Video S2).

In this situation, electrochemical reaction (i.e., electrodeposition of Au) occurred only on the bottom surface of the multilayer graphene (Supplementary Figure S3f). We believe that this phenomenon is exactly comparable to our NiSiNWs@GrμT case (i.e., SEI formation & lithiation only at the outer electrolyte and GrμT interface).

Overall, these considerations indicate that the Controlled experiments I and II are useful/suitable to demonstrate our hypothesis.

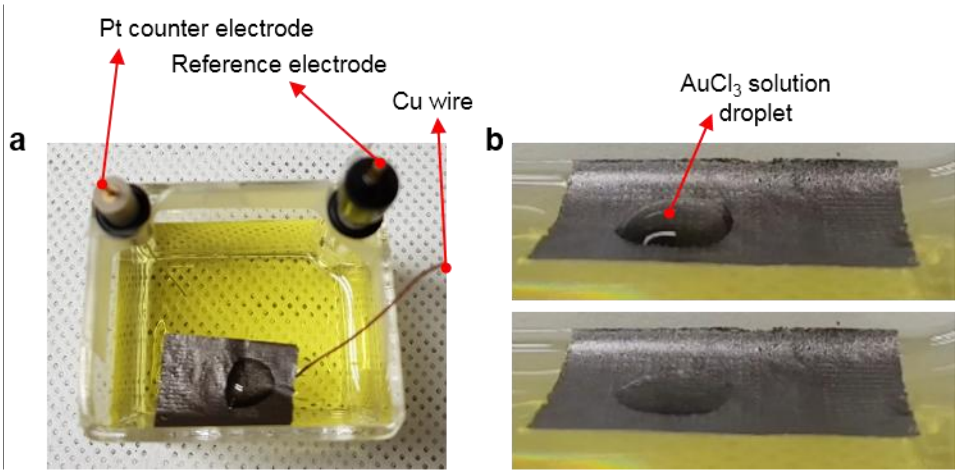

Figure R2, a, Photograph of the Controlled experiment with a multilayer graphene on which a droplet of $AuCl_3$ aqueous electrolyte was placed. **b,** Photograph of $AuCl_3$ droplet placed on multilayer graphene, taken at an early stage (upper panel) and after 70 s electrodeposition (bottom panel).

3. Li transport from the outer electrolyte to the NiSiNWs inside the GrμT.

To answer the second question (if electrolytes inside and outside cannot contact, how the Li ion transport to the NiSi NWs and react?), we have schematically illustrated the lithiation process of the NiSiNWs@GrμT (Fig. R3a). As the reviewer pointed out, there is no potential

gradient at the internal electrolyte and NW interface to induce the SEI formation and lithiation. Instead, the SEI formation and lithiation reactions take place at the external electrolyte and PS interface where a potential gradient is concentrated. Thereafter, the intercalated Li at the PS diffuses to the NWs anchored to the inner surface of the PS (Fig. R3a). Similar behaviors are observed in many previous studies (e.g., *Nano Lett., 12, 3315, 2012* and *Nat. Nanotech., 9, 187, 2014*, in which lithiations of active materials inside carbon-shell takes place at the interface between external electrolyte and the carbon-shell, Fig. R3b).

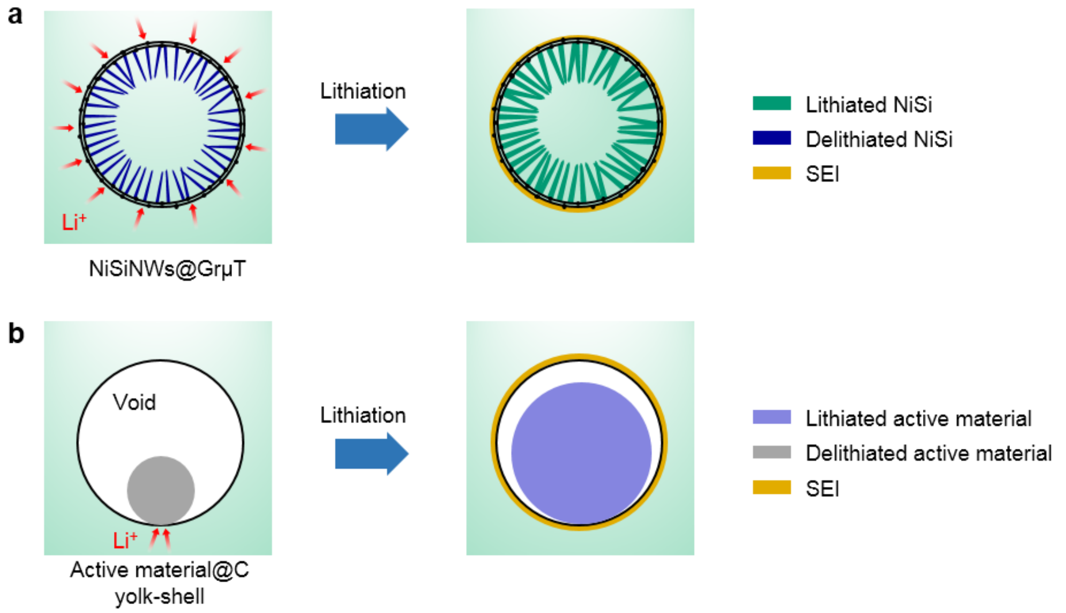

Figure R3. a,b, Schematics of lithiation mechanism in the case of NiSiNWs@GrμT (a) and active material@C yolk-shell (b), respectively.

Meanwhile, in the NiSiNWs@GrμT, the long diffusion path for lithiation of NWs along axial direction would have a disadvantage in ultrafast charge/discharge. However, as discussed in our manuscript (this issue was already discussed in previous revision, see the response to Reviewer #2’s 3rd comment), previous in-situ TEM studies demonstrated the fast lithiation kinetics in individual NWs. FEA simulation for this case shows that the potential gradient is concentrated at the interface between tip of the NW and electrolyte (Li_2O), as similar to case of NiSiNWs@GrμT. In this situation, Li insertion occurred at one tip of the NWs and followed by Li transport along axial direction with axial speed up to ~213 nm/s for highly-conducting SiNWs (ref 31, Fig. R4a,b).

REDACTED

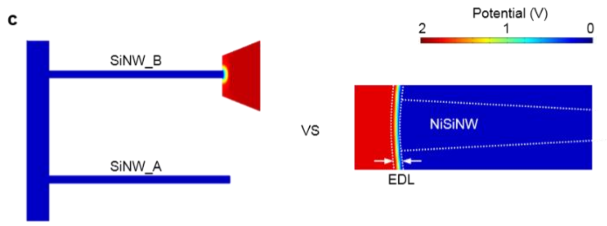

Figure R4. [REDACTED] c, FEA simulation for the case of (a) shows that the potential gradient is concentrated at the NW tip/electrolyte interface as similar to the case of our NiSiNWs@GrμT.

To address these issues, we added Fig. R1 and Fig. R2 to our revised Supplementary Fig. S3 and Supplementary Fig. S5, respectively. In addition, we added following sentences to the revised manuscript:

"However, the rate of ion permeation through the GrμT is very slow with regards to the ionic conduction in the electrolyte so that the inner and external electrolytes would be electrically separated." (1st paragraph on page 6)

"Since the NiSiNWs@GrμT is fabricated with a coin-shaped Ni foam from the beginning, the GrμT ends remain sealed, as confirmed by SEM images (Supplementary Fig. S5e,f)." (2nd paragraph on page 7)

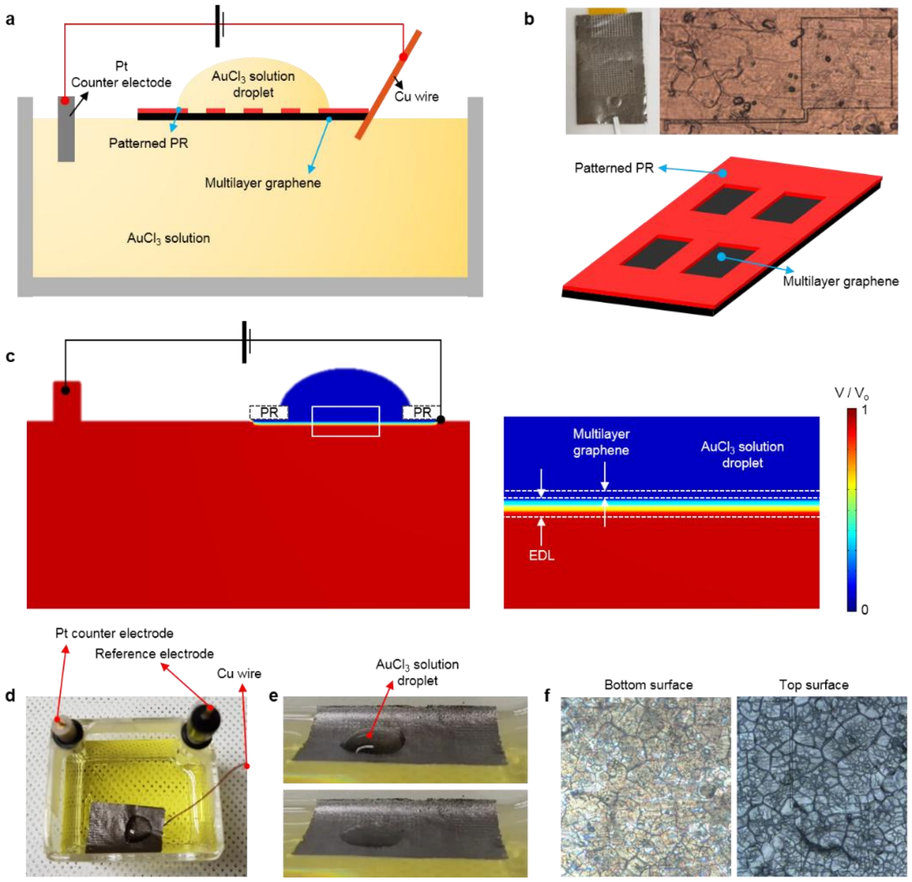

Supplementary Figure S3. Controlled experiment (II) illustrating electric potential control of the electrodeposition reaction. a, Schematic of the experimental setup of the electrochemical cells, consisting of a Pt counter electrode and multilayer graphene membrane connecting with Cu working electrode; the graphene membrane is physically supported by a patterned photo-resist (PR) layer; electrodes are exposed to a 1.0 M $AuCl_3$ aqueous electrolyte, and a droplet of the same electrolyte is placed on the graphene membrane. An external load drives the Au-deposition reaction on the graphene membrane surface. **b,** Photographs (top) and schematic of graphene membrane. **c,** FEA simulation of potential distribution across the cell. Bottom panel: enlarged image taken from the square in the left panel showing that the potential gradient is concentrated on the bottom surface of the graphene membrane. **d,** Photograph of the controlled experiment with a multilayer graphene on which a droplet of $AuCl_3$ aqueous electrolyte was placed. **e,** Photograph of $AuCl_3$ droplet placed on multilayer graphene, taken at an early stage (upper panel) and after 70 s electrodeposition (bottom panel). The $AuCl_3$ droplet gradually percolated down through multilayer graphene sheet and became smaller, illustrating the permeation of ions/molecules to some extend through the multilayer graphene PS (See also Supplementary Video S2). **f,** Optical-microscope images of bottom (left) and top (right) surfaces of the graphene membrane after Au-electrodeposition with an external bias of -1.8 V vs. SHE for 100 s, showing that Au-deposition occurred only on the bottom surface of the graphene membrane. Despite the permeation of ions/molecules (but it is extremely slow with regards to the ionic conduction in the electrolytes), Au electrodeposition reaction occurred only on the bottom surface of the multilayer graphene. This result indicates that the $AuCl_3$ droplet is electrically separated from the $AuCl_3$ solution by the multilayer graphene. This control experiment also demonstrates the validity of our approach.

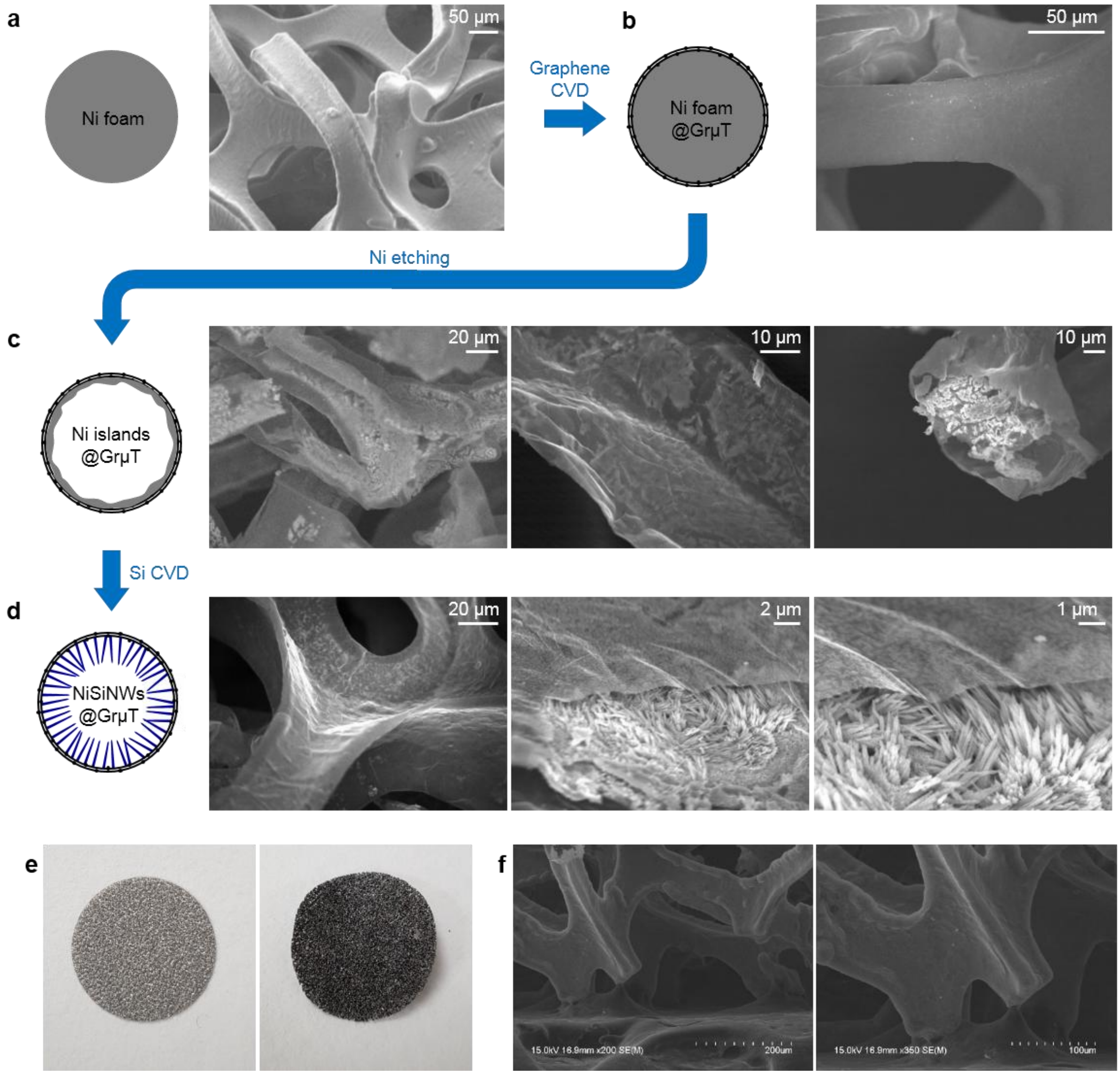

Supplementary Figure S5. Schematics and SEM images at each preparation step of NiSiNWs@GrµT. a-d, Ni foam (**a**), graphene-on-Ni foam (**b**), Ni islands@GrµT (**c**) and NiSiNWs@GrµTs (**d**). **e,** Photographs of coin-shaped Ni foam (left panel) and NiSiNWs@GrµT electrode (right panel). **f,** SEM images of NiSiNWs@GrµT show that the GrµT ends remain sealed.

Comment 4: I think amounts of Ni will not be removed in the process of Ni etching, and after growth of NiSi nanowries, still some Ni existed inside the graphene, so it can enhance the electron conductivity. Maybe that's the important cause for so good rate performance of this structure. I think it need some experimental data to support the author's result (ie. SEM eds mapping, TEM eds mapping including the graphene part).The authors just provided electrical resistance of the GrμTs with and without Ni islands, but we know that the real rate performance test is not fully related with the resistance. From the EDS mapping, in some place Ni existed separately with Si and also it's continuously connected. Therefore, they cannot exclude the possibility that the good rate performance is not related with Ni.

Author Response:

As the reviewer suggested, we have conducted a detailed TEM/EDS element analysis of fragments of the NiSiNWs@GrμT sample. The fragments were prepared by sonicating the NiSiNWs@GrμT sample. During the sonication, most NiSiNWs were detached from the fragments and only GrμT flakes coated with thin island layers remained, which makes the TEM/EDS analysis easier (Supplementary Fig. S9a). From the structural and element analysis, we could confirm that those islands are amorphous SiO_X, where 5-20 nm-diameter Ni nanoparticles are embedded/dispersed (Supplementary Fig. S9e). We believe that during the CVD process for the NiSiNW growth, thin Si island layers also developed on the surface of Ni islands and at the same time Ni-residue, which is not participated in NiSiNWs growth, would form nanoparicles inside/around the Si islands that were thereafter oxidized to form amorphous SiO_X islands.

Besides, as the reviewer commented, we cannot exclude the contribution of Ni nanoparticles to the improved rate performance. However, our in-depth analysis and consideration illustrate that the "potential shielding" effect plays major role in achieving high rate (80C) capability. As the reason for this assertion, we considered the NiSiNWs-on-NiF anode that despite of excellent electric conduction by the Ni beneath NiSiNWs, cannot withstand high-rate cycling exceeding 10C.

To response the reviewer's comment, we added Supplementary Fig. S9 to our revised Supplementary Information. In addition, we revised the related paragraphs as follows;

"From the TEM images of the fragment separated from the NiSiNWs@GrμT, we found that thin island layers are remained on the inside surface of the GrμT (Supplementary Fig. S9). The EDS analysis confirmed that those islands are amorphous SiO_X embedded with 5-20 nm-diameter Ni nanoparticles." (2nd paragraph on page 7)

"Although we cannot exclude the contribution of residual Ni nanoparticles (Supplementary Fig. S9) to the improved rate performance, our in-depth analysis and consideration illustrate that the "potential shielding" effect plays major role in achieving high rate capability." (1st paragraph on page 11)

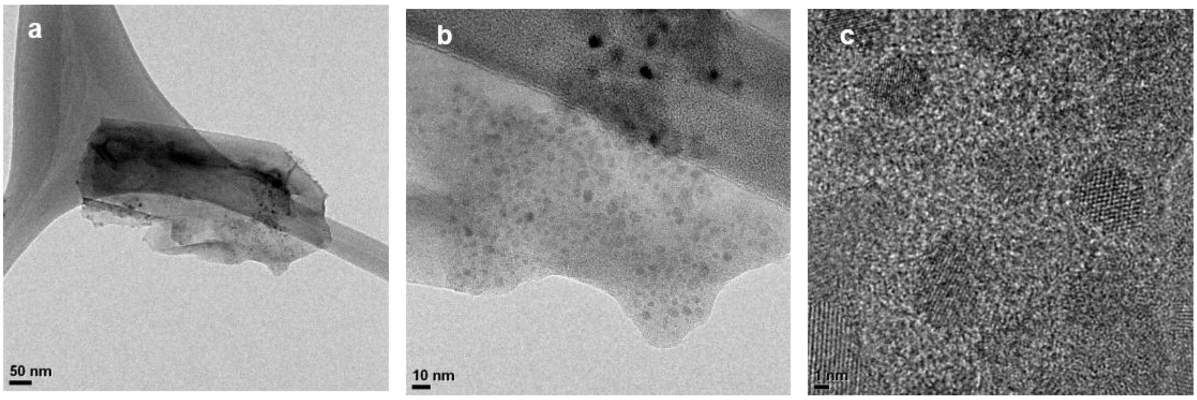

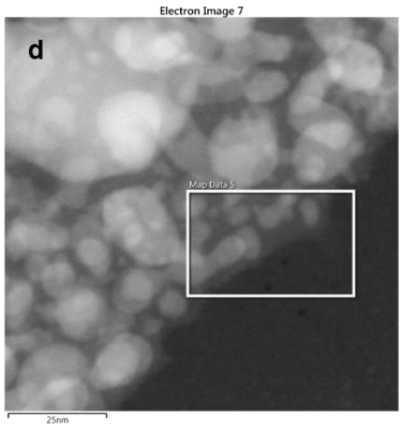

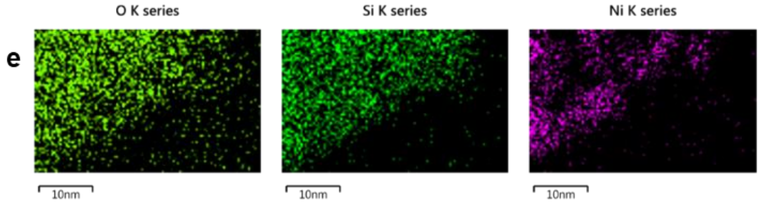

Element	Wt. %	At. %
C	63.03	78.56
O	13.00	12.16
Si	11.40	6.08
Ni	12.56	3.20
Total:	100.00	100.00

Supplementary Figure S9. TEM-EDS analyses of GrμT fragment separated from the NiSiNWs@GrμT. a-c, TEM images in low (a) and high (b,c) magnification of GrμT fragment placed on a lacey carbon film supported on a copper grid. The fragments were prepared by sonicating the NiSiNWs@GrμT sample. During the sonication, most NiSiNWs were detached from the fragments and only GrμT flakes coated with thin island layers remained. The high magnification images show that 5-20 nm-diameter Ni nanoparticles are embedded in amorphous islands. **d,e,** TEM-EDS element analysis of GrμT flake taken over the area inside the square in dark-field image (d). From the elemental mapping images (upper panels in e) and summaries of elemental composition (bottom table in e), the islands are determined to be amorphous SiO_X. This analysis illustrates that during the CVD NiSiNW growth, thin Si island layers also developed on the surface of Ni islands and at the same time Ni-residue, which is not participated in NiSiNWs growth, would form nanoparticles inside/around the Si islands that were thereafter oxidized to form amorphous SiO_X islands.

Reviewer #3 (Remarks to the Author):

Comment 1: In the revised manuscript, authors provided additional experiment to prove his "potential shielding" effect for the NiSi NWs in the electrolyte. However, I still consider the inappropriate comparison of potential gradients developed between these models. (i) FEA simulations can hardly predict the voltage established in the interface between the inner electrolyte and NW. (ii) Particularly, CuO/Cu bowl and a GrμT indeed exhibit the different properties as a potential sheath (PS) in that the CuO/Cu bowl is not ion/solution permeable. (iii) Although the authors maintained that the electrochemical reactions (e.g., SEI formation, lithiation, hydrogen evolution, etc.) are governed by a potential gradient, the CuO/Cu bowl experiment is still not comparable to the mechanism of electric potential control to prevent SEI formation on the NW surface since inner KOH solution has not participated in the closed circuit. Similarly, the second experimental setup of the electrochemical cells which consisting of a Pt counter electrode and multilayer graphene membrane demonstrate the similar problem, the potential gradient was not created beneath the graphene membrane only because the complete circuit has been established above the bottom surface of the graphene membrane.

Author Response:

We thank the reviewer for the valuable comments which provides us the opportunity to compensate an incomplete explanation and remedy the mistake. To responses to the concerns raised in this comment, the following 3 major issues will be discussed: 1. 'Potential shielding effect' in different geometries; 2. Ion/molecule-exchange through GrμT PS vs. Ion conduction in bulk electrolyte; 3. Contact potentials at the electrolyte and anode interface.

1. 'Potential shielding effect' in different geometries

This issue is related to the reviewer's third concern that inner and upper electrolytes in the Controlled experiments (I) and (II), respectively, do not participated in the "electric closed circuit" (or do not form a complete loop with external electric load) and thus they are not appropriate to demonstrate the "potential shielding effect" of our NiSiNWs@GrμT cell.

We respectively disagree with this reviewer's argument in that electrolyte inside the GrμT are electrically isolated from the outer electrolyte (that he referred to as the 'external electric closed circuit') by the GrμT PS, since the all GrμT surface (including tube ends) is completely sealed (see the response to Reviewer #1's 1st comment). In other words, the inner electrolyte of the NiSiNWs@GrμT cell also does not participated in the 'electric closed circuit' owing to the 'potential shielding effect' of the GrμT, as similar to the Controlled experiments (I) & (II).

To make this point clearer, we have compared the voltage distributions across the electrochemical cells of three different geometries, while assuming that the thickness and material of the PSs (i.e., tube wall, bowl, and floating membrane) are identical and also the same electrolyte (Fig. R5a). In addition, to elucidate the effect of ion/molecule permeation to the potential shielding effect, we have introduced different sized holes in the PSs, and simulated the change of the potential distribution across the cells (Fig. R5b,c).

These simulation results showed that despite difference in the geometries the three cells have similar characteristics in that (i) potential gradients concentrate mainly at the outer/lower electrolytes and PS interfaces for $R_h \leq T_{EDL}$ (Fig. R5b) and (ii) if $R_h >> T_{EDL}$ (Fig. R5c), significant ion transport occurs thorough the holes and the degree of the 'potential shielding

effect' decreases rapidly, producing potential gradients even at the inner/upper electrolytes and PS interfaces.

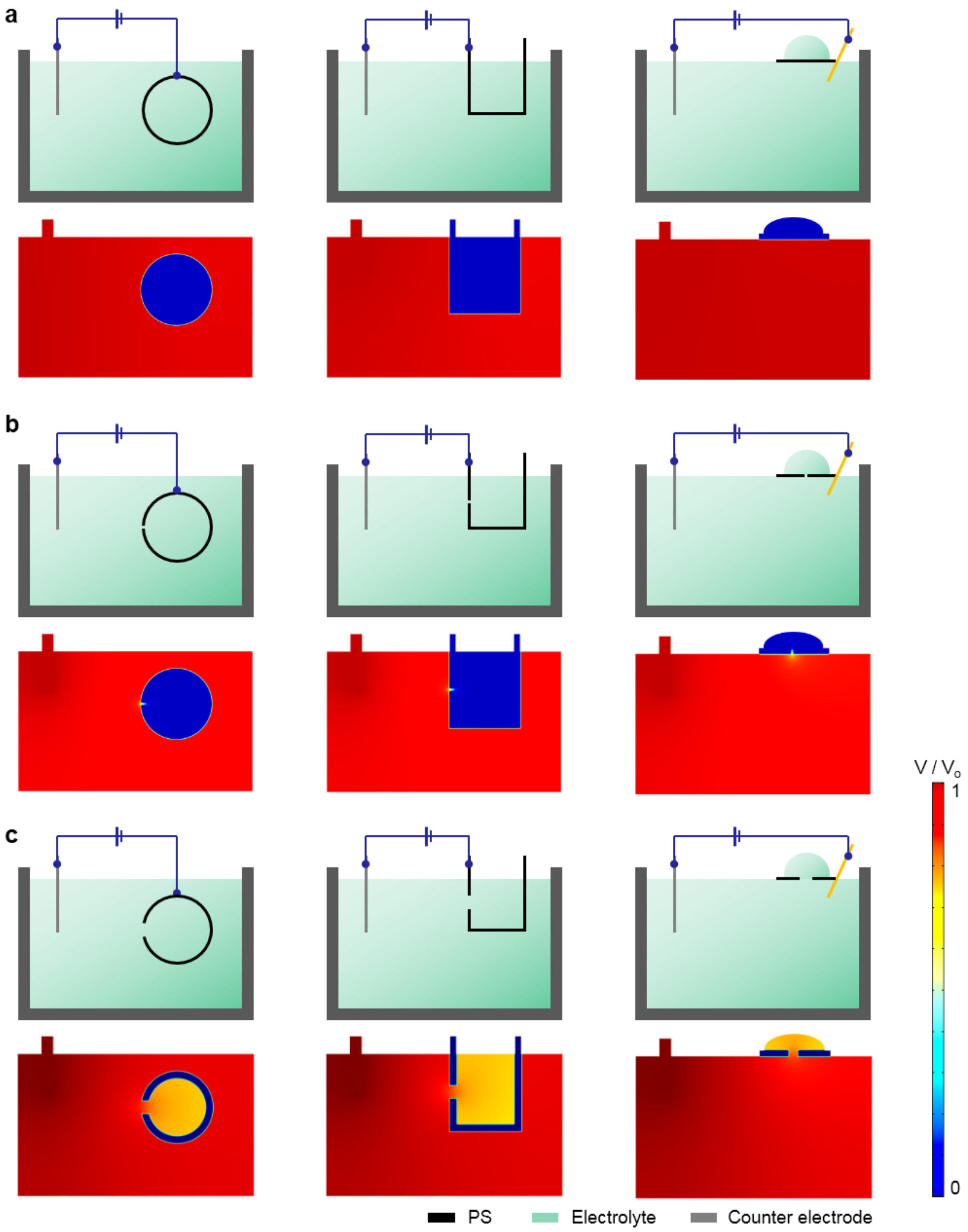

Figure R5. a,b,c, Schematics of electrochemical cells, which have identical electrolyte and PS, in different geometry. (the hole sizes are characterized by the correlation between the radii of holes (R_h) and EDL thickness (T_{EDL}): Fig. R5b, $R_h \approx T_{EDL}$; **c**, $R_h \approx 3T_{EDL}$).

2. Ion/molecule-exchange through GrμT PS vs. Ion conduction in bulk electrolyte

Meanwhile, as the reviewer pointed out, the CuO/Cu bowl and GrμT indeed exhibit the different properties as a potential sheath (PS) given that the CuO/Cu bowl is almost ion/solution permeable (Comment 1-(ii)). Nonetheless, it should be noted that the permeation of ion/mass through the GrμT is very slow with respect to the ionic conduction in the electrolyte. We therefore consider that despite the different ion/solution permeabilities between the CuO/Cu bowl and GrμT, both systems are similar in that the inner/external electrolyte are electrically separated.

In addition, in the case of Controlled experiment II, the $AuCl_3$ droplet and solution are separated by multilayer graphene sheet, and are not in direct contact with each other. Accordingly, both the GrμT PS and multilayer graphene sheet are basically very similar in the ion/mass exchange. In the Controlled experiment II, the permeation of ions/molecules to some extend through the multilayer graphene PS was further confirmed by the observation that the $AuCl_3$ droplet gradually percolated down through multilayer graphene sheet and became smaller (Fig. R6; see also Supplementary Video S2).

In this situation, electrochemical reaction (i.e., electrodeposition of Au) occurred only on the bottom surface of the multilayer graphene (Supplementary Figure S3f). We believe that this phenomenon is exactly comparable to our NiSiNWs@GrμT case (i.e., SEI formation & lithiation only at the outer electrolyte and GrμT interface).

Overall, these considerations indicate that the Controlled experiments I and II are useful/suitable to demonstrate the validity of our argument (i.e., the SEI formation and Li insertion occur only on the external electrolyte/GrμT interface where the potential gradient is created).

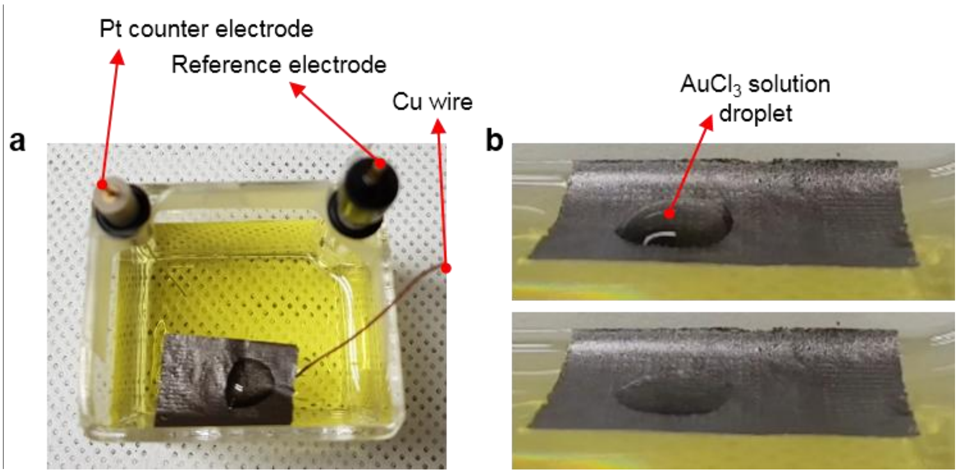

Figure R6, a, Photograph of the Controlled experiment with a multilayer graphene on which a droplet of $AuCl_3$ aqueous electrolyte was placed. **b,** Photograph of $AuCl_3$ droplet placed on multilayer graphene, taken at an early stage (upper panel) and after 70 s electrodeposition (bottom panel).

3. Fermi level in electrolytes: electrochemical potentials at electrolyte-electrode interfaces

We agree with reviewer's comment that our FEA simulations cannot predict the voltage established in the interface between the inner electrolyte and NW (Comment 1-(i)). In this simulation, we considered a contact potential difference in the interface between the inner electrolyte and the NW and imposed an arbitrary value (~0.2 V) of potential drop equivalent to the contact potential.

However, the potential difference at the interface causes the redistribution of charged carriers until the Fermi levels in the both phases are equal. Accordingly, in thermodynamic equilibrium, voltage difference (corresponding to the difference in Fermi level or chemical potential for electrons in energy scale) does not established over the electrode and electrolyte interface (Fig. R7). We therefore conclude that there is no voltage difference at the electrolyte and electrode interface if the two phases are in thermodynamic equilibrium with each other. In accordance with this consideration, since we performed the FEA simulations to predict the voltage distributions in the LIB cells, it is not appropriate to impose intentionally the potential drop across the inner electrolyte and NW anode.

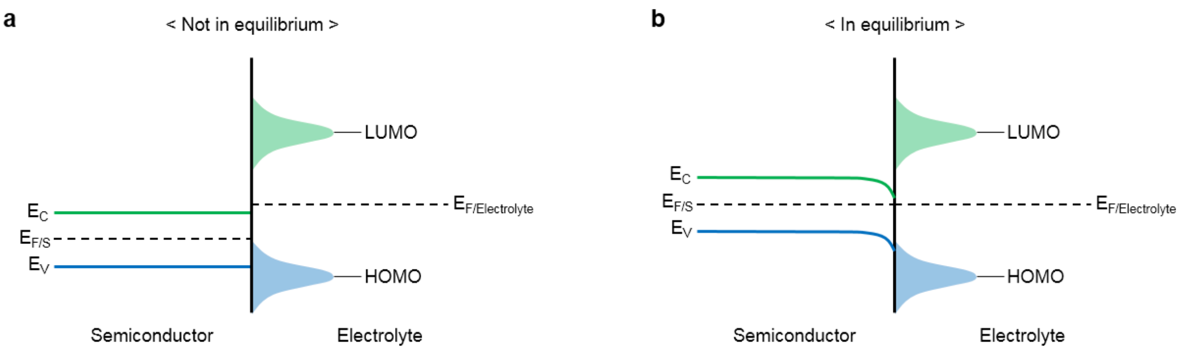

Figure R7. a,b, Electron energy diagrams of solid/electrolyte interface not in equilibrium (a) and in equilibrium (b). In thermodynamic equilibrium state, the 'chemical potential (Fermi level)' of each component should be equal.

In addition, considering the open-circuit voltage (V_{oc}) of ~2 V across the half-cells, we simply assumed that an external load of 2 V was applied between the left-hand side electrolyte counter electrode and GrμT. This assumption is useful to simply the FEA simulation, but in a two-electrode cell it is hard to measure/define the real value of the V_E. In this revision, an external potential bias of V_o was applied between left-hand side counter electrode and GrμT, and then position-dependent potential values across the electrolyte and anode were calculated. Then these potential values were converted to relative values with respect to V_o (i.e., normalized to V/V_o), which make our simulation result more general. The revised FEA simulation results are found in Fig. 1c,d, Fig. 2b, Supplementary Fig. S2b,c, Supplementary Fig. S3c, Supplementary Fig. S4. We thank the reviewer for bringing out this issue to our attention; we therefore can have opportunity to remedy the fault and to achieve more exquisite analysis.

Meanwhile, as like our case, the relationship between the concepts/pictures of solid-state-physics and electrochemistry can lead to some confusion. Especially, the opposite sign in the potential diagram and energy diagram frequently cause confusion. We therefore provided the schematic potential and energy diagram across a conventional battery cell, together with the

correlation between potentials of electrode and electrolyte in the electrochemical scale and their own Fermi levels in the energy scale (see Supplementary Fig. S1 in out revised Supplementary information). We believe that a graphical presentation of the potential profiles across the cell accompanying charging cycle would be useful to illustrate our strategy based on controlling electric potential.

To respond to the reviewer's comment, we revised the manuscript, as follows:

1. We added Supplementary Fig. S1 and revised Supplementary Fig. S3 in our Supplementary Information.

2. We replaced the following figures with newly updated FEA simulation results:

   Fig. 1c,d, Fig. 2b, Supplementary Fig. S2b,c, Supplementary Fig. S3c, Supplementary Fig. S4. 3.

3. We revised the related paragraph in the manuscript, as follows:

   "a potential gradient" was replaced with "a potential difference or gradient" (1st paragraph on page 4).

   "In such a scenario, the enclosed space is potentially isolated, in which the potentials of the electrode and electrolyte remain equal (i.e. $V_A = V_E^*$ in thermodynamic equilibrium) regardless of the external bias (left panel in Fig. 1d)[17]. Given that the SEI formation potential across the inner electrolyte and anode interface (V_{SEI}^*), is determined with respect to the V_E^* (or LUMO is determined with respect to the Fermi level in electrolyte), the V_{SEI}^* remains lower than V_A (middle and right panels in Fig. 1d). This consideration illustrates that even at a low working potential of the anode ($V_A < V_{Lith}$), SEI formation on the surface of the NW anode is substantially suppressed. Control experiments support this assertion (Supplementary Fig. S2 and S3)." (1st paragraph on page 5, revised)

   "However, the rate of ion permeation through the GrμT is very slow with regards to the ionic conduction in the electrolyte so that the inner and external electrolytes would be electrically separated." (1st paragraph on page 6, added)

4. We added new references in the revised manuscript and our Supplementary Information.

   "ref 17. Bignozzi, C. A. *et al.* Nanostructured photoelectrodes based on WO3: applications to photooxidation of aqueous electrolytes. *Chem. Soc. Rev.* **42**, 2228-2246, doi:10.1039/c2cs35373c (2013)."

   "ref S1. Bisquert, J., Cendula, P., Bertoluzzi, L. & Gimenez, S. Energy Diagram of Semiconductor/Electrolyte Junctions. *J. Phys. Chem. Lett.* **5**, 205-207, doi:10.1021/jz402703d (2014)."

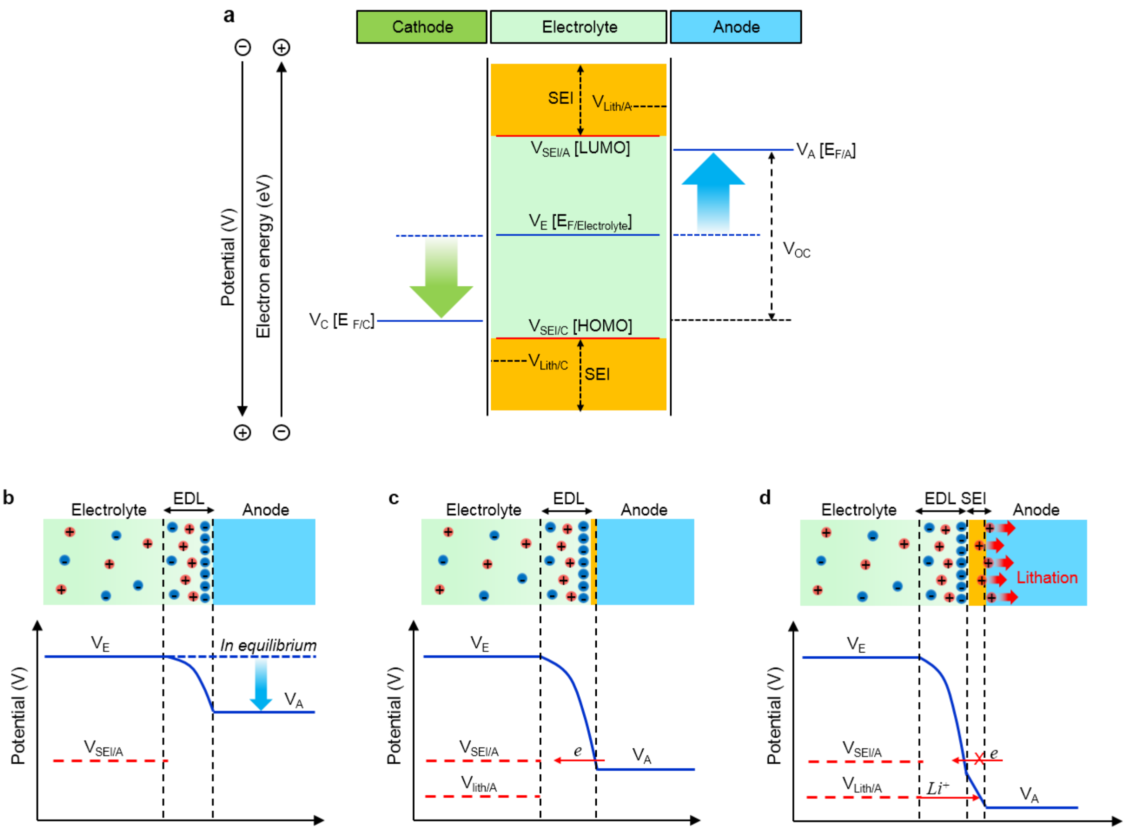

Supplementary Figure S1. Potential/energy diagram across a battery cell. **a,** Schematic potential and energy diagram across a lithium ion battery cell, consisting of cathode, electrolyte and anode. Potentials of cathode (V_C), electrolyte (V_E) and anode (V_A) correspond to their own Fermi levels (or electrochemical potential of electrons) of $E_{F/C}$, $E_{F/Electolyte}$, and $E_{F/A}$, respectively, in the energy scale[S1]. The $V_{Lith/C}$ and $V_{Lith/A}$ refer to (de)lithiation potential of cathode and anode, respectively. During the charging, the V_C and V_A move in the opposite direction, establishing open circuit voltage (V_{OC}). In a cathode, SEI formation starts at a potential V_C above the $V_{SEI/C}$ (or the $E_{F/C}$ lies below the HOMO); in an anode, SEI formation start at a V_A below the $V_{SEI/A}$ (or the $E_{F/A}$ lies above the LUMO). Similarly, delithiation starts at the V_C above the $V_{Lith/C}$ in the cathode while lithiation starts at the V_A below the $V_{Lith/A}$ in the anode side. **b-d,** Schematic images illustrating the developments of a EDL and SEI layer and corresponding potential profiles across the electrolyte and anode, during the first charging cycle. During the charging, the V_A move downward, establishing rapid potential gradient across very thin EDL (b). When the V_A lies below the $V_{SEI/A}$, the electrolyte will be reduced near the anode surface and a thin passivation layer of SEI will be developed on the anode surface until the SEI layer prevents electron transfer from the anode to the electrolyte LUMO (c). At a potential V_A below the $V_{Lith/A}$ regular lithiation involving transport of Li^+ to the anode surface through ion-permeable SEI and thereafter reduction and insertion into the anode.

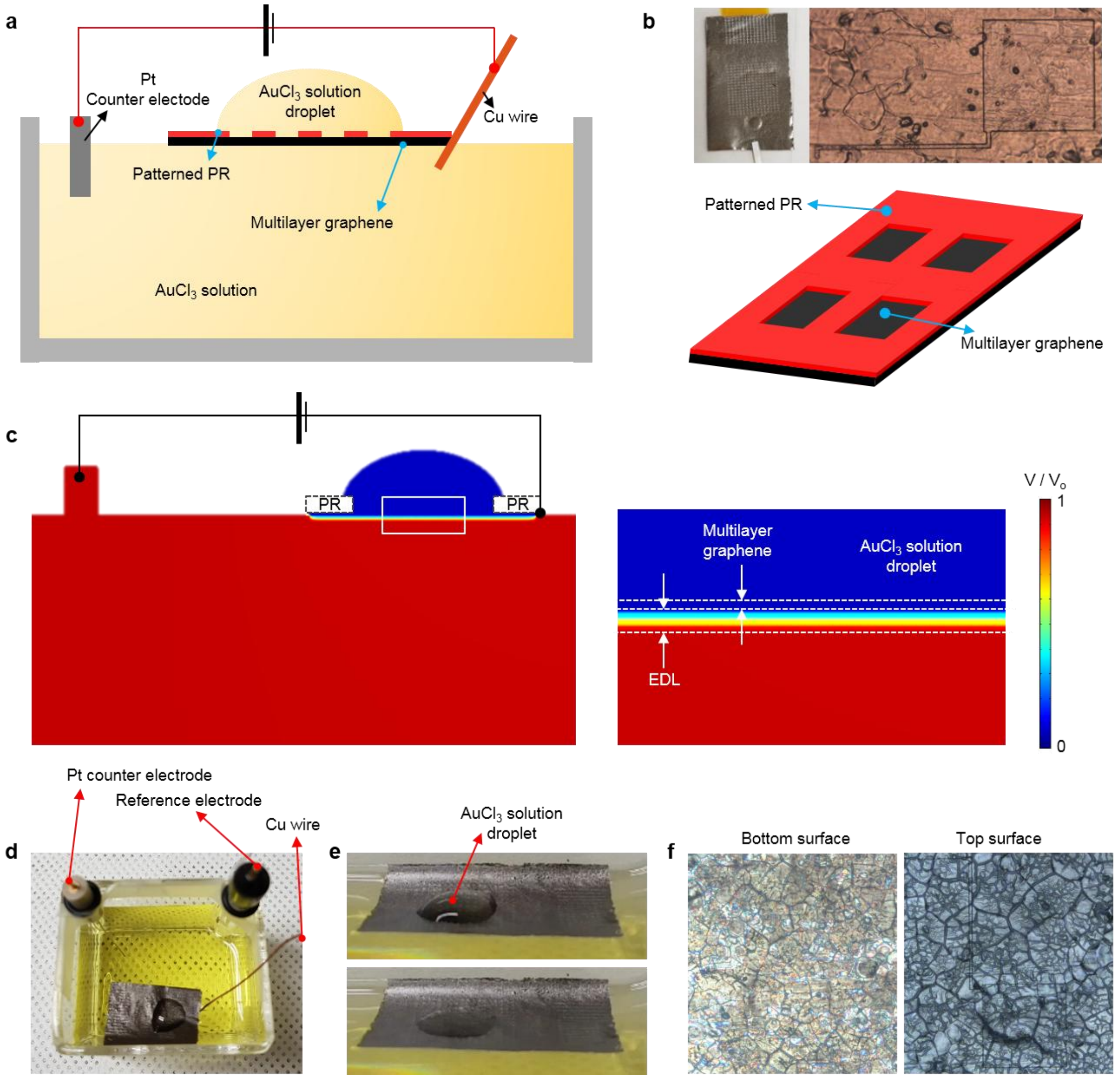

Supplementary Figure S3. Controlled experiment (II) illustrating electric potential control of the electrodeposition reaction. a, Schematic of the experimental setup of the electrochemical cells, consisting of a Pt counter electrode and multilayer graphene membrane connecting with Cu working electrode; the graphene membrane is physically supported by a patterned photo-resist (PR) layer; electrodes are exposed to a 1.0 M $AuCl_3$ aqueous electrolyte, and a droplet of the same electrolyte is placed on the graphene membrane. An external load drives the Au-deposition reaction on the graphene membrane surface. **b,** Photographs (top) and schematic of graphene membrane. **c,** FEA simulation of potential distribution across the cell. Bottom panel: enlarged image taken from the square in the left panel showing that the potential gradient is concentrated on the bottom surface of the graphene membrane. **d,** Photograph of the controlled experiment with a multilayer graphene on which a droplet of $AuCl_3$ aqueous electrolyte was placed. **e,** Photograph of $AuCl_3$ droplet placed on multilayer graphene, taken at an early stage (upper panel) and after 70 s electrodeposition (bottom panel). The $AuCl_3$ droplet gradually percolated down through multilayer graphene sheet and became smaller, illustrating the permeation of ions/molecules to some extend through the multilayer graphene PS (See also Supplementary Video S2). **f,** Optical-microscope images of bottom (left) and top (right) surfaces of the graphene membrane after Au-electrodeposition with an external bias of -1.8 V

vs. SHE for 100 s, showing that Au-deposition occurred only on the bottom surface of the graphene membrane. Despite the permeation of ions/molecules (but it is extremely slow with regards to the ionic conduction in the electrolytes), Au electrodeposition reaction occurred only on the bottom surface of the multilayer graphene. This result indicates that the $AuCl_3$ droplet is electrically separated from the $AuCl_3$ solution by the multilayer graphene. This control experiment also demonstrates the validity of our approach.

Comment 2: On the other hand, the authors claimed plateaus/peaks at 1.4 V and 1.9 V are associated with the lithiation and delithiation of nickel sulfide residues that were developed during the nickel etching process using sulfuric acid. But no XRD data is provided to prove the formation of this nickel sulfide phase. And I suggest the authors change the treatment procedures of the samples to remove these residues.

Author Response:

According to the reviewer's suggestion, we have performed XRD θ-2θ measurement of NiSiNWs@GrμT and Ni islands@GrμT (Supplementary Figure S8c), but could not find the distinct diffraction peaks of nickel sulfide. We attribute this to the small amount of nickel sulfide and its poor crystallization, together with disturbance by broad background signal from the GrμT (20˚- 35˚ degree from multilayer graphene wall). On the other hand, in Supplementary Fig. S12c, when we have conducted the XRD measurement of Ni foam after sulfuric acid treatment for 10 min, we could find weak but clear peaks at 2θ values of 28.22˚ and 31.67˚, corresponding to orthorhombic Ni_9S_8 (*Dalton Trans., 2010, 39, 6080–6091*). In addition, the color of the sample turned into black (Supplementary Fig. S12a and b), which is known to the color of nickel sulfide (*Chem. Mater., 13, 3, 2001*). To further confirm the formation of nickel sulfide phase, we have performed the SEM-EDS and X-ray photoelectron spectroscopy (XPS) measurements of Ni foam after SAT. Both analyses confirm the existence of S element (about 2 at.%). In addition, from the XPS spectra, we observed the Ni $2p_{1/2}$ and $2p_{3/2}$ peaks associated with $Ni^{2+}$and Ni^{3+}, and the S $2p_{1/2}$ and $2p_{3/2}$ peaks associated with Ni–S bonding. These results can support the presence of nickel sulfide phase. Finally, following the reviewer's suggestion, during the NiSiNWs@GrμT fabrications, we used hydrochloric acid to replace sulfuric acid for Ni etching. In this case, the plateaus at 1.4 V and 1.9 V, associated with the lithiation and delithiation of nickel sulfide residues, were disappeared in GCD profile (Supplementary Fig.S11a). All these results concreted our claim that sulfuric acid for Ni etching can develop the nickel sulfide inside of GrμT.

We thank the reviewer for the valuable suggestion which provides the opportunity to more strongly support the analysis of the plateaus/peaks at 1.4 V and 1.9 V are associated with nickel sulfide that was developed by sulfuric acid. We added Supplementary Fig. S11 & S12 and the relevant reference (ref S3) to our revised Supplementary Information.

"ref S3. An, T. C. *et al.* Interlaced NiS2-MoS2 nanoflake-nanowires as efficient hydrogen evolution electrocatalysts in basic solutions. *J. Mater. Chem. A* **4**, 13439-13443, doi:10.1039/c6ta05022k (2016)"

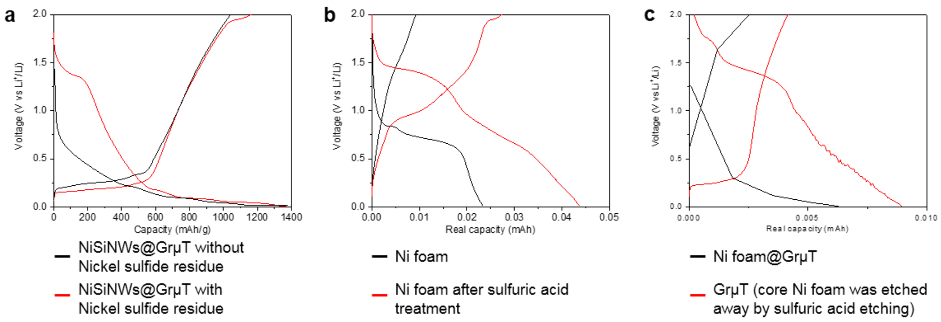

Supplementary Figure S11. Comparison of galvanostatic charge-discharge profiles for diverse samples depending on sulfuric acid treatment. a, NiSiNWs@GrμT that were underwent Ni core etching with hydrochloric acid (black line) and sulfuric acid (red line). **b,c,** Both the Ni foam (b) and Ni foam@GrμT (c) exhibited new plateaus at 1.4 V and 1.9 V in the galvanostatic charge-discharge (GCD) profiles only after sulfuric acid treatment.

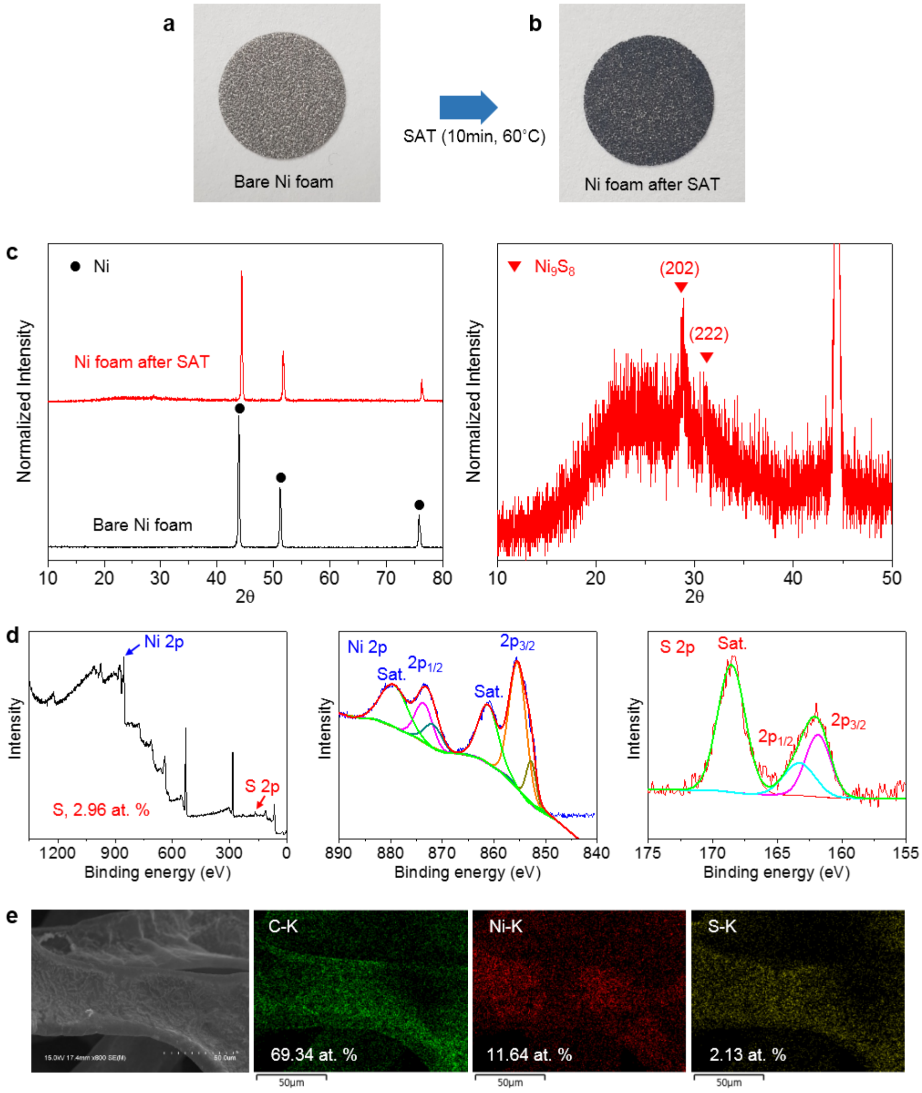

Supplementary Figure S12. Structural analysis of nickel sulfide phase on Ni foam. a,b, Photograph of bare Ni foam (a) and Ni foam after sulfuric acid treatment (SAT) (b). **c,** XRD measurements of bare Ni foam and Ni foam after SAT (left panel) and enlarged pattern of Ni

foam after SAT (right panel). **d,** X-ray photoelectron spectroscopy (XPS) spectrums of Ni foam after SAT in wide scan (left panel) and high-resolution XPS spectra of Ni 2p (middle panel) and S 2p (right panel). In Ni 2p spectra, 853.6 and 871.18 eV are assigned to $2p_{3/2}$ and $2p_{1/2}$ of Ni^{2+}, and the peaks at 855.3 and 873.3 eV are assigned to $2p_{3/2}$ and $2p_{1/2}$ of Ni^{3+}. In S 2p spectra, 161.3 and 163.4 eV are assigned to S $2p_{3/2}$ and S $2p_{1/2}$ of Ni–S bonding[S3]. **e,** SEM (left) and EDS element mapping images of Ni islands@GrμT.

REVIEWERS' COMMENTS:

Reviewer #1 (Remarks to the Author):

The authors have addressed my major concerns. I recommend publication after improving the wordings of the manuscript.

Reviewer #1 (Remarks to the Author):

Comment: The authors have addressed my major concerns. I recommend publication after improving the wordings of the manuscript.

Author Response: We thank the reviewer for the positive comment about the revised manuscript. As the reviewer recommended, we revised the final manuscript and Supplementary Information through English language editing service.
